# Identification of New *N*-methyl-piperazine Chalcones as Dual MAO-B/AChE Inhibitors

**DOI:** 10.3390/ph16010083

**Published:** 2023-01-06

**Authors:** Ashraf K. El-Damasy, Jong Eun Park, Hyun Ji Kim, Jinhyuk Lee, Eun-Kyoung Bang, Hoon Kim, Gyochang Keum

**Affiliations:** 1 Center for Brain Technology, Brain Science Institute, Korea Institute of Science and Technology (KIST), Seoul 02792, Republic of Korea; 2 Department of Medicinal Chemistry, Faculty of Pharmacy, Mansoura University, Mansoura 35516, Egypt; 3 Department of Pharmacy, and Research Institute of Life Pharmaceutical Sciences, Sunchon National University, Suncheon 57922, Republic of Korea; 4 Disease Target Structure Research Center, Korea Research Institute of Bioscience and Biotechnology (KRIBB), Daejeon 34141, Republic of Korea; 5 Department of Bioinformatics, KRIBB School of Bioscience, University of Science and Technology (UST), Daejeon 34113, Republic of Korea

**Keywords:** *N*-methyl-piperazine chalcones, monoamine oxidase, cholinesterase, kinetic study, docking simulation

## Abstract

Monoamine oxidase-B (MAO-B), acetylcholinesterase (AChE), and butyrylcholinesterase (BChE) have been considered target enzymes of depression and neurodegenerative diseases, including Alzheimer’s disease (AD). In this study, seventeen *N*-methyl-piperazine chalcones were synthesized, and their inhibitory activities were evaluated against the target enzymes. Compound **2k** (3-trifluoromethyl-4-fluorinated derivative) showed the highest selective inhibition against MAO-B with an IC_50_ of 0.71 μM and selectivity index (SI) of 56.34, followed by **2n** (2-fluoro-5-bromophenyl derivative) (IC_50_ = 1.11 μM, SI = 16.04). Compounds **2k** and **2n** were reversible competitive MAO-B inhibitors with K_i_ values of 0.21 and 0.28 μM, respectively. Moreover, **2k** and **2n** effectively inhibited AChE with IC_50_ of 8.10 and 4.32 μM, which underscored their multi-target inhibitory modes. Interestingly, compound **2o** elicited remarkable inhibitions over MAO-B, AChE, and BChE with IC_50_ of 1.19–3.87 μM. A cell-based assay of compounds **2k** and **2n** against Vero normal cells pointed out their low cytotoxicity. In a docking simulation, **2k** showed the lowest energy for MAO-B (−11.6 kcal/mol) with four hydrogen bonds and two π-π interactions. Furthermore, in silico studies were conducted, and disclosed that **2k** and **2n** are expected to possess favorable pharmacokinetic properties, such as the ability to penetrate the blood–brain barrier (BBB). In view of these findings, compounds **2k** and **2n** could serve as promising potential candidates for the treatment of neurodegenerative diseases.

## 1. Introduction

Alzheimer’s disease (AD) and Parkinson’s disease (PD) are the most prevalent neurodegenerative diseases (NDDs) that mainly affect the elderly [1]. AD and PD are characterized by heterogeneous and complex multifaceted pathologies that stem from a combination of diverse genomic, epigenomic, environmental, and metabolic factors [2]. A growing body of evidence disclosed that recovery of brain functional steadiness after a neurologic impairment is unlikely to be accomplished by focusing on a single molecular target [3]. Therefore, the research efforts for the treatment of NDDs are being oriented towards the design of multi-target directed ligands (MTDLs) [4]. However, to strike a balance between the effects of MTDLs acting at their molecular targets represents a major challenge [5].

The oxidative deamination of the biogenic amines in peripheral and central tissues is catalyzed by the flavin adenine dinucleotide (FAD) dependent monoamine oxidases (MAOs)-A and -B [6]. During the catabolism of different monoamine neurotransmitters by MAO-A/B, hydrogen peroxide and reactive oxygen species (ROS) are excessively produced as major by-products, triggering oxidative stress and neuronal cell damage associated with several NDDs [7]. Overexpression of MAO-B levels in the substantia nigra of PD patients, along with the relevance of elevated activity of MAO-B and impairment of cognitive functions in AD patients has been reported [8,9]. In this regard, MAO inhibitors are considered as up-regulating agents for neurotransmitter amines, such as dopamine and noradrenaline with neuroprotective properties [10]. Numerous investigations have reported the beneficial effects of reversible/irreversible MAO-B inhibitors in AD related therapeutic approaches [11,12]. Moreover, acetylcholinesterase (AChE) and butyrylcholinesterase (BChE) inhibitors have shown remarkable roles in the preservation of cholinergic functions and symptomatic improvement in AD [13,14]. In view of MTDLs merits, the contemporary medicinal chemists have focused their efforts, over the past few decades, to develop new chemical entities as multiple-acting MAO-B and AChE/BChE inhibitors [15,16,17,18].

**Figure 1:**
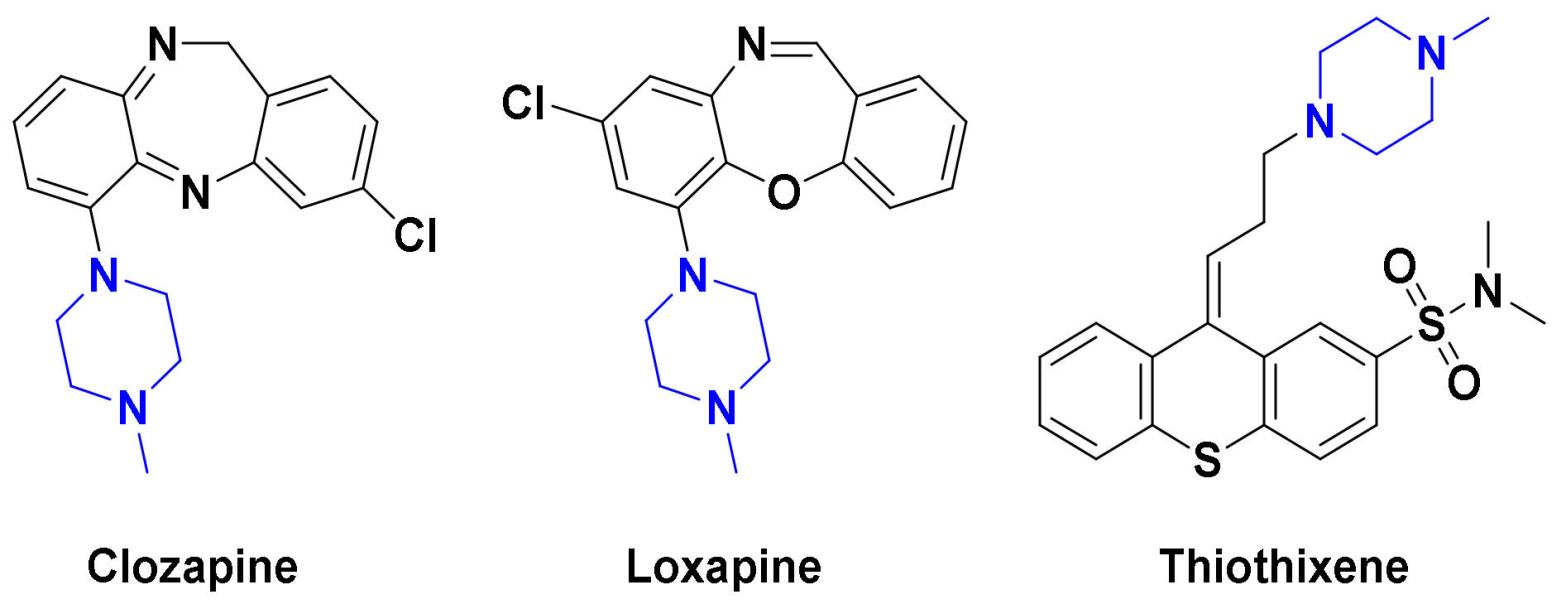
Representative examples of CNS acting FDA-approved drugs possessing *N*-methylpiperazine.

*N*-Methylpiperazine is a privileged derivative of piperazine moiety, with better lipophilic and steric characters, which renders this motif a proper structural element in maintaining the right balance of pharmacokinetic and pharmacodynamic attributes [19]. The existence of two nitrogens along with the small bulky *N*-methyl allows *N*-methylpiperazine to be engaged in various hydrophobic and charge transfer interactions with target enzymes. The favorable drug-like properties of *N*-methylpiperazine were reflected in several central nervous system (CNS) acting drugs, such as clozapine, loxapine, olanzapine, trifluoperazine, and thiothixene (Figure 1) [20]. Furthermore, *N*-methylpiperazine was introduced in a number of anticancer kinase inhibitors, to improve water solubility and/or target affinity, such as bosutinib, ponatinib, nintedanib, brigatinib, and gilteritinib [21]. Moreover, recent studies revealed that the replacement of one or both nitrogen atoms in the piperazine ring with different structural motifs could significantly improve MAO-B, and AChE inhibitions [22,23,24]. Based on this perspective, insertion of *N*-methylpiperazine into the proper scaffolds of certain MAO-B inhibitors would be expected to afford dual MAO-B/AChE inhibitions.

Further, chalcone, 1,3-diphenylprop-2-en-1-one, represents a substantial flexible scaffold for the design of selective MAO-B and/or AChE inhibitors [25], where the existence of propenone (α-β unsaturated ketone) and three rotatable bonds in chalcones can furnish various binding orientations with multiple targets [26]. Recent studies pointed out that conjugation of certain FDA-approved drugs, such as rivastigmine and donepezil, with chalcone scaffold led to generation of multifunctional MAO-B/ChE inhibitors, such as compound **I** with potent neuroprotective properties for AD [15]. Installing different electron-pulling and pushing groups on the aryl/heteroaryl rings of chalcones can modulate the electrophilic character of the Michael acceptor propanone as exemplified by chalcones **II** and **III** [27,28], where the introduction of lipophilic halogens (F, Cl, Br)/electron donation groups (-OMe, -N(Me)_2_) onto the phenyl B ring of chalcones afford highly selective MAO-B inhibitors such as compound **IV** [29,30]. On the other hand, the existence of proper alkylamino moieties on the ketone linked ring A (i.e., compound **V**) was found to be favorable for achieving AChE inhibitory activity [31,32].

**Figure 2:**
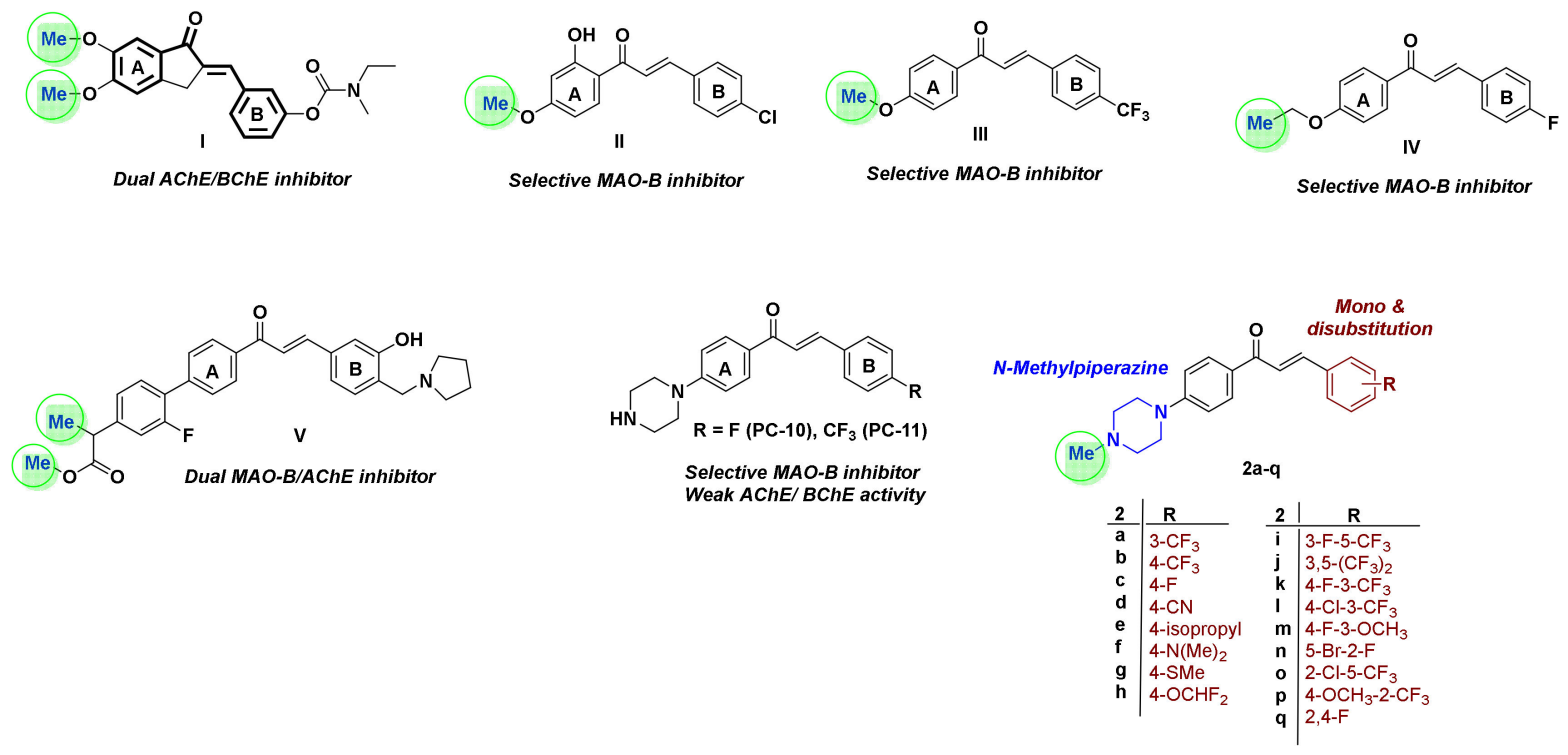
Representative examples of reported chalcones as selective MAO-B inhibitors or dual MAO-B/AChE or BChE inhibitors, and chemical structure of the target compounds **2k**–**2q**.

As part of our continued endeavors to develop novel chemical entities as potent MAO-B/or AChE inhibitors, we have recently reported compounds **PC-10** and **PC-11** as piperazine featuring chalcones with promising selective MAO-B inhibitors (Figure 2) [33]. Compounds **PC-10** and **PC-11** showed IC_50_ values of 0.65 and 0.71 μM, respectively, against MAO-B, with selectivity index (SI) of 48.3 and 49.2 over MAO-A. Furthermore, kinetics study of **PC-10** and **PC-11** revealed their competitive mode of MAO-B inhibition with inhibition constant (K_i_) of 0.63 and 0.53 μM, respectively. However, these molecules exerted modest activity towards both AChE and BChE with IC_50_ values of 26.3–28.0 and 36.2–36.4 μM, respectively. Motivated by such findings, as well as the aforementioned considerations highlighting the significance of terminal methyl group (Figure 2), we pursued further structural modifications on compounds **PC-10** and **PC-11** aiming at design of dual MAO-B/AChE inhibitors with improved potency. Thus, in the current study, seventeen *N*-methylpiperazine chalcones were designed, synthesized, and biologically tested against MAO-A/B and AChE/BChE. We expected that insertion of the small bulky methyl group on the terminal amine of piperazine would impart AChE inhibitory activity for the target molecules. In addition, diverse mono- and di-substituted phenyl rings, with various lipophilic characters, were investigated to construct a reliable structure activity relationship (SAR) study. To the best of our knowledge, this is the first report harnessing *N*-methylpiperazine moiety to afford dual MAO-B/AChE chalcone inhibitors.

## 2. Results and Discussion

### 2.1. Synthesis of Compounds

**Scheme 1:**
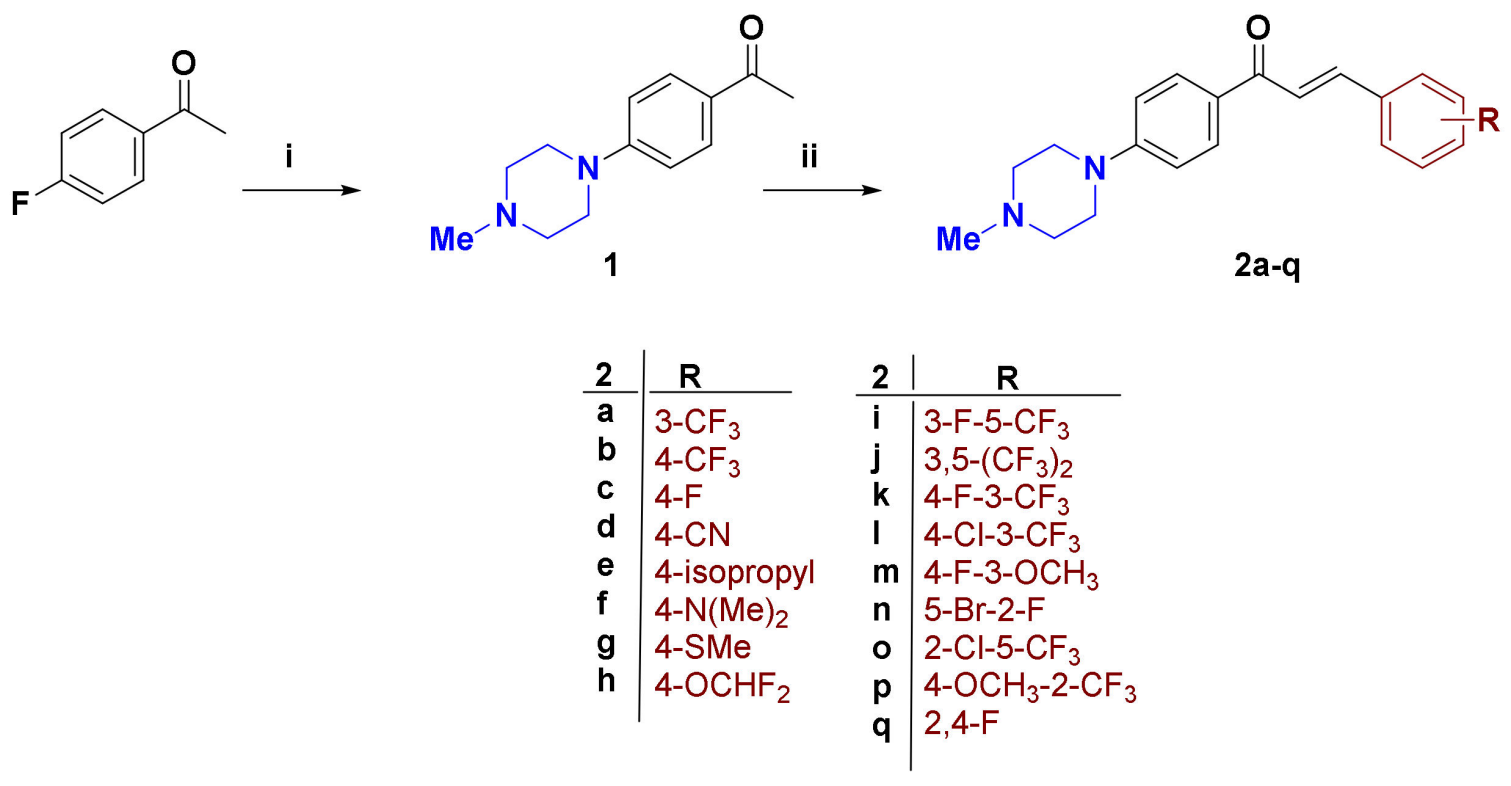
Reagents and reaction conditions: (i) 4-Methylpiperazine, neat, 140 °C, 12 h, 84%; (ii) Benzaldehyde derivative, 40% ethanolic sodium hydroxide, 95% ethanol, rt, 0.5–4 h, 59–92%.

As illustrated in Scheme 1, the synthesis of the target 4-methylpiperazine-containing α,β-unsaturated ketones **2a**–**q** was commenced with preparation of 4-methylpiperazine acetophenone 1 as the key precursor. Heating of 4-fluoroacetophenone with 4-methylpiperazine at 140 °C under neat conditions afforded compound **1** in 84% yield [34]. The Claisen–Schmidt condensation of 1 was with equimolar amounts of various mono/di-substituted benzaldehydes in 40% alcoholic NaOH at room temperature, and furnished the target molecules **2a**–**q** in good yield. The ^1^H NMR charts of the final compounds showed the 4-methylpiperazine ring protons *N*-CH_2_ resonating at 3.42 and 2.57 ppm as triplets, as well as *N*-CH_3_ protons as a singlet at 2.36 ppm. The large coupling constant (*J* = 15.6 Hz) value of H_α_ and H_β_ enone protons confirmed the *trans* (*E*) configuration of the 4-methylpiperazine chalcones. The spectra were provided in Supplementary (Figures S1–S3).

### 2.2. Inhibitory Activities against MAO-A/B, AchE, and BChE

#### 2.2.1. Overview of the Activity

Chalcone is a well-known privileged scaffold as a MAO inhibitor, particularly selective towards MAO-B [35]. From this background, a plethora of structurally diverse chalcone derivatives has been synthesized by our team, and their MAO-A/B inhibition profiles have been reported [33,36,37]. The results revealed that the best members in those chalcone derivatives had IC_50_ spanning from 0.0021 to 0.65 μM against MAO-B [33,36,37].

In this study, a set of seventeen *N*-methyl-piperazine chalcone derivatives were synthesized and their inhibitory activities were evaluated against the target enzymes. In the preliminary inhibition assay at 10 μM concentration, all compounds exerted <50% of residual activity for MAO-B, except **2j**, whereas all compounds showed >50% of residual activity for MAO-A, except **2p** (Table 1). Compound **2k** (3-trifluoromethyl-4-fluorinated derivative) elicited the highest selective inhibition against MAO-B with IC_50_ of 0.71 μM and selectivity index (SI) of 56.34, followed by the 2-fluoro-5-brominated derivative **2n** (IC_50_ = 1.11 μM, SI = 16.04) (Table 2, Figure S4). Furthermore, most of the tested chalcone derivatives displayed <50% of residual activity for AChE at 10 μM, except **2g**, **2i**, **2j**, and **2l**, whereas most of compounds showed >50% of residual activity for BChE at 10 μM, except **2c**, **2e**, and **2n–p**. Among them, **2b** had the highest AChE inhibition with IC_50_ of 2.26 μM and SI (ChE) of 5.92, followed by **2d** with IC_50_ of 2.38 μM, and SI (ChE) of 10.38. Concerning BChE, **2o** stood out as the most potent member with IC_50_ of 1.19 μM. Remarkably, all the compounds exerted selective dual MAO-B/AChE inhibition, except **2o**, disclosing that *N*-methyl-piperazine chalcone derivatives could be expected as multi-target inhibitors for AD.

#### 2.2.2. SAR

Regarding the monosubstituted chalcones **2a**–**h**, installing an electron withdrawing group (EWG) on the terminal phenyl moiety is favorable for MAO-B inhibition, as observed with **2a**–**d** (IC_50_ = 2.20–3.10 μM). The two positional isomers **2a** and **2b** showed comparable activity for MAO-B, however the *para*-trifluoromethylphenyl derivative **2b** elicited 2.5-fold better inhibitory activity against AChE (IC_50_ = 2.26 μM) than its corresponding congener **2a**. Replacing the –CF_3_ group in **2b** with fluorine (**2c**) retained MAO-B inhibitory potency, while leading to ~4.6-fold reduction in AChE inhibition (**2c**, IC_50_ = 10.3 μM), pointing out that the trifluoromethyl moiety is favorable for AChE suppression. Such a finding was further emphasized by compounds **2g** and **2h**, which are equipotent against MAO-B, while the 4-difluoromethoxy member **2h** exerted better potency for AChE (IC_50_ = 5.82 μM) than its corresponding methylthio derivative **2g** (IC_50_ = 12.41 μM). Replacing the carbon of isopropyl substituted chalcone **2e** (IC_50_ = 8.36 μM) with nitrogen (**2f**) resulted in ~2.5-fold augmentation in MAO-B inhibition (IC_50_ = 3.29 μM). Upon comparing the activity of **2b**, **2c**, and **2f** with their corresponding methyl lacking piperazine congeners **PC11**, **PC10**, and **PC5** [33], respectively. It was found that incorporation of the methyl moiety at piperazine decreased the MAO-B inhibition to certain extent, however it significantly improved the AChE inhibitory potency. For example, the 4-methylpiperazine chalcones **2b** and **2f** exerted IC_50_ values of 2.26 μM and 3.03 against AChE, while their relevant desmethyl analogs **PC11** and **PC5** showed IC_50_ values of 26.3 μM and >10 μM [33], respectively. Except **2c** and **2g**, all monosubstituted chalcones elicited IC_50_ values < 6.0 μM towards AChE, standing as promising dual MAO-B/AChE inhibitors. Among them, the *para*-cyanophenyl derivative **2b** showed the best activity with equipotency towards MAO-B/AChE (IC_50_ = 2.2 μM), along with highest SI (18.18).

**Figure 3:**
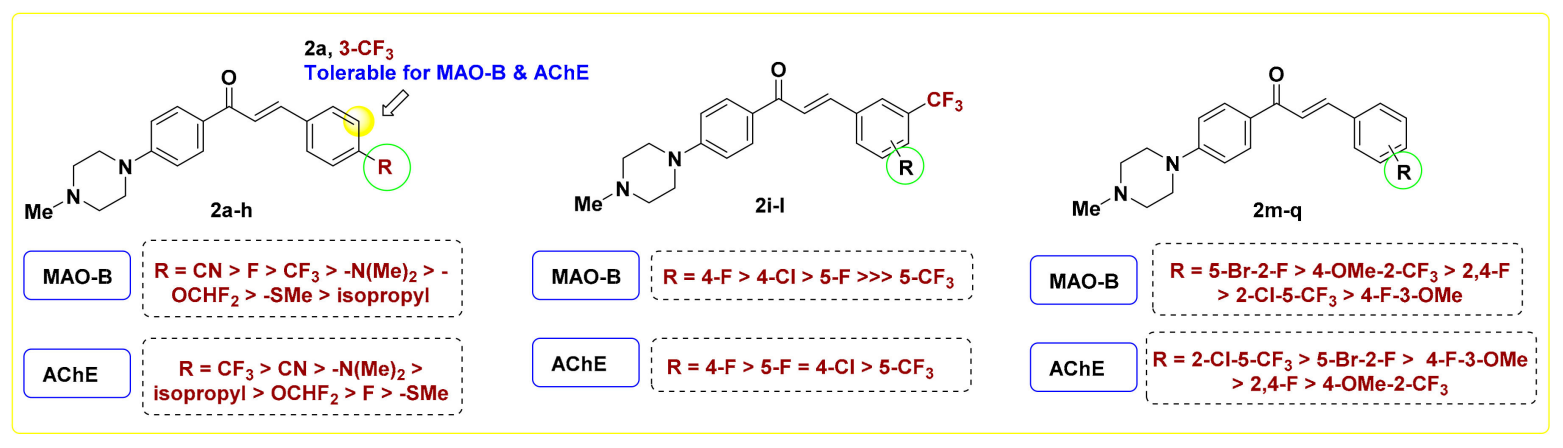
SAR analysis of the target compounds **2a**–**q** for MAO-B/AChE inhibition.

Referring to the disubstituted derivatives **2i**–**q**, it was noticed that the existence of a four-halogen moiety (F/Cl) adjacent to *meta*-trifluoromethyl is optimal for MAO-B inhibition. For example, compound **2k** bearing 3-trifluoromethyl-4-fluorophenyl showed superior potency against MAO-B (IC_50_ = 0.71 μM) compared to its 3,5-positional isomer **2i** (IC_50_ = 2.33 μM). Upon comparing the 4-halogenated-3-trifluoromerthylphenyl derivatives **2k** and **2l**, it was found that **2k** is relatively more potent than its corresponding 4-chloro congener **2l** over MAO-B, AChE, and BChE. Such a finding is in agreement with the recent report of Rullo et al., which underscored the importance of fluorinated motifs in achieving sound MAO-B inhibition [38]. Worth noting is that replacing fluorine in **2i** (IC_50_ = 2.33 μM) with a trifluoromethyl group (**2j**) led to dramatic drop in MAO-B inhibition (IC_50_ = 15.70 μM). In addition, the introduction of a 3-methoxy group, in place of the 3-trifluoromethyl motif of **2k**, afforded the chalcone **2m** with significantly reduced potency for MAO-B (IC_50_ = 6.76 μM), yet with reasonable inhibition towards AChE and BChE. Upon inspection of the activity of 2,5-disubstituted chalcones **2n** and **2o**, it was observed that compound **2o**, the positional isomer of **2l**, displayed a distinct multi-target inhibitory effect over MAO-B, AChE, and particularly BChE (IC_50_ = 1.19 μM). As an exception to the noticed dual selective MAO-B/AChE inhibition of this set of 4-methylpiperazine, the 2,4-difluoro member **2p** elicited moderate MAO-A potency (IC_50_ = 6.67 μM) with a low SI value of 3.06. In view of these biochemical assay results and as illustrated in Figure 3, it is evident that both the nature and position of the substituent installed on terminal phenyl ring possess critical roles in modulating the inhibitory potency towards MAO-A/B and AChE/BChE.

### 2.3. Inhibition Pattern Analysis of ***2k*** and ***2n***

**Figure 4:**
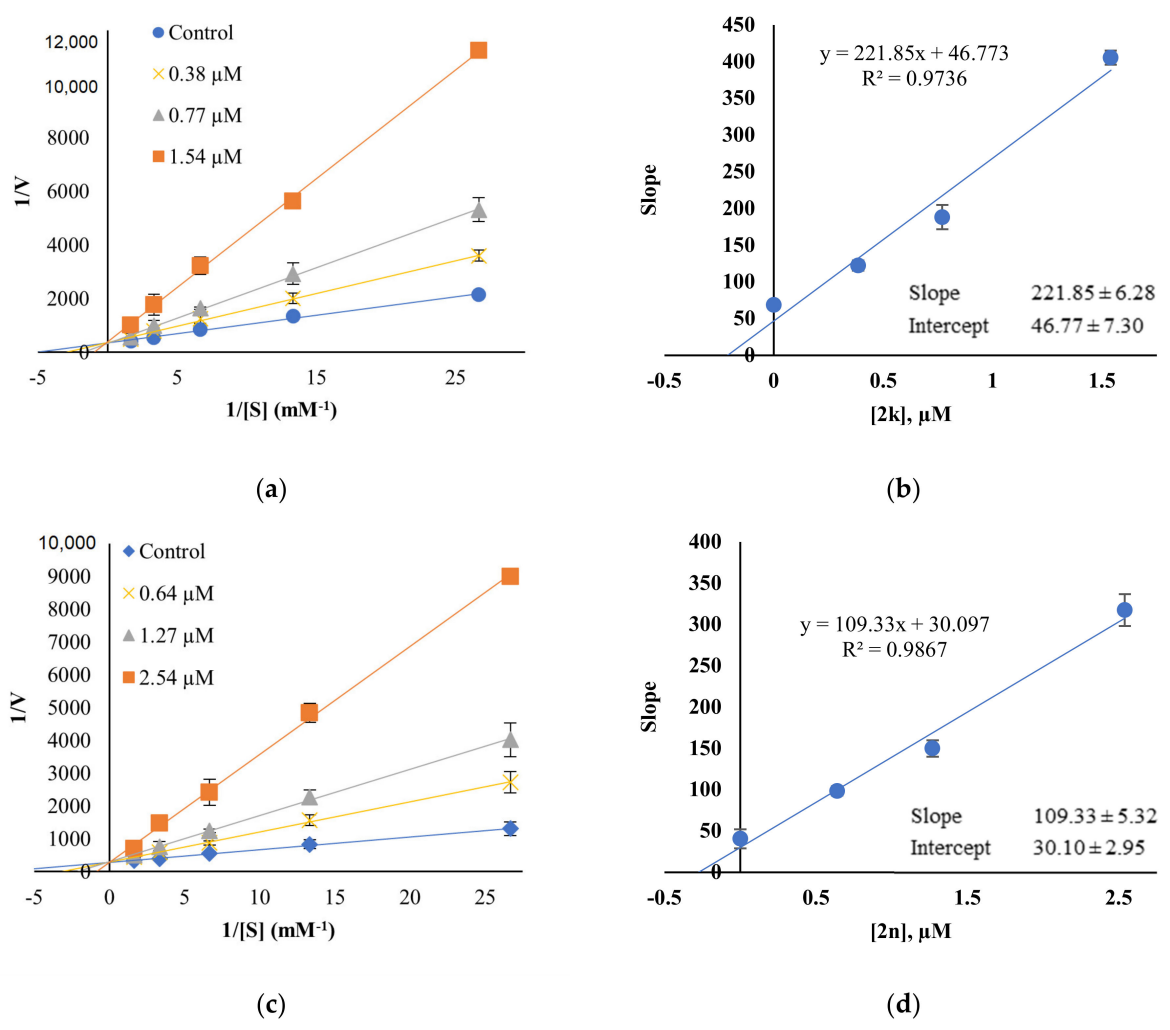
Lineweaver–Burk plots (**a**,**c**) and their secondary plots (**b**,**d**) of **2k** and **2n** for MAO-B, respectively. Five concentrations of benzylamine used were 0.0375, 0.075, 0.15, 0.3 and 0.6 mM, respectively, and three concentrations of inhibitor were ~1/2×, 1×, and 2 × IC_50_: 0.38, 0.77, and 1.54 µM for **2k**; 0.64, 1.27, and 2.54 µM for **2n**. The K_m_ value for MAO-B was 0.20 mM.

A kinetic study of the most active compounds **2k** and **2n** against MAO-B was performed to analyze the inhibition patterns using Lineweaver–Burk plots, and their Ki values were determined using their secondary plots. The Lineweaver–Burk plots of **2k** and **2n**, matched in Y-axis, showed that Ki values were 0.21 ± 0.03 μM and 0.28 ± 0.01 μM, respectively, which points out that both compounds are competitive inhibitors of MAO-B (Figure 4).

**Figure 5:**
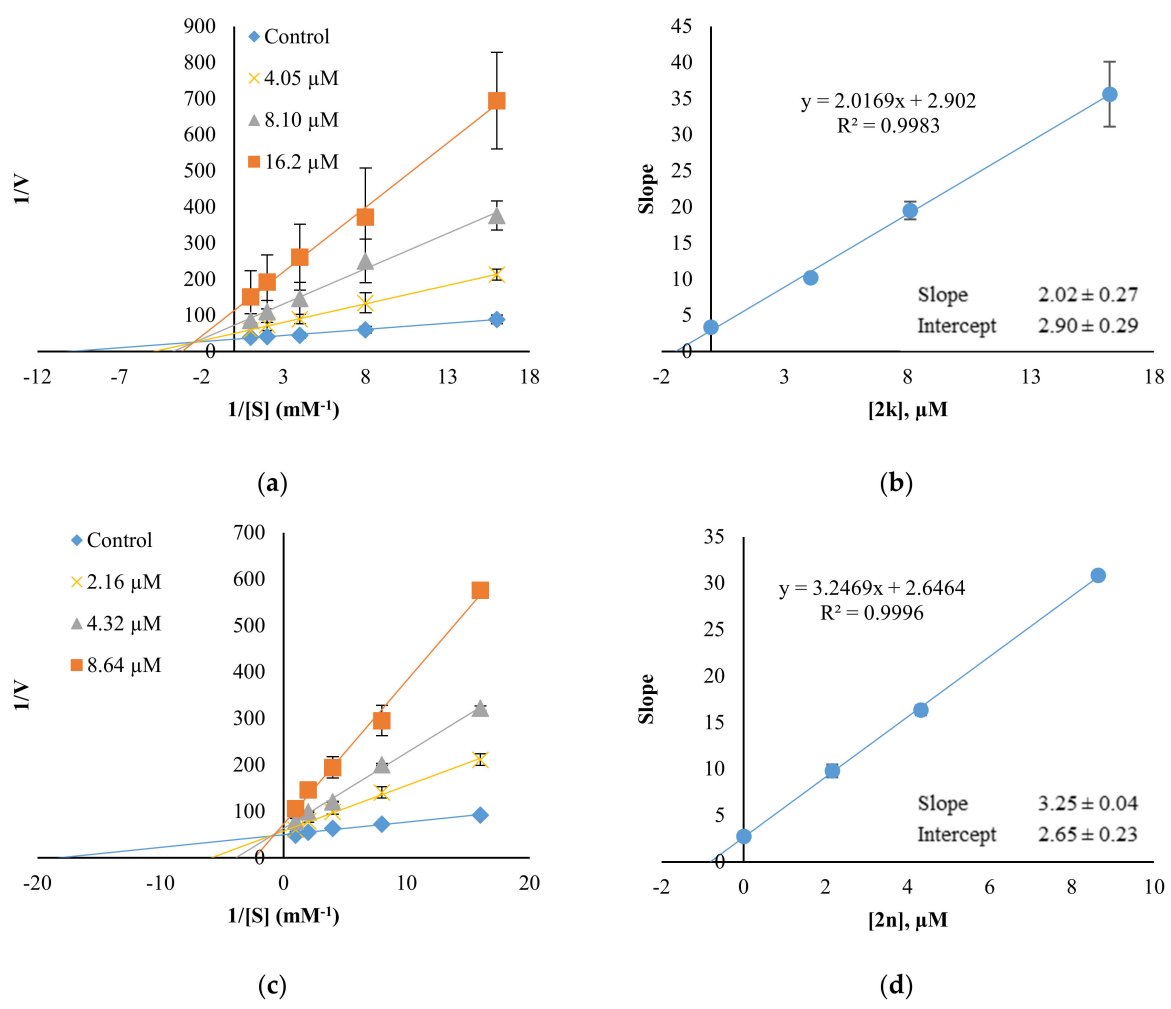
Lineweaver–Burk plots (**a**,**c**) and their secondary plots (**b**,**d**) of **2k** and **2n** for AChE. Five concentrations of ATCI were used; 0.0625, 0.125, 0.25, 0.5 and 1.0 mM, respectively, and three concentrations of inhibitor were ~1/2×, 1×, and 2 × IC_50_: 4.05, 8.10, and 16.20 µM for **2k**; 2.16, 4.32, and 8.64 µM for **2n**. The K_m_ value for AChE was 0.075 mM.

Since **2k** and **2n** displayed AChE inhibition with IC_50_ of 8.10 and 4.32 µM, respectively, kinetics for AChE were also performed (Figure 5). **2k** and **2n** were found to be mixed type inhibitors for AChE, showing contact points in second quadrants with *K_i_* values of 1.44 ± 0.33 and 0.82 ± 0.08 µM, respectively.

**Figure 6:**
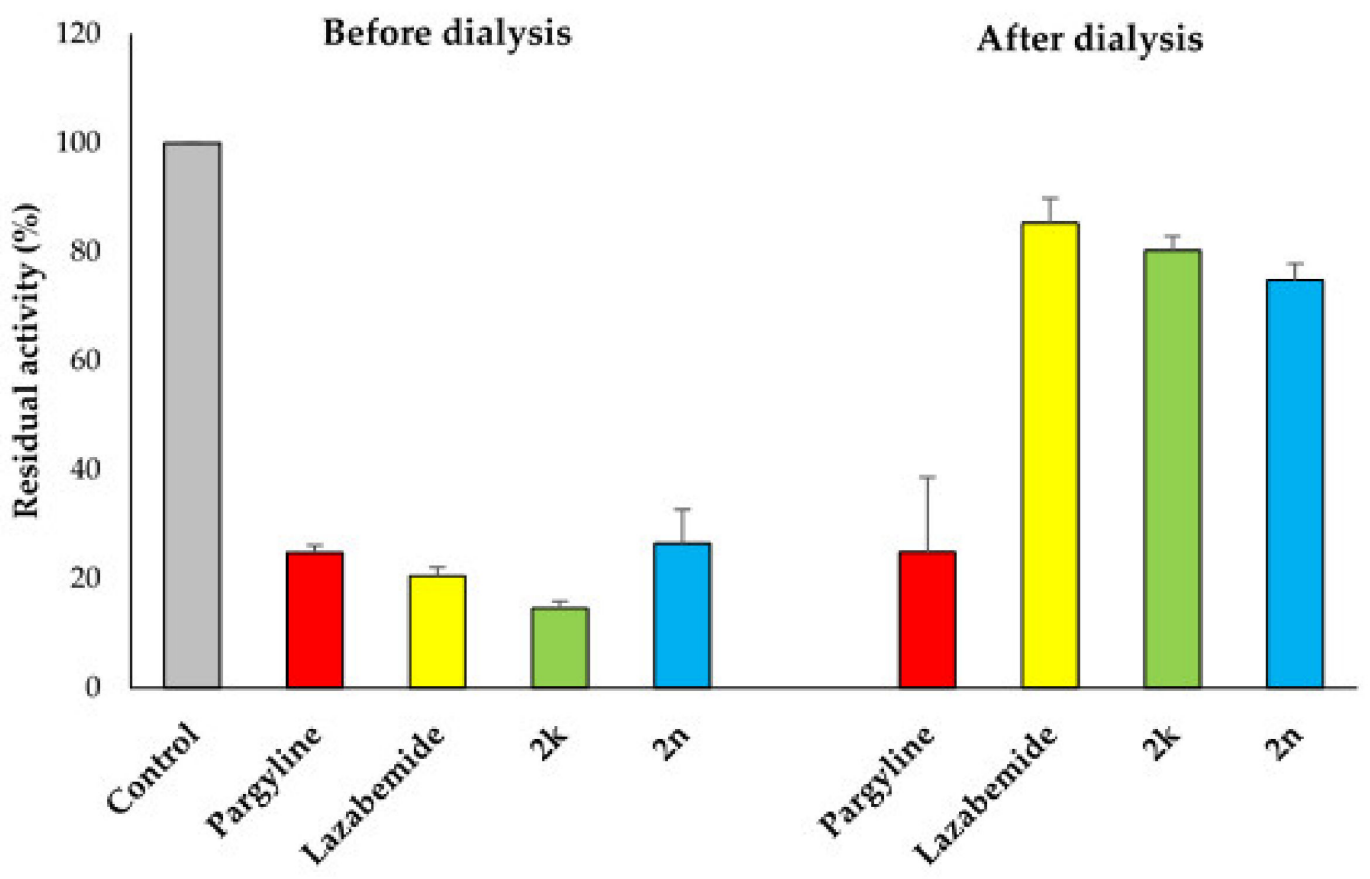
Reversibility tests of **2k** and **2n** for MAO-B using dialysis method. MAO-B was treated with ~2 × IC_50_ of pargyline (0.28 µM), lazabemide (0.22 µM), **2k** (1.16 µM), and **2n** (2.54 µM) before dialysis for 6 h. The residual activities were determined before and after dialysis to confirm the reversibility pattern using enzyme assay described in Materials and Methods.

In reversibility tests of **2k** and **2n** for MAO-B using the dialysis method, their residual activities were efficiently recovered from 14.63% and 26.47% before dialysis, respectively, to 80.26% and 74.83% after dialysis, respectively (Figure 6). In references, pargyline, an irreversible MAO-B inhibitor, displayed no recovery of the residual activity, i.e., 24.81% to 24.88%, whereas lazabemide, a reversible MAO-B inhibitor, showed efficient recovery of the residual activity, i.e., from 20.53% to 98.78%. From these results, it is concluded that **2k** and **2n** are reversible MAO-B inhibitors.

In a previous study, piperazine chalcone derivatives were also analyzed for their inhibitory activities against MAO-B [33], and *p*-fluorinated piperazine chalcone derivative **PC10** showed the highest reversible competitive inhibition for MAO-B with an IC_50_ value of 0.65 µM (K_i_ = 0.50 µM). On the other hand, **2k**, the most potent MAO-B inhibitor in this study, also showed reversible competitive inhibition with IC_50_ of 0.71 µM (K_i_ = 0.21 µM), followed by **2n** (IC_50_ = 1.11 µM, K_i_ = 0.28 µM). Interestingly, **2k** and **2n** elicited effective inhibition against AChE with IC_50_ values of 8.10 and 4.32 µM, respectively, in contrast to **PC10** which showed a moderate inhibition with IC_50_ of 28.0 µM [33]. **2k** and **2n** showed mixed type inhibition for AChE with K_i_ values of 1.44 and 0.82 µM, respectively; however, there were no kinetics data of **PC10** against AChE [33]. We previously reported about AChE inhibition of morpholine-based chalcones, which have a similar structure to the piperazine chalcone; however, the lead compounds **MO5** and **MO9** exerted competitive and non-competitive inhibition, not mixed-type, for AChE with K_i_ values of 2.52 µM and 7.04 µM, respectively, which are remarkably lower than those of **2k** and **2n** [39]. These findings suggest that *N*-methyl piperazine chalcones possess comparable or better K_i_ values for MAO-B than their corresponding piperazine chalcones, as well as possessing higher affinities with multi-target inhibitions for targeting AD.

### 2.4. Cytotoxicity Study of ***2k*** and ***2n***

**Figure 7:**
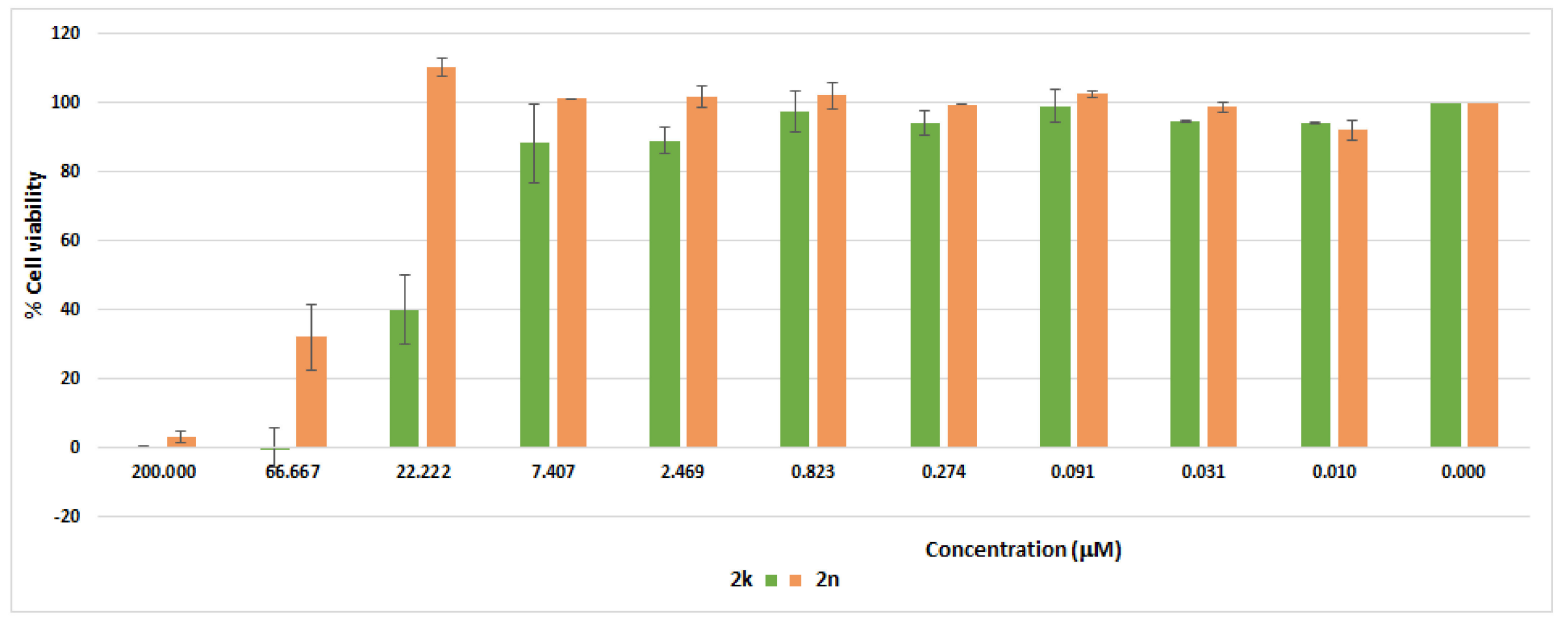
Cytotoxicity profile of compounds **2k** and **2n** over normal Vero cells. The cells were incubated with various concentrations of **2k** and **2n** for 24 h (0.01, 0.03, 0.09, 0.27, 0.82, 2.47, 7.41, 22.2, 66.7, and 200 µM) and MTT assays were conducted. Data are expressed as the mean ± SD of duplicate assay.

The cytotoxicity profile of **2k** and **2n** on the cellular viability of VERO normal cells was investigated at ten different concentrations (0.0102–200 µM), using MTT (3-(4,5-dimethylthiazol-2-yl)-2,5-diphenyltetrazolium bromide) assay. As depicted in Figure 7, compounds **2k** and **2n** were non-toxic to normal VERO cells with IC_50_ values of 19.93 µM and 62.04 µM, respectively, pointing out their suitability as potential therapeutic candidates.

### 2.5. Computational Studies

#### 2.5.1. Docking Simulations of **2k** and **2n**

**Figure 8:**
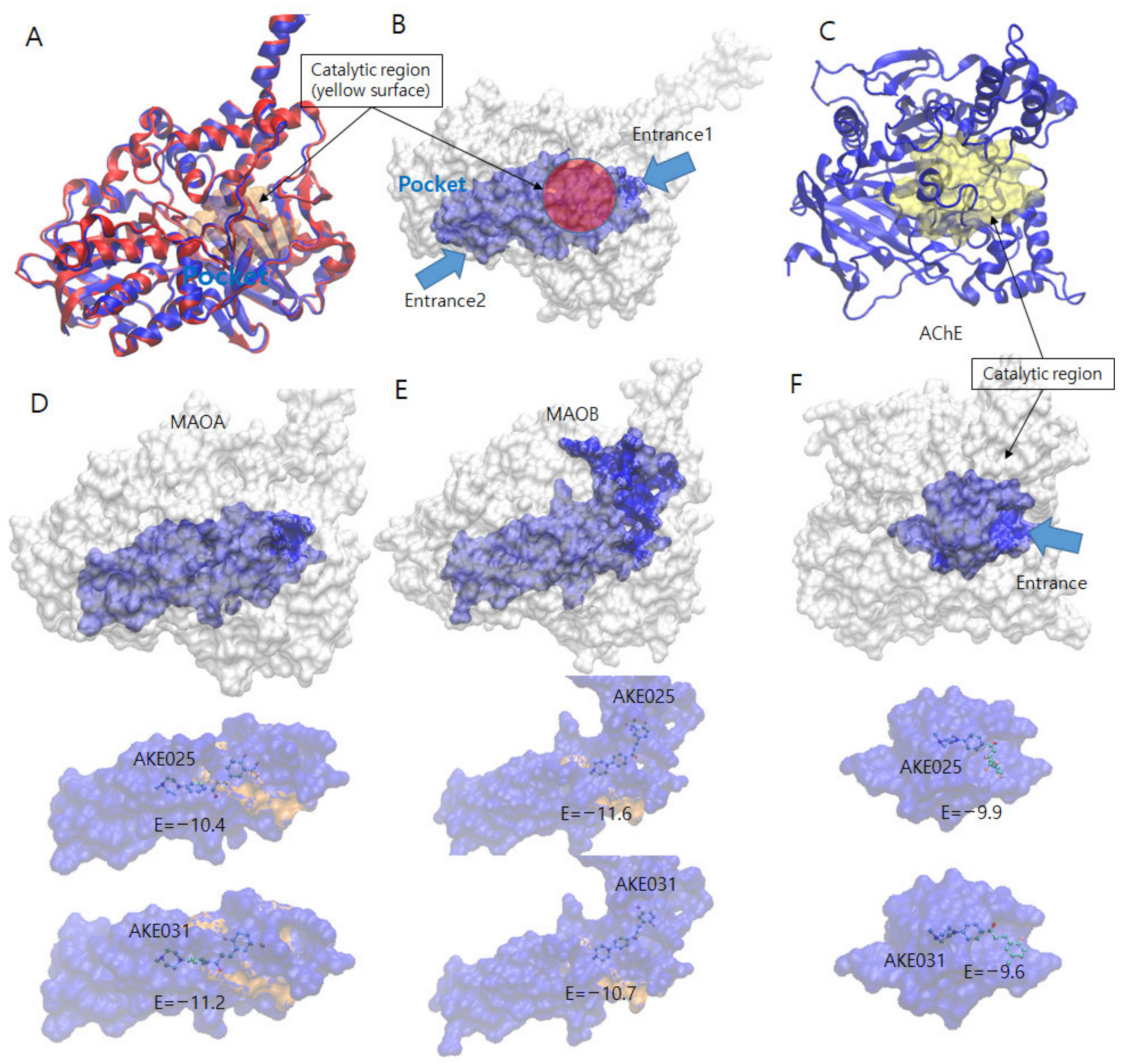
Molecular docking of **2k** and **2n** on MAO-A, MAO-B, and AChE. (**A**) Superimposed MAO family structure. Both structures are drawn by Cartoon model shaded by blue (MAO-A) and red color (MAO-B). The catalytic regions are drawn by transparent yellow surface model in the center. (**B**) Pocket and catalytic region of MAOs. The protein model is drawn by white surface model. MAO family structure has large pocket (blue surface model) in the center and connected to the catalytic region (red circle) in the center. The pocket has two entrances toward the catalytic regions (two blue arrows). (**C**) AChE protein structure drawn by blue cartoon model and yellow transparent surface model (catalytic region). The compound entrances toward the catalytic region are indicated by blue arrows. Docking simulation results of compounds **2k** and **2n** into MAO-A (**D**), MAO-B (**E**), and AChE (**F**). The MAOs structures are sketched by white sphere model and the binding pockets that exist on the center are shaded by blue color. The below panels show the binding pocket shade by transparent blue spheres for the existing compound to show. The binding catalytic regions are shown by transparent yellow colors. The compounds are bound in the pocket and near the catalytic region.

**Figure 9:**
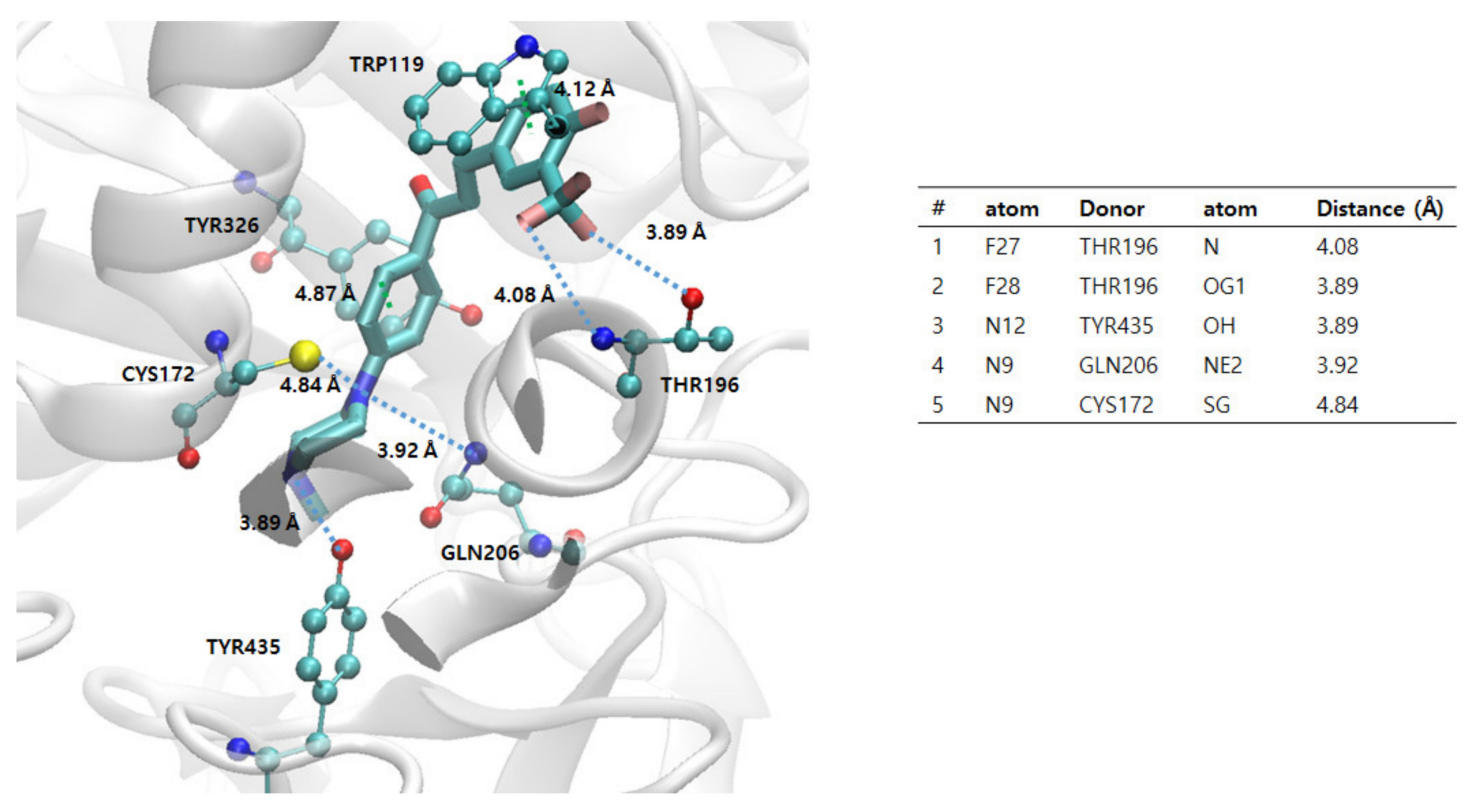
The putative 3D binding mode of **2k** with MAO-B. The π-π binding interactions are indicated by green thick dash lines with the distance between two centroids of the rings, and the hydrogen bonds are depicted by blue thick dash lines with the distance between donor and acceptor atoms. The detailed analyses including donor and acceptor atoms and the distance are tabulated on right of the Figure.

Docking simulations of the best two members **2k** and **2n** with MAO-A (pdb code: 2Z5X), MAO-B (pdb code: 4A79), and AChE (pdb code: 6O4W) were performed using AUTODOCK-VINA [40]. The catalytic sites of MAO-A, MAO-B, and AChE are highlighted, as depicted in Figure 8A, 8B, and 8C, respectively. Six combinations of docking simulations were performed (MAO-A-**2k** and MAOA-**2n** in Figure 8D, MAO-B-**2k** and MAO-B-**2n** in Figure 8E, and AChE-**2k** and AChE-**2n** in Figure 8F). The docked compounds were clustered based on their position on the protein. Each top lowest energy conformation in the catalytic region of the three proteins is tabulated in Table 3. Each lowest energy compound near the catalytic region is sketched, with their docking energies in Figure 8D–F. Among the generated conformations, compound **2k** has the lowest energy with MAO-B (−11.6 kcal/mol), indicating the tightest binding which is consistent with the experimental IC_50_ value. That tight binding stems from the existence of several hydrogen bonding interactions (blue dash lines), as shown in Figure 9. The main key residues are THR196, GLN206, and TYR435, and the crucial interacting functional groups in **2k** are CF_3_ and two nitrogen atoms in piperazine. Because the side chain of THR196 can rotate freely and stay in three conformers, *Gauche+* (60° of torsional angle in N-CA-CB-OG1), *Gauche-* (−60°), and *trans* (180°), the hydrogen bonds of the donor, OG1 in THR196 could enhance its binding affinity toward the –CF_3_ of **2k**. The nitrogen atom in the backbone of THR196 can also engage in hydrogen bonds with the –CF_3_. Two terminus atoms in the side chain (–NE_2_ for GLN206 and -OH for TYR435) can share binding interactions toward the two nitrogen atoms in the piperazine of **2k**. Although the number of hydrogen bonds in MAO-B-**2k** is four hydrogen bonds, the MAO-A-**2k** also has four hydrogen bonds. However, MAO-B-**2k** possesses lower binding energy and the number of π-π interaction is two, as shown in Figure 8, which is higher than one in MAO-A-**2k**. Two π-π interactions are found between (1) TYR326 and the central planar ring, and (2) TRP119 and the fluorine aromatic ring. In particular, the first π-π interaction between TYR326 and the central ring of **2k** plays an important role in inhibitory selectivity of MAO proteins. The MAO-B has TYR326, and the corresponding residue in MAO-A is ILE335. The structural difference confirms that the inhibitor selectivity of MAO-A and MAO-B is caused by π-π interaction [41]. The docking of compounds **2k** and **2n** on AChE had higher binding energies than the MAO series. However, AChE showed a higher number of hydrogen bonds and the same number of π-π interactions. Therefore, both compounds **2k** and **2n** could have tighter binding on AchE than the MAO-A protein. As revealed from the docking simulation results, compound **2k** was the most favorable inhibitor in MAO-B in terms of the lowest binding energy and higher number of hydrogen bonds along with π-π interactions, and these results are in good agreement with the IC_50_ or K_i_ experiment results. In AChE inhibition, K_i_ results were not in accordance with the results of the docking data; 2n (K_i_ = 0.82 uM) bound more tightly on AChE than 2k (K_i_ = 1.44 uM), but differences in the docking data of AChE were smaller than those of MAO-B. This discrepancy might come from the difference in inhibition modes; AChE inhibition by 2k and 2n was mixed type with a different degree, while MAO-B inhibition was competitive. In addition, docking data were calculated based on a combination of various factors such as hydrogen bonding, electrostatic bonding, van der Waals forces, dissolvent effects, and the flexibilities of compounds [42]. Therefore, mixed inhibition degrees and microelement environments of the binding sites might influence the experimental K_i_ values, though docking values were not exactly in line with the K_i_ values.

#### 2.5.2. Pharmacokinetic Prediction of **2k** and **2n**

As a part of our study, ADME prediction for compounds **2k** and **2n** was performed. **2k** and **2n** were predicted to have high gastrointestinal absorption, blood–brain barrier (BBB) permeability, and no substrates of P-glycoprotein (P-gp). **2k** inhibited cytochrome P450 (CYP) 2C19, 2D6, and 3A4, whereas **2n** inhibited CYP 2C19, 2C9, and 2D6 (Table 4). Furthermore, **2k** and **2n** were predicted to have no violations for Lipinski rules (Table 5) [44]. Accordingly, **2k** and **2n** would be expected to possess favorable pharmacokinetic properties for the treatment of neurodegeneration disorders.

## 3. Materials and Methods

### 3.1. Chemistry

General: All solvents and reagents were obtained from commercial suppliers and were used directly without further purification. The reaction progress was monitored on a TLC plate (Merck, silica gel 60 F_254_, Darmstadt, Germany). Melting points were measured using OptiMelt MPA100 melting point apparatus and were uncorrected. FT-IR spectra were scanned using Frontier IR SP8000 MIR DTGS / KBr / Al / Ext beams with Spotlight 200i microscopy (PerkinElmer Inc., MA, USA). ^1^H and ^13^C NMR spectra were recorded on a Bruker Advance 400 MHz spectrometer, using deuterated chloroform. Chemical shifts (δ) are given in parts per million (ppm) upfield from tetramethylsilane (TMS) as an internal standard, and s, d, t, and m are presented as singlet, doublet, doublet triplet and multiplet, respectively. Coupling constants (*J*) are reported in hertz (Hz). High resolution mass spectra (HRMS) were recorded on a Waters Acquity UPLC/Synapt G2 QTOF MS or JMS 700 (Jeol, Tokyo, Japan) mass spectrometer.

### 3.2. Synthesis

#### 3.2.1. 1-(4-(4-Methylpiperazin-1-yl)phenyl)ethan-1-one (**1**) [34]

A mixture of 1-(4-fluorophenyl)ethan-1-one (2.5 g, 18 mmol) and 4-methylpiperazine (9 g, 90 mmol) was stirred and heated in a sealed tube at 140 °C for 12 h. The reaction mixture was cooled down to room temperature and poured into ice water. The precipitated solid was filtered under vacuum and dried to afford the title compound as a pure yellow solid, 3.5 g (84.2% yield). ^1^H and ^13^C NMR data are consistent with the reported in literature.

#### 3.2.2. General Procedure for Synthesis of Compounds **2a**–**q**

To a solution of compound **1** (250 mg, 1.15 mmol) and the appropriate aromatic aldehyde (1.15 mmol) in 95% ethanol (5 mL), 40% alcoholic sodium hydroxide (1 mL) was added. The reaction mixture was stirred at rt for 0.5–4 h, and the formed precipitate was filtered, washed with cold ethanol, dried and crystallized from ethanol to afford the target compounds, as a yellow amorphous powder, in their pure form.

1. (*E*)-1-(4-(4-methylpiperazin-1-yl)phenyl)-3-(3-(trifluoromethyl)phenyl)prop-2-en-1-one (**2a**)

Yield 89%; mp = 162–163 °C, IR (KBr, cm^−1^): 2798, 1654, 1608, 1584, 1252, ^1^H NMR (400 MHz, CDCl_3_) δ 7.98 (d, *J* = 9.0 Hz, 2H), 7.84 (s, 1H), 7.77–7.73 (m, 2H), 7.60–7.56 (m, 2H), 7.49 (t, *J* = 7.7 Hz, 1H), 6.88 (d, *J* = 9.0 Hz, 2H), 3.37 (t, *J* = 5.1 Hz, 4H), 2.52 (t, *J* = 5.1 Hz, 4H), 2.32 (s, 3H); ^13^C NMR (100 MHz, CDCl_3_) δ 187.31, 154.22, 141.04, 136.17, 131.47, 131.16, 130.79, 129.42, 127.73, 126.33 (d, *J* = 3 Hz), 124.49 (d, *J* = 4 Hz), 123.91 (d, *J* = 271 Hz), 123.68, 113.44, 54.69, 47.13, 46.11; HRMS (ESI-TOF) m/z calcd for C_21_H_22_F_3_N_2_O [M+H]^+^: 375.1684, found: 375.1701.

2. (*E*)-1-(4-(4-methylpiperazin-1-yl)phenyl)-3-(4-(trifluoromethyl)phenyl)prop-2-en-1-one (**2b**)

Yield 85%; mp = 190–191 °C, IR (KBr, cm^−1^): 2800, 1652, 1606, 1586, 1256, ^1^H NMR (400 MHz, CDCl_3_) δ 8.00 (d, *J* = 8.8 Hz, 2H), 7.77 (d, *J* = 15.7 Hz, 1H), 7.71 (d, *J* = 8.1 Hz, 2H), 7.65–7.60 (m, 3H), 6.91 (d, *J* = 8.9 Hz, 2H), 3.40 (t, *J* = 5.0 Hz, 4H), 2.55 (t, *J* = 5.0 Hz, 4H), 2.35 (s, 3H); ^13^C NMR (100 MHz, CDCl_3_) δ 187.36, 154.25, 140.98, 138.79, 131.39 (q, *J* = 32 Hz), 130.80, 128.34, 127.73, 125.80 (q, *J* = 3.7 Hz), 124.29, 123.94 (d, *J* = 271 Hz), 113.45, 54.70, 47.13, 46.13; HRMS (ESI-TOF) m/z calcd for C_21_H_22_F_3_N_2_O [M+H]^+^: 375.1684, found: 375.1693.

3. (*E*)-3-(4-fluorophenyl)-1-(4-(4-methylpiperazin-1-yl)phenyl)prop-2-en-1-one (**2c**)

Yield 74%; mp = 157–158 °C, IR (KBr, cm^−1^): 2795, 1648, 1605, 1593, 1413, 1220, ^1^H NMR (400 MHz, CDCl_3_) δ 7.99 (d, *J* = 9.0 Hz, 2H), 7.75 (d, *J* = 15.6 Hz, 1H), 7.62 (dd, *J* = 8.6, 5.4 Hz, 2H), 7.48 (d, *J* = 15.6 Hz, 1H), 7.09 (t, *J* = 8.6 Hz, 2H), 6.91 (d, *J* = 9.0 Hz, 2H), 3.40 (t, *J* = 5.1 Hz, 4H), 2.56 (t, *J* = 5.1 Hz, 4H), 2.35 (s, 3H); ^13^C NMR (100 MHz, CDCl_3_) δ 187.78, 163.81 (d, *J* = 249 Hz), 154.12, 141.79, 131.61 (d, *J* = 3.6 Hz), 130.67, 130.12 (d, *J* = 8.3 Hz), 128.13, 121.75 (d, *J* = 2.1 Hz), 116.00 (d, *J* = 22 Hz), 113.52, 54.75, 47.24, 46.16; HRMS (ESI-TOF) m/z calcd for C_20_H_22_FN_2_O [M+H]^+^: 325.1716, found: 325.1722.

4. (*E*)-4-(3-(4-(4-methylpiperazin-1-yl)phenyl)-3-oxoprop-1-en-1-yl)benzonitrile (**2d**)

Yield 84%; mp = 218–220 °C, IR (KBr, cm^−1^): 2801, 2224, 1650, 1603, 1582, ^1^H NMR (400 MHz, CDCl_3_) δ 7.99 (d, *J* = 9.0 Hz, 2H), 7.74 (d, *J* = 15.6 Hz, 1H), 7.70–7.67 (m, 4H), 7.63 (d, *J* = 15.6 Hz, 1H), 6.92 (d, *J* = 9.0 Hz, 2H), 3.42 (t, *J* = 5.0 Hz, 4H), 2.56 (t, *J* = 5.0 Hz, 4H), 2.36 (s, 3H); 13C NMR (100 MHz, CDCl_3_) δ 187.06, 154.32, 140.39, 139.76, 132.61, 130.85, 128.55, 127.54, 125.21, 118.54, 113.43, 112.99, 54.71, 47.11, 46.15; HRMS (ESI-TOF) m/z calcd for C_21_H_22_N_3_O [M+H]^+^: 332.1763, found: 332.1774.

5. (*E*)-3-(4-isopropylphenyl)-1-(4-(4-methylpiperazin-1-yl)phenyl)prop-2-en-1-one (**2e**)

Yield 78.3%; mp = 135–136 °C, IR (KBr, cm^−1^): 2842, 1649, 1601, 1583, ^1^H NMR (400 MHz, CDCl_3_) δ 7.99 (d, *J* = 8.9 Hz, 2H), 7.78 (d, *J* = 15.6 Hz, 1H), 7.57 (d, *J* = 8.2 Hz, 2H), 7.52 (d, *J* = 15.6 Hz, 1H), 7.27 (d, *J* = 8.1 Hz, 2H), 6.92 (d, *J* = 9.0 Hz, 2H), 3.39 (t, *J* = 5.1 Hz, 4H), 2.94 (quintet, *J* = 6.9 Hz, 1H), 2.56 (t, *J* = 5.1 Hz, 4H), 2.35 (s, 3H), 1.27 (d, *J* = 6.9 Hz, 6H); 13C NMR (100 MHz, CDCl_3_) δ 188.22, 154.04, 151.45, 143.25, 133.00, 130.64, 128.43, 127.02, 121.16, 113.57, 54.77, 47.30, 46.16, 34.12, 23.82; HRMS (ESI-TOF) m/z calcd for C_23_H_29_N_2_O [M+H]^+^: 349.228, found: 349.2291.

6. (*E*)-3-(4-(dimethylamino)phenyl)-1-(4-(4-methylpiperazin-1-yl)phenyl)prop-2-en-1-one (**2f**)

Yield 59.4%; mp = 182–184 °C, IR (KBr, cm^−1^): 2795, 1640, 1595, 1548, 1433, 1340, 1258, ^1^H NMR (400 MHz, CDCl_3_) δ 7.99 (d, *J* = 8.8 Hz, 2H), 7.78 (d, *J* = 15.4 Hz, 1H), 7.53 (d, *J* = 8.7 Hz, 2H), 7.37 (d, *J* = 15.4 Hz, 1H), 6.90 (d, *J* = 8.9 Hz, 2H), 6.67 (d, *J* = 8.8 Hz, 2H), 3.35 (t, *J* = 4.7 Hz, 4H), 3.00 (s, 6H), 2.54 (t, *J* = 4.8 Hz, 4H), 2.33 (s, 3H); 13C NMR (100 MHz, CDCl_3_) δ 188.27, 153.78, 151.77, 144.10, 130.37, 130.14, 129.12, 123.09, 116.85, 113.63, 111.87, 54.79, 47.42, 46.17, 40.16; HRMS (ESI-TOF) m/z calcd for C_22_H_28_N_3_O [M+H]^+^: 350.2232, found: 350.2239.

7. (*E*)-1-(4-(4-methylpiperazin-1-yl)phenyl)-3-(4-(methylthio)phenyl)prop-2-en-1-one (**2g**)

Yield 81%; mp = 166–167 °C, IR (KBr, cm^−1^): 2842, 1645, 1605, 1585, ^1^H NMR (400 MHz, CDCl_3_) δ 7.99 (d, *J* = 8.7 Hz, 2H), 7.74 (d, *J* = 15.6 Hz, 1H), 7.54 (d, *J* = 7.8 Hz, 2H), 7.52 (d, *J* = 14.9 Hz, 1H), 7.23 (d, *J* = 8.2 Hz, 2H), 6.90 (d, *J* = 8.8 Hz, 2H), 3.38 (t, *J* = 4.7 Hz, 4H), 2.54 (t, *J* = 4.7 Hz, 4H), 2.49 (s, 3H), 2.34 (s, 3H); 13C NMR (100 MHz, CDCl_3_) δ 187.88, 154.04, 142.54, 141.65, 131.88, 130.62, 128.67, 128.30, 126.03, 121.00, 113.52, 54.75, 47.25, 46.17, 15.22; HRMS (ESI-TOF) m/z calcd for C_21_H_25_N_2_OS [M+H]^+^: 353.1687, found: 353.1694.

8. (*E*)-3-(4-(difluoromethoxy)phenyl)-1-(4-(4-methylpiperazin-1-yl)phenyl)prop-2-en-1-one (**2h**)

Yield 92%; mp = 151–152 °C, IR (KBr, cm^−1^): 2798, 1650, 1605, 1605, 1589, 1224, ^1^H NMR (400 MHz, CDCl_3_) δ 7.99 (d, *J* = 8.8 Hz, 2H), 7.74 (d, *J* = 15.6 Hz, 1H), 7.63 (d, *J* = 8.6 Hz, 2H), 7.50 (d, *J* = 15.6 Hz, 1H), 7.14 (d, *J* = 8.4 Hz, 2H), 6.91 (d, *J* = 8.9 Hz, 2H), 6.56 (t, *J* = 73.5 Hz, 1H), 3.39 (t, *J* = 4.9 Hz, 4H), 2.55 (t, *J* = 4.9 Hz, 4H), 2.35 (s, 4H); 13C NMR (100 MHz, CDCl_3_) δ 187.68, 154.14, 152.34, 141.59, 132.56, 130.68, 129.81, 128.03, 122.10, 119.59, 115.65 (t, J = 259 Hz), 113.49, 54.73, 47.20, 46.14; HRMS (ESI-TOF) m/z calcd for C_21_H_23_F_2_N_2_O_2_ [M+H]^+^: 373.1727, found: 373.1732.

9. (*E*)-3-(3-fluoro-5-(trifluoromethyl)phenyl)-1-(4-(4-methylpiperazin-1-yl)phenyl)prop-2-en-1-one (**2i**)

Yield 79%; mp = 167–168 °C, IR (KBr, cm^−1^): 2800, 1654, 1608, 1250, ^1^H NMR (400 MHz, CDCl_3_) δ 8.00 (d, *J* = 9.0 Hz, 2H), 7.72 (d, *J* = 15.6 Hz, 1H), 7.66 (s, 1H), 7.59 (d, *J* = 15.6 Hz, 1H), 7.49 (d, *J* = 9.1 Hz, 1H), 7.34 (d, *J* = 8.1 Hz, 1H), 6.92 (d, *J* = 9.0 Hz, 2H), 3.42 (t, *J* = 5.1 Hz, 4H), 2.57 (t, *J* = 5.1 Hz, 4H), 2.36 (s, 3H); 13C NMR (100 MHz, CDCl_3_) δ 186.95, 162.75 (d, *J* = 247 Hz), 154.34. 139.70, 138.82 (d, *J* = 8.0 Hz), 130.87, 127.50, 124.93, 120.62, 117.99, 117.77, 113.90, 113.69, 113.45, 54.73, 47.14, 46.15; HRMS (ESI-TOF) m/z calcd for C_21_H_21_F_4_N_2_O [M+H]^+^: 393.159, found: 393.1592.

10. (*E/Z*)-3-(3,5-bis(trifluoromethyl)phenyl)-1-(4-(4-methylpiperazin-1-yl)phenyl)prop-2-en-1-one (2**j**)

Yield 78% (E/Z, 4:1); mp = 160–162 °C, IR (KBr, cm^−1^): 2801, 1655, 1583, 1253, ^1^H NMR (400 MHz, CDCl_3_) δ 8.02 (d, *J* = 12 Hz, 4H), 7.87–7.81 (m, 1H) 7.79 (d, *J* = 15.6 Hz, 1H), 7.66 (d, *J* = 15.6 Hz, 1H), 6.93 (d, *J* = 9.0 Hz, 2H), 3.43 (t, *J* = 5.0 Hz, 4H), 2.57 (t, *J* = 5.0 Hz, 4H), 2.36 (s, 3H); 13C NMR (100 MHz, CDCl_3_) δ 186.72, 154.39, 139.18, 137.57, 132.39 (d, *J* = 33 Hz), 130.93, 127.77, 127.35, 125.49, 123.12 (d, *J* = 271 Hz), 122.98, 113.44, 54.71, 47.10, 46.14; HRMS (ESI-TOF) m/z calcd for C_22_H_21_F_6_N_2_O [M+H]^+^: 443.1558, found: 443.1561.

11. (*E*)-3-(4-fluoro-3-(trifluoromethyl)phenyl)-1-(4-(4-methylpiperazin-1-yl)phenyl)prop-2-en-1-one (**2k**)

Yield 82.3%; mp = 133–135 °C, IR (KBr, cm^−1^): 2805, 1652, 1605, 1466, 1225, ^1^H NMR (400 MHz, CDCl_3_) δ 7.99 (d, *J* = 8.8 Hz, 2H), 7.86 (d, *J* = 4.4 Hz, 1H), 7.80–7.77 (m, 1H), 7.73 (d, *J* = 15.6 Hz, 1H), 7.53 (d, *J* = 15.6 Hz, 1H), 7.23 (d, *J* = 9.8 Hz, 1H), 6.92 (d, *J* = 8.8 Hz, 2H), 3.41 (t, *J* = 5.0 Hz, 4H), 2.56 (t, *J* = 5.0 Hz, 4H), 2.36 (s, 3H); 13C NMR (100 MHz, CDCl_3_) δ 187.15, 154.27, 141.44, 139.97, 133.60, 133.52, 131.99, 130.79, 130.66, 127.67, 126.61, 123.45, 121.04, 117.72, 117.62 (d, *J* = 21 Hz), 113.50 (d, *J* = 5.0 Hz), 54.73, 47.16, 46.15; HRMS (ESI-TOF) m/z calcd for C_21_H_21_F_4_N_2_O [M+H]^+^: 393.1590, found: 393.1595.

12. (*E*)-3-(4-chloro-3-(trifluoromethyl)phenyl)-1-(4-(4-methylpiperazin-1-yl)phenyl)prop-2-en-1-one (**2l**)

Yield 77.4%; mp = 139–140 °C, IR (KBr, cm^−1^): 2807, 1650, 1605, 1583, 1365, 1253, ^1^H NMR (400 MHz, CDCl_3_) δ 7.99 (d, *J* = 8.9 Hz, 2H), 7.92 (d, *J* = 1.1 Hz, 1H), 7.72 (d, *J* = 15.6 Hz, 1H), 7.69 (s, 1H), 7.58 (d, *J* = 15.6 Hz, 1H), 7.54 (d, *J* = 8.4 Hz, 1H), 6.92 (d, *J* = 9.0 Hz, 2H), 3.41 (t, *J* = 5.0 Hz, 4H), 2.56 (t, *J* = 5.0 Hz, 4H), 2.36 (s, 3H); 13C NMR (100 MHz, CDCl_3_) δ 187.08, 154.29, 139.89, 134.40, 133.40, 132.09, 132.05, 130.82, 129.03 (d, *J* = 31 Hz), 127.61, 126.82 (d, *J* = 5.2 Hz), 124.05, 122.61 (d, *J* = 272 Hz), 113.45, 54.73, 47.15, 46.15; HRMS (ESI-TOF) m/z calcd for C_21_H_21_ClF_3_N_2_O [M+H]^+^: 409.1294, found: 409.1303.

13. (*E*)-3-(4-fluoro-3-methoxyphenyl)-1-(4-(4-methylpiperazin-1-yl)phenyl)prop-2-en-1-one (**2m**)

Yield 84%; mp = 153–154 °C, IR (KBr, cm^−1^): 2794, 1646, 1602, 1578, 1235, ^1^H NMR (400 MHz, CDCl_3_) δ 7.97 (d, *J* = 8.8 Hz, 2H), 7.69 (d, *J* = 15.6 Hz, 1H), 7.44 (d, *J* = 15.6 Hz, 1H), 7.20–7.16 (m, 2H), 7.10–7.05 (m, 1H), 6.90 (d, *J* = 8.8 Hz, 2H), 3.93 (s, 3H), 3.38 (t, *J* = 4.9 Hz, 4H), 2.54 (t, *J* = 4.8 Hz, 4H), 2.34 (s, 3H); 13C NMR (100 MHz, CDCl_3_) δ 187.89, 154.13, 153.63 (d, *J* = 250 Hz), 147.97 (d, *J* = 11 Hz), 142.17, 132.04 (d, *J* = 3.8 Hz), 130.67, 128.12, 121.92, 121.49 (d, *J* = 7.0 Hz), 116.46 (d, *J* = 19 Hz), 113.51, 112.92, 56.34, 54.74, 47.24, 46.13; HRMS (ESI-TOF) m/z calcd for C_21_H_24_FN_2_O_2_ [M+H]^+^: 355.1822, found: 355.1826.

14. (*E*)-3-(5-bromo-2-fluorophenyl)-1-(4-(4-methylpiperazin-1-yl)phenyl)prop-2-en-1-one (**2n**)

Yield 64%; mp = 133–135 °C, IR (KBr, cm^−1^): 2803, 1651, 1590, 1303, ^1^H NMR (400 MHz, CDCl_3_) δ 7.99 (d, *J* = 9.0 Hz, 2H), 7.78 (d, *J* = 15.7 Hz, 1H), 7.74 (d, *J* = 2.4 Hz, 1H), 7.63 (d, *J* = 15.8 Hz, 1H), 7.43 (ddd, *J* = 8.7, 4.5, 2.5 Hz, 1H), 7.00 (dd, *J* = 10, 8.8 Hz, 1H), 6.90 (d, *J* = 9.0 Hz, 2H), 3.40 (t, *J* = 5.1 Hz, 4H), 2.55 (t, *J* = 5.1 Hz, 4H), 2.35 (s, 3H); 13C NMR (100 MHz, CDCl_3_) δ 187.26, 160.50 (d, *J* = 252 Hz), 154.22, 133.90, 133.79, 131.75 (d, *J* = 3.0 Hz), 130.84, 127.68, 125.63 (d, *J* = 6.2 Hz), 125.49 (d, *J* = 13 Hz), 117.99 (d, *J* = 24 Hz), 116.98 (d, *J* = 3.0 Hz), 113.44, 54.71, 47.13, 46.15; HRMS (ESI-TOF) m/z calcd for C_20_H_21_BrFN_2_O [M+H]^+^: 403.0821, found: 403.0836.

15. (*E*)-3-(2-chloro-5-(trifluoromethyl)phenyl)-1-(4-(4-methylpiperazin-1-yl)phenyl)prop-2-en-1-one (**2o**)

Yield 85.4%; mp = 128–129 °C, IR (KBr, cm^−1^): 2800, 1651, 1591, 1255, ^1^H NMR (400 MHz, CDCl_3_) δ 8.11 (dd, *J* = 15.7, 1.8 Hz, 1H), 8.00 (d, *J* = 7.3 Hz, 2H), 7.98 (d, *J* = 11.5 Hz, 1H), 7.58–7.54 (m, 3H), 6.92 (dd, *J* = 9.0, 2.2 Hz, 2H), 3.41 (s, 4H), 2.55 (s, 4H), 2.35 (s, 3H); 13C NMR (100 MHz, CDCl_3_) δ 187.05, 154.29, 138.64, 137.17, 134.63, 130.93, 130.82, 129.60 (d, *J* = 33 Hz), 127.47, 126.90, 126.30, 124.52 (d, *J* = 3.6 Hz), 123.57 (d, *J* = 270 Hz), 113.43, 54.70, 47.09, 46.13; HRMS (ESI-TOF) m/z calcd for C_21_H_21_ClF_3_N_2_O [M+H]^+^: 409.1294, found: 409.1295.

16. (*E*)-3-(4-methoxy-2-(trifluoromethyl)phenyl)-1-(4-(4-methylpiperazin-1-yl)phenyl)prop-2-en-1-one (**2p**)

Yield 91%; mp = 133–134 °C, IR (KBr, cm^−1^): 2801, 1650, 1592, 1320, ^1^H NMR (400 MHz, CDCl_3_) δ 8.06 (dd, *J* = 15.4, 2.0 Hz, 1H), 7.97 (d, *J* = 9.0 Hz, 2H), 7.79 (d, *J* = 8.7 Hz, 1H), 7.39 (d, *J* = 15.4 Hz, 1H), 7.20 (d, *J* = 2.6 Hz, 1H), 7.07 (dd, J = 8.7, 2.5 Hz, 1H), 6.89 (d, *J* = 9.0 Hz, 2H), 3.86 (s, 3H), 3.38 (t, *J* = 5.1 Hz, 4H), 2.54 (t, *J* = 5.0 Hz, 4H), 2.34 (s, 3H); 13C NMR (100 MHz, CDCl_3_) δ 187.69, 160.37, 154.11, 138.03, 130.75, 130.48 (d, *J* = 30 Hz), 129.48, 127.88, 126.40, 124.26, 123.77 (d, *J* = 273 Hz), 117.23, 113.46, 112.04 (q, *J* = 5.8 Hz), 55.65, 54.72, 47.18, 46.13; HRMS (ESI-TOF) m/z calcd for C_22_H_24_F_3_N_2_O_2_ [M+H]^+^: 405.1790, found: 405.1800.

17. (*E*)-3-(2,4-difluorophenyl)-1-(4-(4-methylpiperazin-1-yl)phenyl)prop-2-en-1-one (**2q**)

Yield 85%; mp = 138–141 °C, IR (KBr, cm^−1^): 2802, 1651, 1588, 1427, 1292, ^1^H NMR (400 MHz, CDCl_3_) δ 7.98 (d, *J* = 8.9 Hz, 2H), 7.81 (d, *J* = 15.8 Hz, 1H), 7.65–7.52 (m, 2H), 6.94–6.62 (m, 4H), 3.40 (t, *J* = 5.0 Hz, 4H), 2.55 (t, *J* = 5.0 Hz, 4H), 2.35 (s, 3H); 13C NMR (100 MHz, CDCl_3_) δ 187.71, 154.09 (d, *J* = 15 Hz), 136.07, 134.76, 130.83 (d, *J* = 4.8 Hz), 130.75, 129.27 (d, *J* = 269 Hz), 124.22 (d, *J* = 7.1 Hz), 119.90, 113.54, 113.49, 112.10, 111.85, 104.92, 104.66, 104.40, 64.09, 54.73, 47.29, 47.19, 46.15; HRMS (ESI-TOF) m/z calcd for C_20_H_21_F_2_N_2_O [M+H]^+^: 343.1622, found: 343.1622.

### 3.3. Monoamine Oxidase (MAO) and Cholinesterase (ChE) Biological Studies

Materials: Monoamine oxidase A and B (MAO-A and MAO-B), acetyl and butyrylcholinesterase (AChE and BChE), kynuramine, benzylamine, acetylthiocholine iodide (ATCI), S-butyrylthiocholine iodide (BTCI), 5,5′-dithiobis(2-nitrobenzoic acid) (DTNB), toloxatone, clorgyline, lazabemide, pargyline, donepezil, and dimethyl sulfoxide (DMSO) were obtained from Sigma Aldrich (St. Louis, MO, USA).

#### 3.3.1. Enzyme Inhibition Assays

MAO assays were performed as described previously [45], i.e., ~0.09 U/mL of MAO-A and MAO-B were reacted with 0.06 mM kynuramine and 0.3 mM benzylamine, respectively, in 50 mM sodium phosphate (pH 7.2). The absorbances of reaction mixtures of MAO-A and MAO-B were observed at 316 nm and 250 nm, respectively, for 30 min under kinetic mode of the spectrophotometer (OPTIZEN, K-Lab, Dajeon, Republic of Korea). For inhibition assay, the compounds were dissolved in DMSO and added into the reaction mixture. The final concentration of DMSO in the reaction mixture was less than 1% to avoid solvent disturbance.

For measuring cholinesterase (ChE) activity, the Ellman method [46] was employed with a slight modification. The AChE and BchE with ~0.02 U/mL were reacted with 0.5 mM ATCI and BTCI, respectively, followed by addition of 0.5 mM DTNB. The absorbance of the reaction mixture was continuously observed at 412 nm for 15 min under the kinetic mode of the spectrophotometer. In the inhibition assay, the enzyme and the compound were preincubated in reaction buffer for 15 min, followed by adding substrates and DTNB.

#### 3.3.2. Kinetics and Reversibility Analysis

To analyze the inhibitory patterns of compounds **2k** and **2n**, which showed the most potent MAO-B inhibition with the lowest IC_50_, kinetics and reversibility tests were conducted [47]. In kinetics for MAO-B, 0.038, 0.075, 0.15, 0.3, and 0.6 mM of benzylamine were used to construct Lineweaver–Burk plots, and the compounds were added with concentrations of ~1/2× IC_50_, IC_50_, and 2× IC_50_ for a secondary plot to obtain K_m_ and K_i_ values. In the kinetics for AChE, 0.0625, 0.125, 0.25, 0.5, 1.0 mM of ATCI were used in the presence of DTNB, and the compounds were added to be ~1/2× IC_50_, IC_50_, and 2× IC_50_ concentrations for the secondary plot to obtain K_m_ and K_i_ values.

Reversibility of the compounds for MAO-B was measured using the dialysis method. The compounds were treated with ~1.5× IC_50_ for **2k** and ~2× IC_50_ for **2n**, respectively, with the reaction mixture without substrate. The reaction mixture was placed in a dialysis tube (DiaEasyTM Dialyzer (800 µL) MWCO 6–8 kDa, Biovision, CA, USA) and dialyzed for 6 h. After that, the 0.3 mM of benzylamine was added into the reaction mixture for measuring enzyme activity. The relative activity to the control (without inhibitors) before and after dialysis was calculated, respectively. The lazabemide and pargyline were used as standard reversible and irreversible MAO-B inhibitors, respectively.

### 3.4. Cell Cytotoxicity Study

#### 3.4.1. Materials

The reference compound staurosporine was purchased from Sigma-Aldrich (Saint Louis, MO, USA). Compounds **2k** and **2n** were dissolved in DMSO as 50 mM stock. CellTiter 96^®^ Non-Radioactive Cell Proliferation Assay (MTT) reagent was purchased from Promega (Cat# G4000). The normal Vero cell line was purchased from ATCC. Normal Vero cells were cultured with EMEM medium plus 10% FBS. In total, 100 µg/mL penicillin and 100 µg/mL streptomycin were added to the culture media. Cultures were maintained at 37 °C in a humidified atmosphere of 5% CO_2_ and 95% air.

#### 3.4.2. Method

Compounds **2k** and **2n** and staurosporine were diluted with DMSO in 10-dose and 3-fold serial dilution starting at 50 mM (test compounds) and 10 mM (staurosporine). The test compounds (100 nL) and staurosporine (25 nL) were delivered from the source plate to the wells of 384-well cell culture plates by Echo 550 Liquid Handler. Culture medium (25 µL) containing 2000 of normal Vero cells was added to the 384-well cell culture plates, and the plates were incubated at 37 °C in a humidified atmosphere of 5% CO_2_ and 95% air for 72 h. MTT Dye Solution (8 µL) was added to each well, and the plates were incubated at 37 °C in a CO_2_ incubator for 6 h. After incubation, the formazan was produced in the cells. Solubilization/Stop Solution (30 µL) was added to each well and the plates were incubated for another 1 h at 37 °C in a CO_2_ incubator to dissolve the formazan crystals. The absorbance of each sample was measured at 590 nm using Envision 2104 Multilabel Reader (PerkinElmer, Santa Clara, CA, USA), and the cell viability was determined based on the quantification of the color intensity in each culture well (duplicate assay mode). The IC_50_ curves were plotted, and the IC_50_ values were calculated using the GraphPad Prism 4 program based on a sigmoidal dose–response equation.

### 3.5. Molecular Docking

Docking simulation of three enzymes, MAO-A, MAO-B, and AChE with compounds **2k** and **2n** were performed using AUTODOCK-VINA [40]. The protein structures of MAO-A, MAO-B, and AChE are PDB (Protein Data Bank) ID of 2Z5X, 4A79, and 6O4W, respectively. MAO-B and AChE have a homogenous complex with two chains, A and B. We used two programs; the first program predicts a binding site, and the second program, Pck, is a pocket search program, not a binding site. Because the pocket and the binding site are not identical, we cannot find another binding site working under allosteric inhibition when the docking simulation runs only in the binding site. The first program is for understanding the enzyme, and the second one is for general docking procedure. For the docking simulation, the first chain, A, was chosen. The binding sites of two proteins were determined using a binding pocket program, DPSP (dockable pocket site prediction: as an in-house program). It searches and collects all of the ligands existing on similar sequence structures, and obtains pockets available on the MAO and AChE proteins. Compounds **2k** and **2n** were sketched, energy minimized, and converted to the general structure format, i.e., PDB, using the Marvin program (ChemAxon; http://www.chemaxon.com, accessed on 1 July 2022). For the docking simulation, the pockets in these proteins were searched utilizing Pck pocket detection program (http://schwarz.benjamin.free.fr/Work/Pck/home.htm, accessed on 1 July 2022), where it detects the amino acids forming pockets and measures their volumes. Twelve, thirteen, and fourteen pockets over 100 Å^3^ of volume were found in MAO-A, MAO-B, and AChE, respectively. These pockets consist of 168, 158, and 101 amino acids for MAO-A, MAO-B, and AChE. Compounds **2k** and **2n** were put on Carbon α atom positions in these pocket residues, and the docking simulations were run ten times with different random seeds, i.e., a total of 1680, 1580, and 1010 simulations were carried out for MAO-A, MAO-B, and AChE, respectively. The box size with a length of 15 Å was used to prevent the compounds from drifting from the center of the pocket residue. With these docking poses, the clustering based on the center of mass was performed to group these docking conformations using CHARMM (Chemistry at HARvard Macromolecular Mechanics) [48]. The compounds were ranked by the lowest energy of the group, the largest number of the group, and the lowest energy conformation in the group. The molecular structures were drawn by VMD (visual molecular dynamics) visualizer program (https://www.ks.uiuc.edu/Research/vmd/, accessed on 1 July 2022). Hydrogen bond and π-π interaction analysis between each compound and proteins were calculated based on a geometric analysis. First, the acceptor and donor atoms of hydrogen bonds were assigned and then their Cartesian coordinates were obtained. Second, all of the pairs between acceptor and donor atoms were arranged to measure geometric distance and angle. The used hydrogen bonding criteria is the distance below 4.5 Å between the acceptor and donor atoms. The π-π interaction forms between two close parallel aromatic rings. First, the aromatic rings were sought, and then the atoms forming the aromatic rings were found. The centroid of these atoms was calculated and the Cartesian coordinate was obtained. The distance between the two centroids was measured, in the case that both aromatic rings are close and parallel.

### 3.6. Pharmacokinetic Prediction of the ***2k*** and ***2n*** Using the SwissADME Web Tool

The pharmacokinetic and physicochemical properties such as gastrointestinal (GI) absorption, BBB permeability, P-glycoprotein (P-gp) substrate, cytochrome P450 (CYP), and Lipinski parameters [43] of **2k** and **2n** were predicted using the web tool of SwissADME (http://www.swissadme.ch, accessed on 1 July 2022) [49].

## 4. Conclusions

In this report, we describe the synthesis of new *N*-methyl-piperazine chalcones, and their MAOs and AChE inhibition profiles. Most of the tested compounds exerted potent MAO-B inhibition with considerable SI along with moderate AChE inhibition. The fluorinated substituted derivatives **2k** and **2n** inhibited MAO-B with K_i_ values of sub-micromolar range. The kinetics study of **2k** and **2n** disclosed their competitive reversible MAO-B inhibitory pattern. A cell-based assay of **2k** and **2n** against normal Vero cells revealed their minimized cytotoxicity. A docking simulation predicted that **2k** showed the lowest energy for MAO-B (−11.6 kcal/mol) with four hydrogen bonds and two π-π interactions. In silico ADME/Tox prediction studies of **2k** and **2n** pointed out their favorable drug-like properties. Overall, compounds **2k** and **2n** could serve as promising MAO-B/AChE inhibitors for the treatment of NDD.

## Figures and Tables

**Table 1:** Preliminary assay of compounds **2a**–**q** against MAO-A, MAO-B, AChE, and BChE at 10 µM.

ID	Residual Activities at 10 μM (%)			
	MAO-A	MAO-B	AChE	BChE
**2a**	117.97 ± 7.73	12.78 ± 0.79	34.94 ± 5.19	58.65 ± 6.80
**2b**	88.19 ± 6.87	21.02 ± 4.02	18.99 ± 0.55	58.50 ± 1.38
**2c**	103.47 ± 8.84	22.73 ± 1.61	49.42 ± 4.66	49.67 ± 3.70
**2d**	72.35 ± 2.50	16.67 ± 4.71	24.26 ± 0.95	71.53 ± 5.25
**2e**	60.56 ± 3.98	44.09 ± 3.04	17.75 ± 1.85	49.32 ± 1.28
**2f**	83.58 ± 4.22	21.33 ± 3.77	26.37 ± 1.85	76.51 ± 3.35
**2g**	97.18 ± 1.99	40.00 ± 1.67	50.39 ± 4.43	74.66 ± 4.48
**2h**	55.22 ± 4.12	17.33 ± 3.77	37.99 ± 0.92	73.92 ± 3.35
**2i**	114.29 ± 6.06	20.35 ± 2.47	51.49 ± 4.92	63.72 ± 6.24
**2j**	97.14 ± 4.04	67.78 ± 5.36	57.12 ± 5.39	86.03 ± 1.04
**2k**	96.43 ± 3.03	−8.72 ± 0.82	46.85 ± 2.58	61.76 ± 5.54
**2l**	85.88 ± 1.66	0.00 ± 1.79	68.50 ± 1.32	73.02 ± 1.75
**2m**	78.82 ± 1.66	39.87 ± 6.26	27.61 ± 1.90	66.83 ± 2.10
**2n**	55.63 ± 1.00	6.45 ± 3.04	30.55 ± 1.11	43.21 ± 2.88
**2o**	92.54 ± 4.23	22.87 ± 2.26	37.60 ± 0.00	6.46 ± 3.05
**2p**	44.53 ± 3.31	15.00 ± 3.93	37.84 ± 7.64	28.85 ± 4.08
**2q**	72.66 ± 3.31	13.11 ± 2.06	33.78 ± 0.27	55.29 ± 0.68

Results are expressed as the means ± SD of at least two experiments.

**Table 2:** Inhibitory activities (IC_50_, μM) and selectivity indices of compounds **2a–q** against MAO-A, MAO-B, AChE, and BChE.

Compound No.	IC_50_ (μM)				SI (MAO)	SI (ChE)
	MAO-A	MAO-B	AChE	BChE		
**2a**	>40	2.58 ± 0.19	5.70 ± 0.22	13.89 ± 2.22	15.50	2.44
**2b**	>40	3.10 ± 0.04	2.26 ± 0.29	13.37 ± 0.27	12.90	5.92
**2c**	>40	2.98 ± 0.18	10.30 ± 1.03	18.41 ± 3.17	13.42	1.79
**2d**	>40	2.20 ± 0.06	2.38 ± 0.13	24.71 ± 2.00	18.18	10.38
**2e**	16.21 ± 1.74	8.36 ± 0.39	3.75 ± 0.82	14.36 ± 0.40	1.94	3.83
**2f**	>40	3.29 ± 0.08	3.03 ± 0.31	>40	12.16	>13.20
**2g**	>40	6.74 ± 0.03	12.41 ± 1.96	>40	5.93	>3.22
**2h**	>40	6.14 ± 0.43	5.82 ± 0.51	>40	6.51	>6.87
**2i**	>40	2.33 ± 0.14	13.93 ± 0.08	25.30 ± 2.96	17.17	1.82
**2j**	>40	15.70 ± 1.17	15.36 ± 2.43	>40	2.55	>2.60
**2k**	>40	0.71 ± 0.03	8.10 ± 0.69	21.09 ± 0.23	56.34	2.60
**2l**	>40	1.41 ± 0.04	14.07 ± 0.54	26.09 ± 1.28	28.37	1.85
**2m**	>40	6.76 ± 0.82	4.45 ± 0.24	18.61 ± 1.35	5.92	4.18
**2n**	17.80 ± 0.28	1.11 ± 0.06	4.32 ± 0.39	9.44 ± 0.05	16.04	2.19
**2o**	>40	3.20 ± 0.32	3.87 ± 0.18	1.19 ± 0.06	12.50	0.31
**2p**	6.67 ± 0.63	2.18 ± 0.01	7.17 ± 0.60	7.58 ± 0.25	3.06	1.06
**2q**	16.52 ± 1.75	3.03 ± 0.13	6.37 ± 0.17	10.89 ± 0.90	5.45	1.71
Toloxatone	1.08 ± 0.03	-	-	-	-	-
Lazabemide		0.11 ± 0.02	-	-	-	-
Clorgyline	0.007 ± 0.001	-	-	-	-	-
Pargyline	-	0.14 ± 0.01	-	-	-	-
Tacrine	-	-	0.27 ± 0.02	0.060 ± 0.002	-	-
Donepezil	-	-	0.010 ± 0.002	0.180 ± 0.004	-	-
PC5 *	34.1 ± 2.51	2.31 ± 0.46	-	-	14.8	
PC10 *	31.4 ± 3.50	0.65 ± 0.023	28.0 ± 2.43	36.4 ± 2.36		
PC11 *	34.9 ± 4.10	0.71 ± 0.0035	26.3 ± 1.29	36.2 ± 3.65		

Results are expressed as the means ± SD of at least two experiments. SI values: MAO, MAO-B selectivity over MAO-A; ChE, AChE selectivity over BChE, based on their respective IC_50_ values. *, from ref [33].

**Table 3:** Detailed docking results of compounds **2k** and **2n** on MAO-A, MAO-B, and AChE ^a^.

Enzyme	Cpds	No. of Cpds	Ave. Energy	Lowest Energy (Rank)	Free Energy	No. of HB	No. of π-π/Aro.
MAO-A	**2k**	32	−5.97 ± 2.56	−10.40 (2)	−12.48	4	1/5
	**2n**	50	−5.88 ± 2.42	−11.20 (2)	−13.55	2	1/4
MAO-B	**2k**	10	−5.47 ± 3.70	−11.60 (1)	−12.98	4	2/7
	**2n**	16	−5.33 ± 3.31	−10.70 (1)	−12.36	2	2/7
AChE	**2k**	41	−7.81 ± 1.15	−9.90 (1)	−12.13	7	1/9
	**2n**	39	−7.74 ± 1.25	−9.60 (1)	−11.80	4	1/9

^a^ Each first lowest energy compound in the catalytic region is chosen. The result is tabulated in terms of number of compounds existing on the pocket (No. of Cpds), average energy on the pocket, the number of docking compounds on the same pocket, lowest energy with cluster rank with Parentheses, free energy, number of Hydrogen bonding, and number of π-π interaction/aromatic rings. Energy unit is kcal/mol. The free energy is the colony energy [43] calculated by E–kT log(N), where E is the lowest energy, N is the number of compounds in the catalytic region. The k is Boltzmann constant and T is room temperature, 300 K. The free energy considers both energetic and entropic contribution.

**Table 4:** Predicted pharmacokinetic properties of **2k** and **2n**.

Compound No.	GI Absorption	BBB Permeant	P-gp Substrate	CYP1A2 Inhibitor	CYP2C19 Inhibitor	CYP2C9 Inhibitor	CYP2D6 Inhibitor	CYP3A4 Inhibitor	Log *K_p_* (Skin Permeation)
**2k**	High	Yes	No	No	Yes	No	Yes	Yes	−5.42 cm/s
**2n**	High	Yes	No	No	Yes	Yes	Yes	No	−5.62 cm/s

GI: gastrointestinal; BBB: blood–brain barrier; P-gp: P-glycoprotein; CYP: Cytochrome P.

**Table 5:** Physicochemical properties of **2k** and **2n**.

Compound No.	Mw	cLog P_o/w_	HBD	HBA	TPSA (Å^2^)	RB	Lipinski Violations *
**2k**	392.40	4.48	0	6	23.55	5	0
**2n**	403.30	4.03	0	3	23.55	4	0

Mw: molecular weight; cLog P: consensus Log P_o/w_; HBD: H-bond donors; HBA: H-bond acceptors; TPSA: topological polar surface area; RB: rotatable bonds. * Lipinski violation is indicated by counting the number of violations in the Lipinski’s rule, i.e., MW < 500, cLog P_o/w_ < 5.00, HBD < 5, HBA < 10, TPSA < 140 Å^2^, and RB < 10.

## Data Availability

Data are contained within the article and Supplementary Materials.

## Supplementary Materials

The following supporting information can be downloaded at: https://www.mdpi.com/article/10.3390/ph16010083/s1, this section includes; Figure S1, ^1^H NMR and ^13^C NMR spectra; Figure S2, HRMS charts; Figure S3, IR spectra; Figure S4, IC_50_ curves of **2k** and **2n** against MAO-B.
