# Supplementary material for: Identification of New N-methyl-piperazine Chalcones as Dual MAO-B/AChE Inhibitors
Source: Pharmaceuticals (Basel). 2023 Jan 6;16(1):83. doi: 10.3390/ph16010083 (PMC9860728; doi:10.3390/ph16010083)
Supplement: Supplementary file 1 [file pharmaceuticals-16-00083-s001.zip › pharmaceuticals-2086141-supplementary.pdf]

Supplementary Information

Identification of New *N*-methyl-piperazine Chalcones as Dual MAO-B/AChE Inhibitors

Ashraf K. El-Damasy [1,2,*,†], Jong Eun Park [3,†], Hyun Ji Kim [1], Jinhyuk Lee [4,5], Eun-Kyoung Bang [1], Hoon Kim [3,*] and Gyochang Keum [1,*]

[1] Center for Brain Technology, Brain Science Institute, Korea Institute of Science and Technology (KIST), Seoul 02792, Republic of Korea; hjkim926@kist.re.kr (H.J.K.); eunkbang@kist.re.kr (E.-K.B.)
[2] Department of Medicinal Chemistry, Faculty of Pharmacy, Mansoura University, Mansoura 35516, Egypt
[3] Department of Pharmacy, and Research Institute of Life Pharmaceutical Sciences, Sunchon National University, Suncheon 57922, Republic of Korea; park140201@naver.com
[4] Disease Target Structure Research Center, Korea Research Institute of Bioscience and Biotechnology (KRIBB), Daejeon 34141, Republic of Korea; jinhyuk@kribb.re.kr
[5] Department of Bioinformatics, KRIBB School of Bioscience, University of Science and Technology (UST), Daejeon 34113, Republic of Korea
* Correspondence: ashraf.el-damasy@kist.re.kr (A.K.E.-D.); hoon@sunchon.ac.kr (H.K.); gkeum@kist.re.kr (G.K.)
† These authors contributed equally to this work.

Contents **Pages**

Contents	Pages
Figure S1. ^{1}H NMR and ^{13}C NMR spectra	2–18
Figure S2. HRMS charts	19–35
Figure S3. IR spectra	36–44
Figure S4. IC_{50} curves of **2k** and **2n** against MAO-B	45

Figure S1. ^{1}**H NMR and** ^{13}**C NMR spectra**

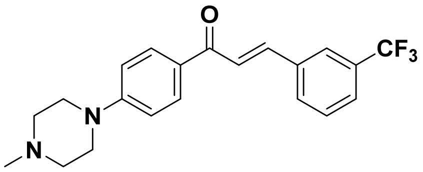

2a

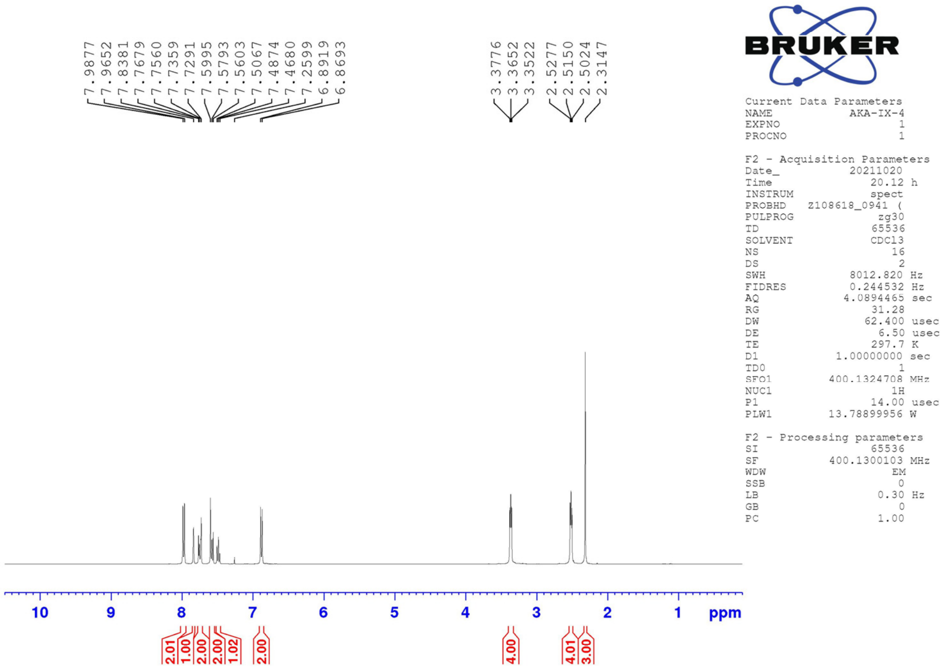

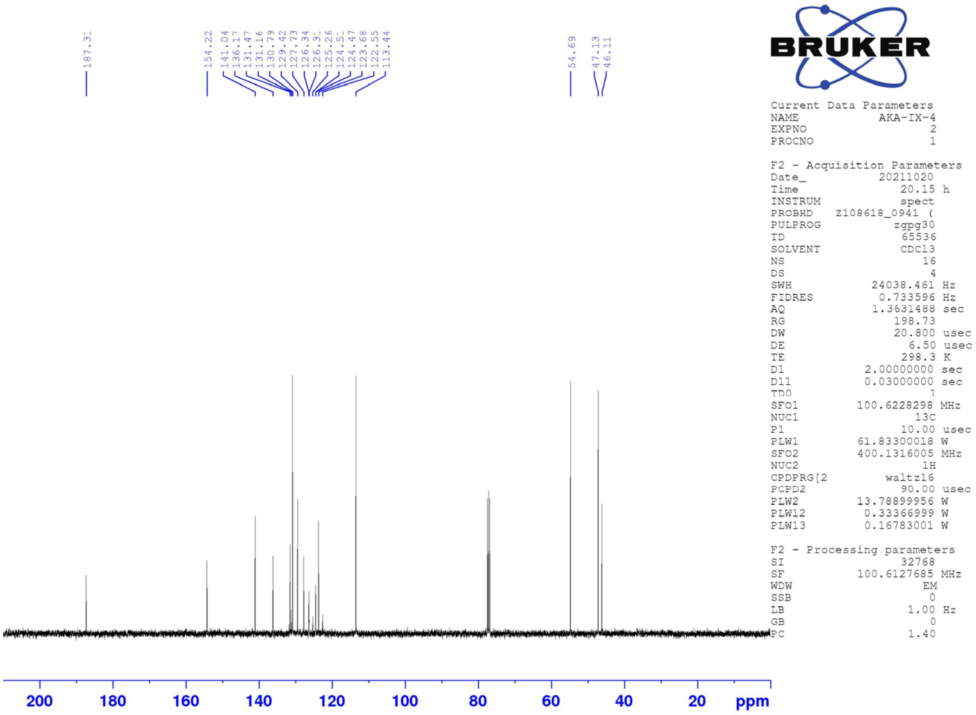

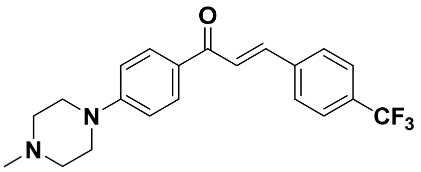
O
N
N
CF3

2b

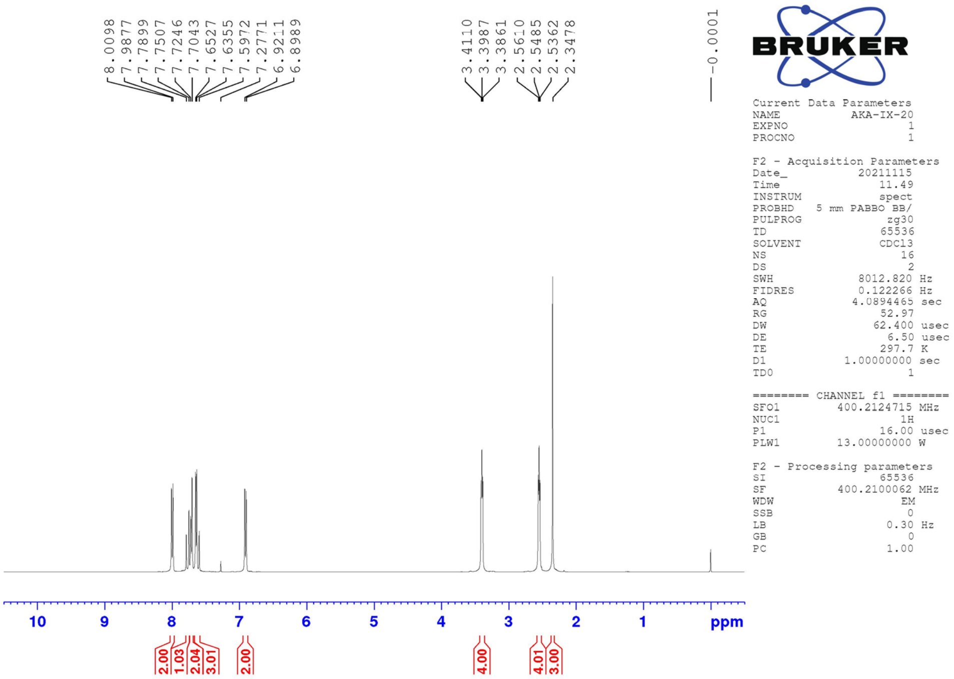
8.0098
7.9877
7.7899
7.7507
7.7246
7.7043
7.6527
7.6355
7.5972
7.2771
6.9211
6.8989
3.4110
3.3987
3.3861
2.5610
2.5485
2.5362
2.3478
-0.0001
BRUKER
Current Data Parameters
NAME AKA-IX-20
EXPNO 1
PROCNO 1
F2 - Acquisition Parameters
Date_ 20211115
Time 11.49
INSTRUM spect
PROBHD 5 mm PABBO BB/
PULPROG zg30
TD 65536
SOLVENT CDCl3
NS 16
DS 2
SWH 8012.820 Hz
FIDRES 0.122266 Hz
AQ 4.0894465 sec
RG 52.97
DW 62.400 usec
DE 6.50 usec
TE 297.7 K
D1 1.00000000 sec
TD0 1
======== CHANNEL f1 ========
SFO1 400.2124715 MHz
NUC1 1H
P1 16.00 usec
PLW1 13.00000000 W
F2 - Processing parameters
SI 65536
SF 400.2100062 MHz
WDW EM
SSB 0
LB 0.30 Hz
GB 0
PC 1.00
10 9 8 7 6 5 4 3 2 1 ppm
2.00
1.03
2.04
3.01
2.00
4.00
4.01
3.00

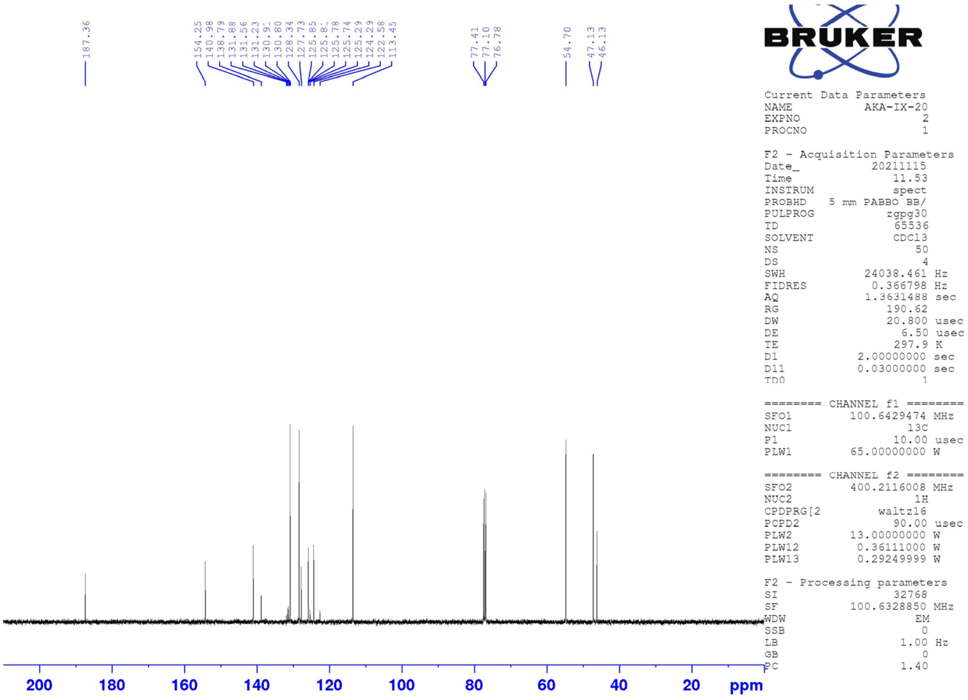
187.36
154.25
140.98
138.79
131.88
131.56
131.23
130.80
128.34
127.73
125.78
125.74
125.29
124.29
122.58
113.45
77.41
77.10
76.78
54.70
47.13
46.13
BRUKER
Current Data Parameters
NAME AKA-IX-20
EXPNO 2
PROCNO 1
F2 - Acquisition Parameters
Date_ 20211115
Time 11.53
INSTRUM spect
PROBHD 5 mm PABBO BB/
PULPROG zgpg30
TD 65536
SOLVENT CDCl3
NS 50
DS 4
SWH 24038.461 Hz
FIDRES 0.366798 Hz
AQ 1.3631488 sec
RG 190.62
DW 20.800 usec
DE 6.50 usec
TE 297.9 K
D1 2.00000000 sec
D11 0.03000000 sec
======== CHANNEL f1 ========
SFO1 100.6429474 MHz
NUC1 13C
P1 10.00 usec
PLW1 65.00000000 W
======== CHANNEL f2 ========
SFO2 400.2116008 MHz
NUC2 1H
CPDPRG[2 waltz16
PCPD2 90.00 usec
PLW2 13.00000000 W
PLW12 0.36111000 W
PLW13 0.29249999 W
F2 - Processing parameters
SI 32768
SF 100.6328850 MHz
WDW EM
SSB 0
LB 1.00 Hz
GB 0
PC 1.40
200 180 160 140 120 100 80 60 40 20 ppm

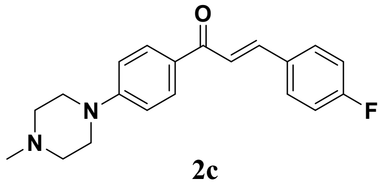

2c

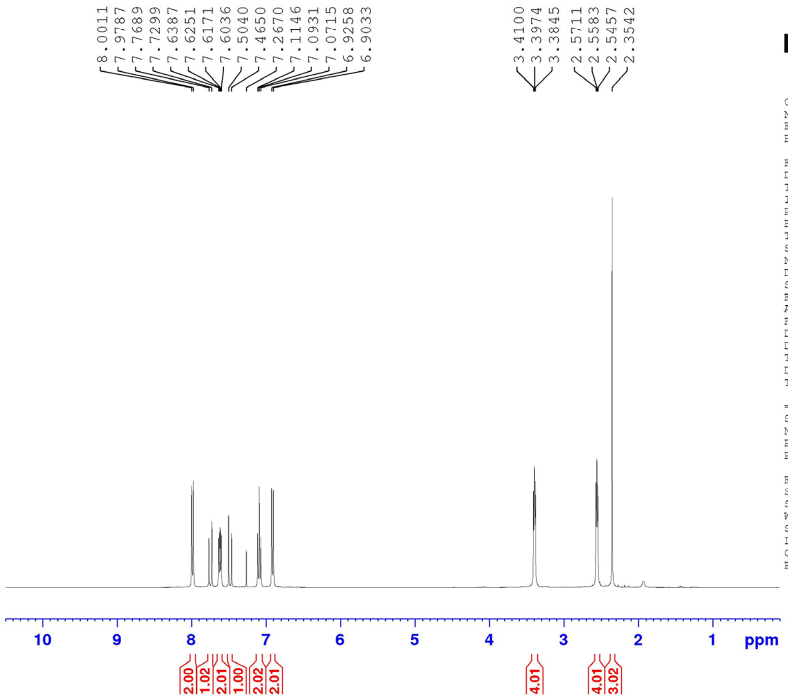


```
Current Data Parameters
NAME             AKA-IX-19
EXPNO                    1
PROCNO                   1

F2 - Acquisition Parameters
Date_             20211115
Time                 11.35
INSTRUM              spect
PROBHD    5 mm PABBO BB/
PULPROG               zg30
TD                   65536
SOLVENT              CDCl3
NS                      16
DS                       2
SWH             8012.820 Hz
FIDRES          0.122266 Hz
AQ             4.0894465 sec
RG                   91.91
DW                62.400 usec
DE                  6.50 usec
TE                 297.7 K
D1            1.00000000 sec
TD0                      1

======== CHANNEL f1 ========
SFO1         400.2124715 MHz
NUC1                    1H
P1                 16.00 usec
PLW1         13.00000000 W

F2 - Processing parameters
SI                   65536
SF           400.2100102 MHz
WDW                     EM
SSB                      0
LB                  0.30 Hz
GB                       0
PC                    1.00
```

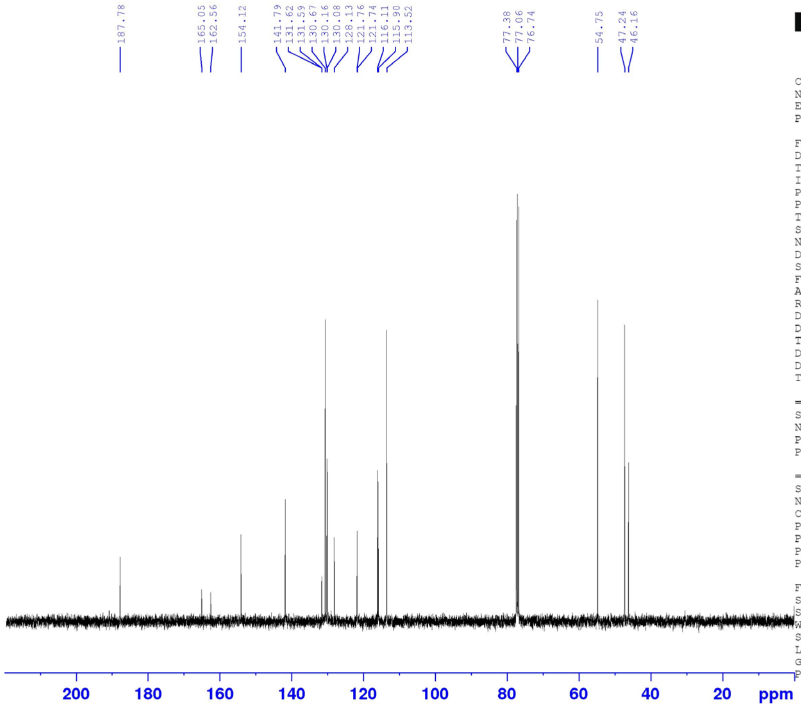


```
Current Data Parameters
NAME             AKA-IX-19
EXPNO                    2
PROCNO                   1

F2 - Acquisition Parameters
Date_             20211115
Time                 11.43
INSTRUM              spect
PROBHD    5 mm PABBO BB/
PULPROG            zgpg30
TD                   65536
SOLVENT              CDCl3
NS                     100
DS                       4
SWH            24038.461 Hz
FIDRES          0.366798 Hz
AQ             1.3631488 sec
RG                  190.62
DW                20.800 usec
DE                  6.50 usec
TE                 297.9 K
D1            2.00000000 sec
D11           0.03000000 sec
TD0                      1

======== CHANNEL f1 ========
SFO1         100.6429474 MHz
NUC1                   13C
P1                 10.00 usec
PLW1         65.00000000 W

======== CHANNEL f2 ========
SFO2         400.2116008 MHz
NUC2                    1H
CPDPRG[2           waltz16
PCPD2              90.00 usec
PLW2         13.00000000 W
PLW12         0.36111000 W
PLW13         0.29249999 W

F2 - Processing parameters
SI                   32768
SF           100.6328850 MHz
WDW                     EM
SSB                      0
LB                  1.00 Hz
GB                       0
PC                    1.40
```

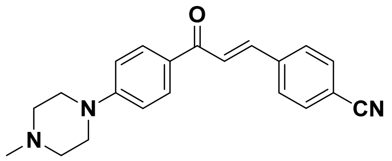

2d

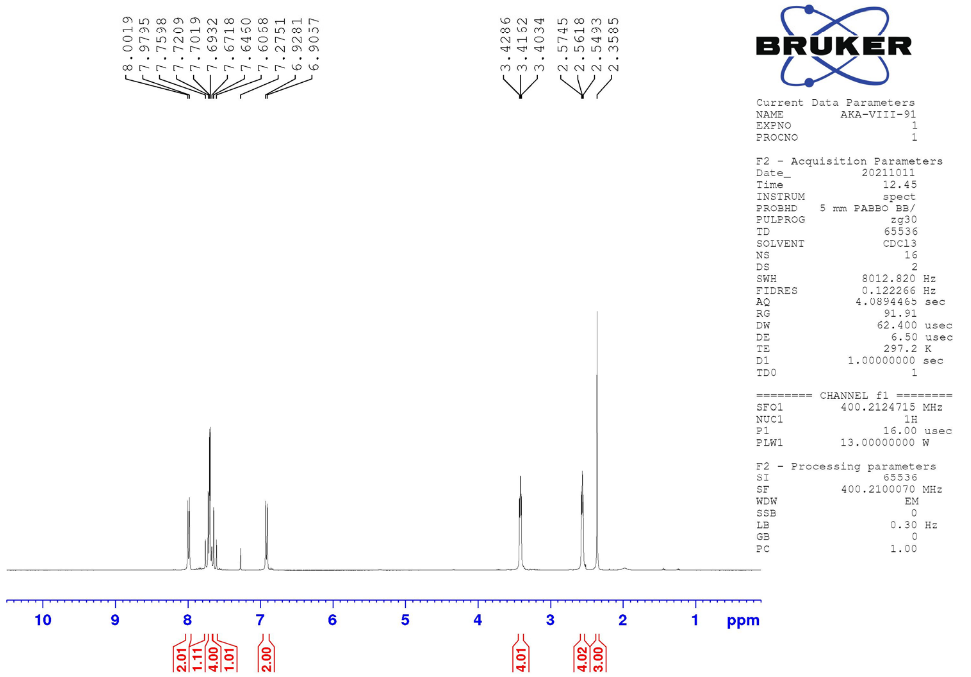

8.0019
7.9795
7.7598
7.7209
7.7019
7.6932
7.6718
7.6450
7.6068
7.2751
6.9281
6.9057
3.4286
3.4162
3.4034
2.5745
2.5618
2.5493
2.3585
BRUKER
Current Data Parameters
NAME AKA-VIII-91
EXPNO 1
PROCNO 1
F2 - Acquisition Parameters
Date_ 20211011
Time 12.45
INSTRUM spect
PROBHD 5 mm PABBO BB/
PULPROG zg30
TD 65536
SOLVENT CDCl3
NS 16
DS 2
SWH 8012.820 Hz
FIDRES 0.122266 Hz
AQ 4.0894465 sec
RG 91.91
DW 62.400 usec
DE 6.50 usec
TE 297.2 K
D1 1.00000000 sec
TD0 1
======== CHANNEL f1 ========
SFO1 400.2124715 MHz
NUC1 1H
P1 16.00 usec
PLW1 13.00000000 W
F2 - Processing parameters
SI 65536
SF 400.2100070 MHz
WDW EM
SSB 0
LB 0.30 Hz
GB 0
PC 1.00
10
9
8
7
6
5
4
3
2
1
ppm
2.01
1.11
4.00
1.01
2.00
4.01
4.02
3.00

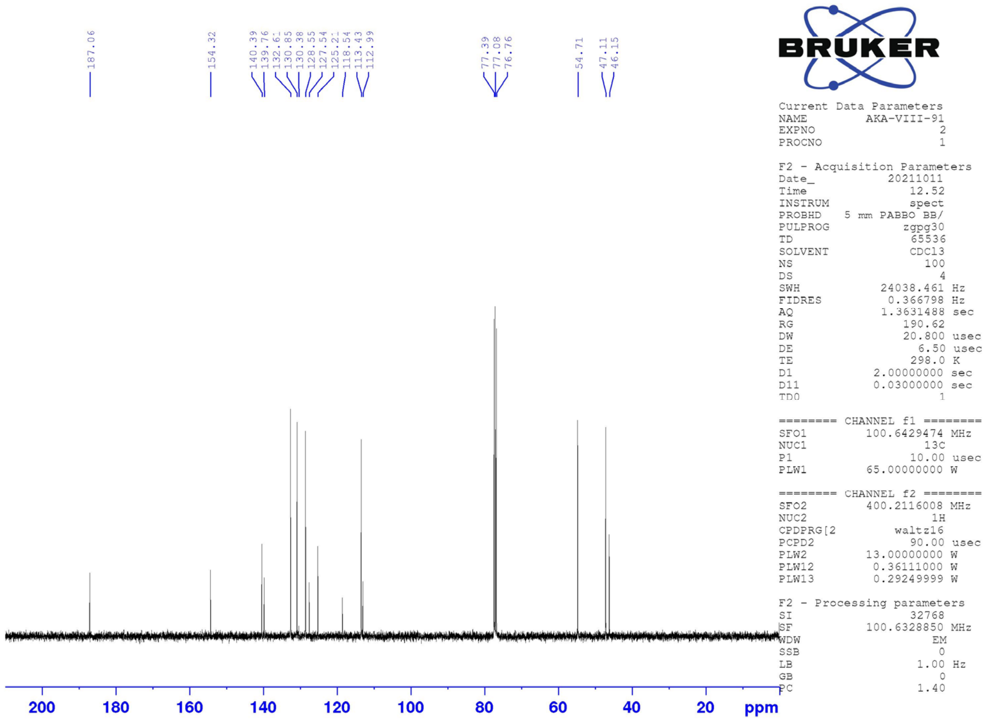

187.06
154.32
140.39
139.76
132.6:
130.8:
130.38
128.55
127.54
125.2:
118.54
113.43
112.99
77.39
77.08
76.76
54.71
47.11
46.15
BRUKER
Current Data Parameters
NAME AKA-VIII-91
EXPNO 2
PROCNO 1
F2 - Acquisition Parameters
Date_ 20211011
Time 12.52
INSTRUM spect
PROBHD 5 mm PABBO BB/
PULPROG zgpg30
TD 65536
SOLVENT CDCl3
NS 100
DS 4
SWH 24038.461 Hz
FIDRES 0.366798 Hz
AQ 1.3631488 sec
RG 190.62
DW 20.800 usec
DE 6.50 usec
TE 298.0 K
D1 2.00000000 sec
D11 0.03000000 sec
TD0 1
======== CHANNEL f1 ========
SFO1 100.6429474 MHz
NUC1 13C
P1 10.00 usec
PLW1 65.00000000 W
======== CHANNEL f2 ========
SFO2 400.2116008 MHz
NUC2 1H
CPDPRG[2 waltz16
PCPD2 90.00 usec
PLW2 13.00000000 W
PLW12 0.36111000 W
PLW13 0.29249999 W
F2 - Processing parameters
SI 32768
SF 100.6328850 MHz
WDW EM
SSB 0
LB 1.00 Hz
GB 0
PC 1.40
200
180
160
140
120
100
80
60
40
20
ppm

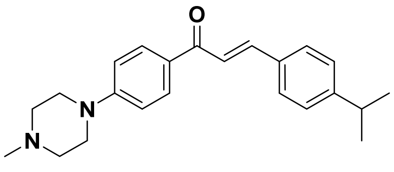
O
N
N

2e

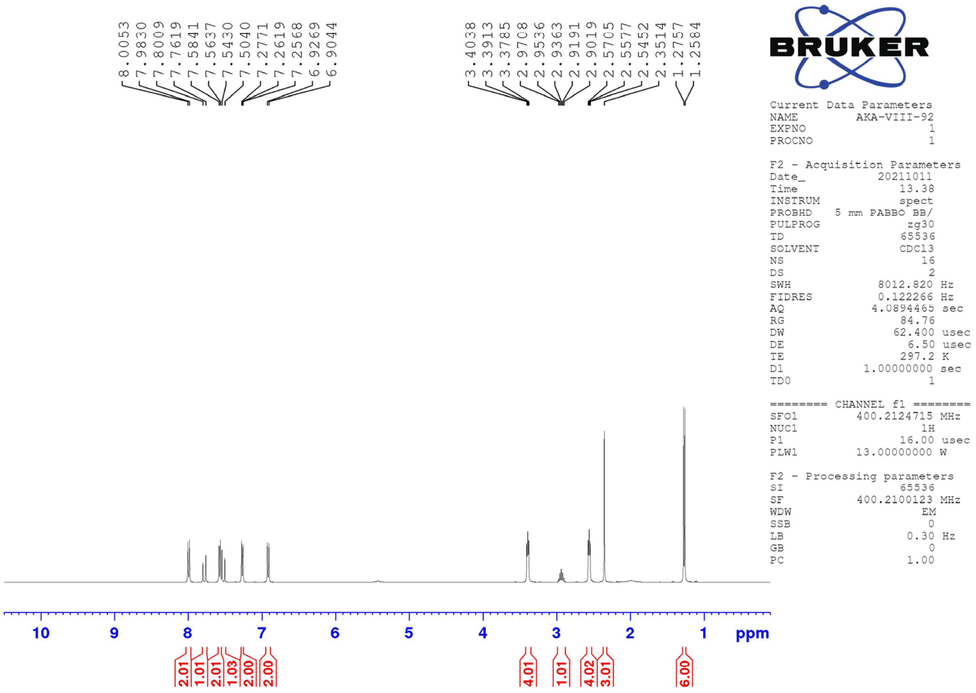
8.0053
7.9830
7.8009
7.7619
7.5841
7.5637
7.5430
7.5040
7.2771
7.2619
7.2568
6.9269
6.9044
3.4038
3.3913
3.3785
2.9708
2.9536
2.9363
2.9191
2.9019
2.5705
2.5577
2.5452
2.3514
1.2757
1.2584
BRUKER
Current Data Parameters
NAME AKA-VIII-92
EXPNO 1
PROCNO 1
F2 - Acquisition Parameters
Date_ 20211011
Time 13.38
INSTRUM spect
PROBHD 5 mm PABBO BB/
PULPROG zg30
TD 65536
SOLVENT CDCl3
NS 16
DS 2
SWH 8012.820 Hz
FIDRES 0.122266 Hz
AQ 4.0894465 sec
RG 84.76
DW 62.400 usec
DE 6.50 usec
TE 297.2 K
D1 1.00000000 sec
TD0 1
======== CHANNEL f1 ========
SFO1 400.2124715 MHz
NUC1 1H
P1 16.00 usec
PLW1 13.00000000 W
F2 - Processing parameters
SI 65536
SF 400.2100123 MHz
WDW EM
SSB 0
LB 0.30 Hz
GB 0
PC 1.00
10
9
8
7
6
5
4
3
2
1
ppm
2.01
1.01
2.01
1.03
2.00
2.00
4.01
1.01
4.02
3.01
6.00

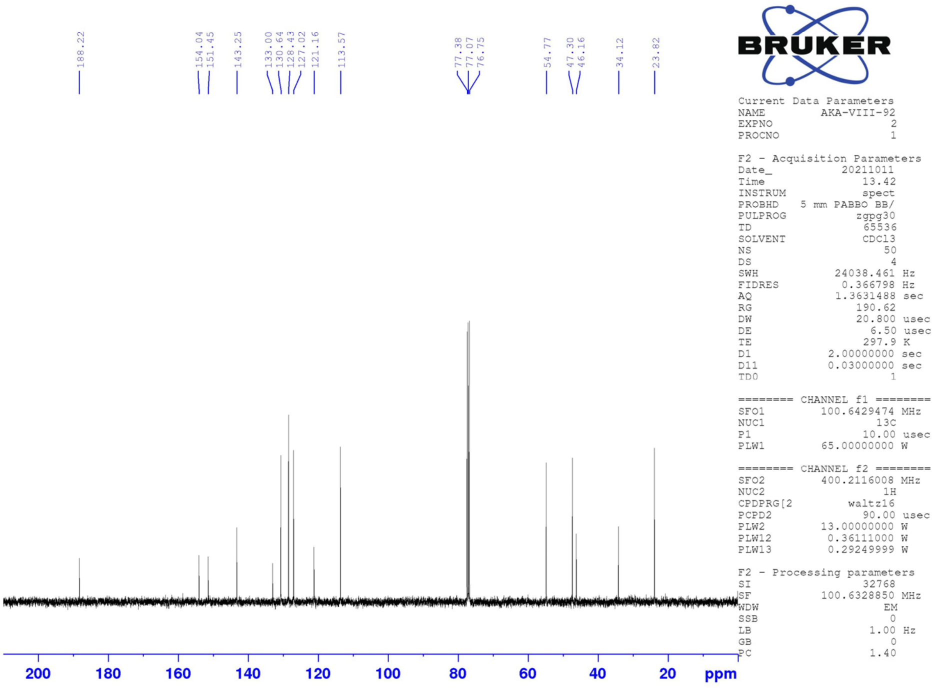
188.22
154.04
151.45
143.25
133.00
130.64
128.43
127.02
121.16
113.57
77.38
77.07
76.75
54.77
47.30
46.16
34.12
23.82
BRUKER
Current Data Parameters
NAME AKA-VIII-92
EXPNO 2
PROCNO 1
F2 - Acquisition Parameters
Date_ 20211011
Time 13.42
INSTRUM spect
PROBHD 5 mm PABBO BB/
PULPROG zgpg30
TD 65536
SOLVENT CDCl3
NS 50
DS 4
SWH 24038.461 Hz
FIDRES 0.366798 Hz
AQ 1.3631488 sec
RG 190.62
DW 20.800 usec
DE 6.50 usec
TE 297.9 K
D1 2.00000000 sec
D11 0.03000000 sec
TD0 1
======== CHANNEL f1 ========
SFO1 100.6429474 MHz
NUC1 13C
P1 10.00 usec
PLW1 65.00000000 W
======== CHANNEL f2 ========
SFO2 400.2116008 MHz
NUC2 1H
CPDPRG[2 waltz16
PCPD2 90.00 usec
PLW2 13.00000000 W
PLW12 0.36111000 W
PLW13 0.29249999 W
F2 - Processing parameters
SI 32768
SF 100.6328850 MHz
WDW EM
SSB 0
LB 1.00 Hz
GB 0
PC 1.40
200
180
160
140
120
100
80
60
40
20
ppm

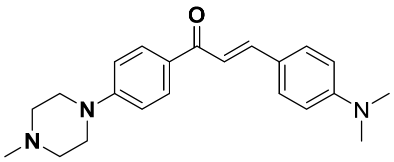

2f

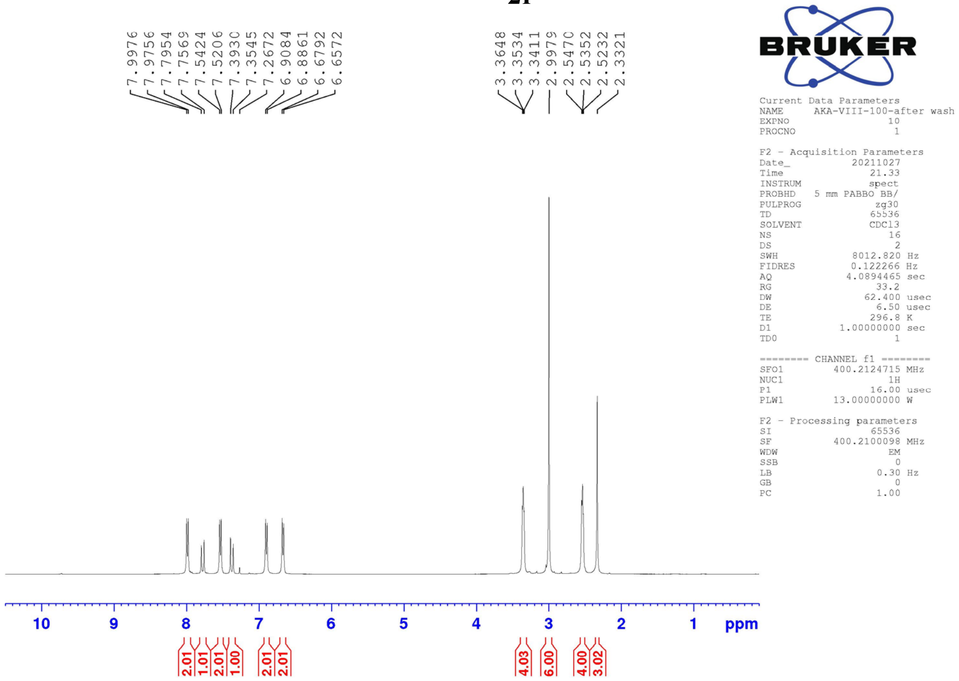

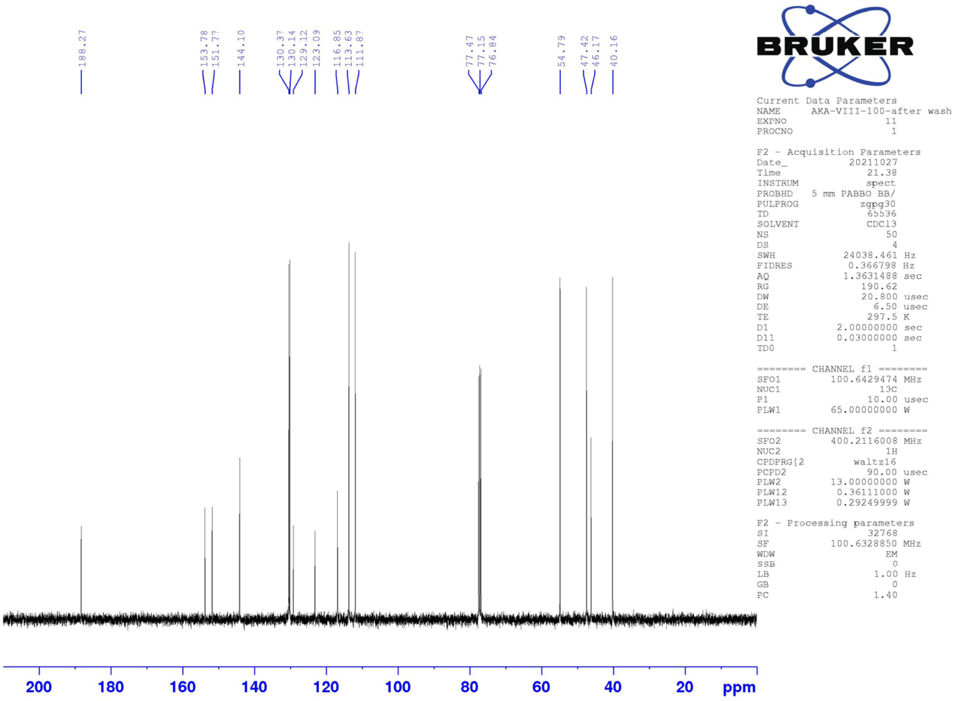

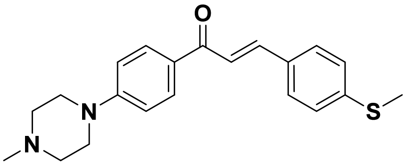

2g

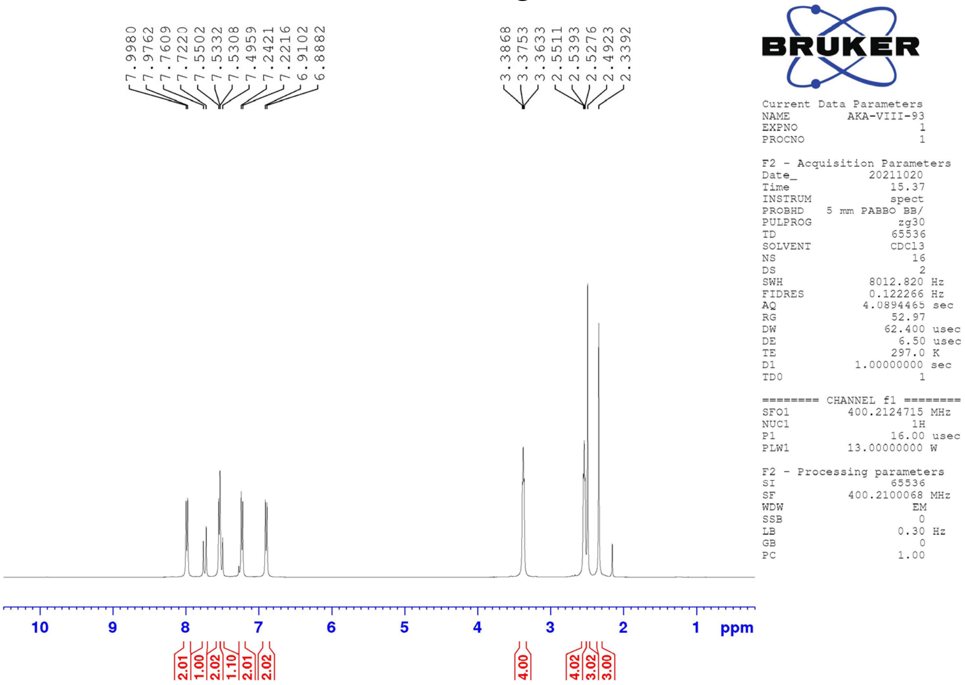
7.9980
7.9762
7.7609
7.7220
7.5502
7.5332
7.5308
7.4959
7.2421
7.2216
6.9102
6.8882
3.3868
3.3753
3.3633
2.5511
2.5393
2.5276
2.4923
2.3392
BRUKER
Current Data Parameters
NAME AKA-VIII-93
EXPNO 1
PROCNO 1
F2 - Acquisition Parameters
Date_ 20211020
Time 15.37
INSTRUM spect
PROBHD 5 mm PABBO BB/
PULPROG zg30
TD 65536
SOLVENT CDCl3
NS 16
DS 2
SWH 8012.820 Hz
FIDRES 0.122266 Hz
AQ 4.0894465 sec
RG 52.97
DW 62.400 usec
DE 6.50 usec
TE 297.0 K
D1 1.00000000 sec
TD0 1
======== CHANNEL f1 ========
SFO1 400.2124715 MHz
NUC1 1H
P1 16.00 usec
PLW1 13.00000000 W
F2 - Processing parameters
SI 65536
SF 400.2100068 MHz
WDW EM
SSB 0
LB 0.30 Hz
GB 0
PC 1.00
10 9 8 7 6 5 4 3 2 1 ppm
2.01 1.00 2.02 1.10 2.01 2.02
4.00
4.02 3.02 3.00

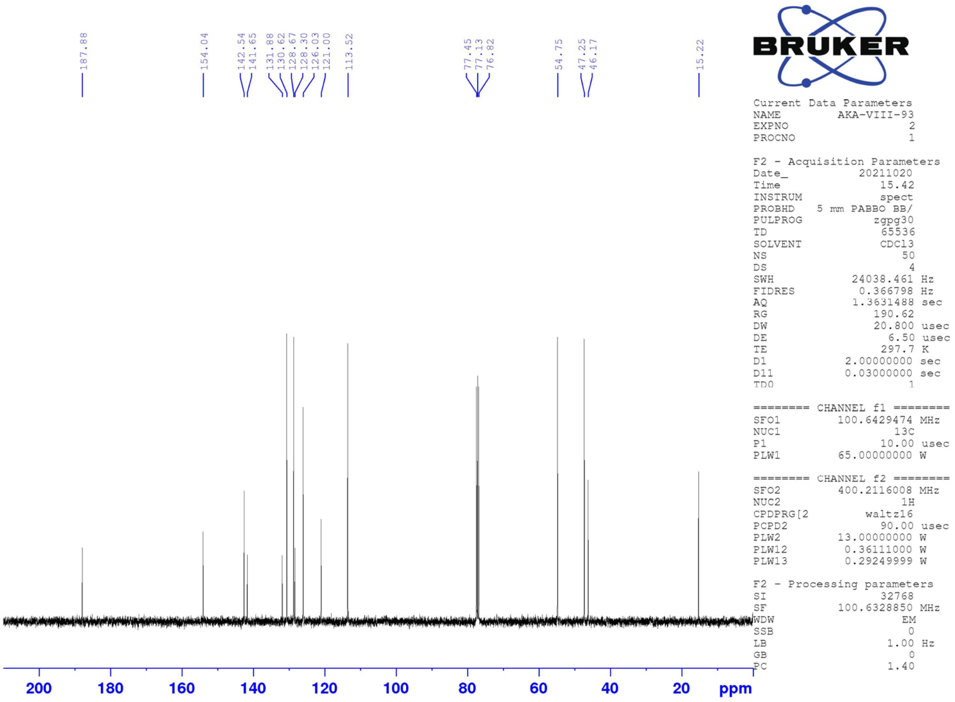
187.88
154.04
142.54
141.65
131.88
130.62
128.67
128.30
126.03
121.00
113.52
77.45
77.13
76.82
54.75
47.25
46.17
15.22
BRUKER
Current Data Parameters
NAME AKA-VIII-93
EXPNO 2
PROCNO 1
F2 - Acquisition Parameters
Date_ 20211020
Time 15.42
INSTRUM spect
PROBHD 5 mm PABBO BB/
PULPROG zgpg30
TD 65536
SOLVENT CDCl3
NS 50
DS 4
SWH 24038.461 Hz
FIDRES 0.366798 Hz
AQ 1.3631488 sec
RG 190.62
DW 20.800 usec
DE 6.50 usec
TE 297.7 K
D1 2.00000000 sec
D11 0.03000000 sec
TD0 1
======== CHANNEL f1 ========
SFO1 100.6429474 MHz
NUC1 13C
P1 10.00 usec
PLW1 65.00000000 W
======== CHANNEL f2 ========
SFO2 400.2116008 MHz
NUC2 1H
CPDPRG[2 waltz16
PCPD2 90.00 usec
PLW2 13.00000000 W
PLW12 0.36111000 W
PLW13 0.29249999 W
F2 - Processing parameters
SI 32768
SF 100.6328850 MHz
WDW EM
SSB 0
LB 1.00 Hz
GB 0
PC 1.40
200 180 160 140 120 100 80 60 40 20 ppm

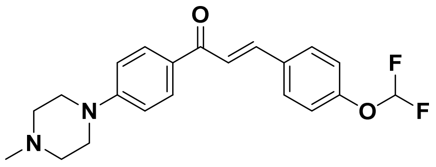

2h

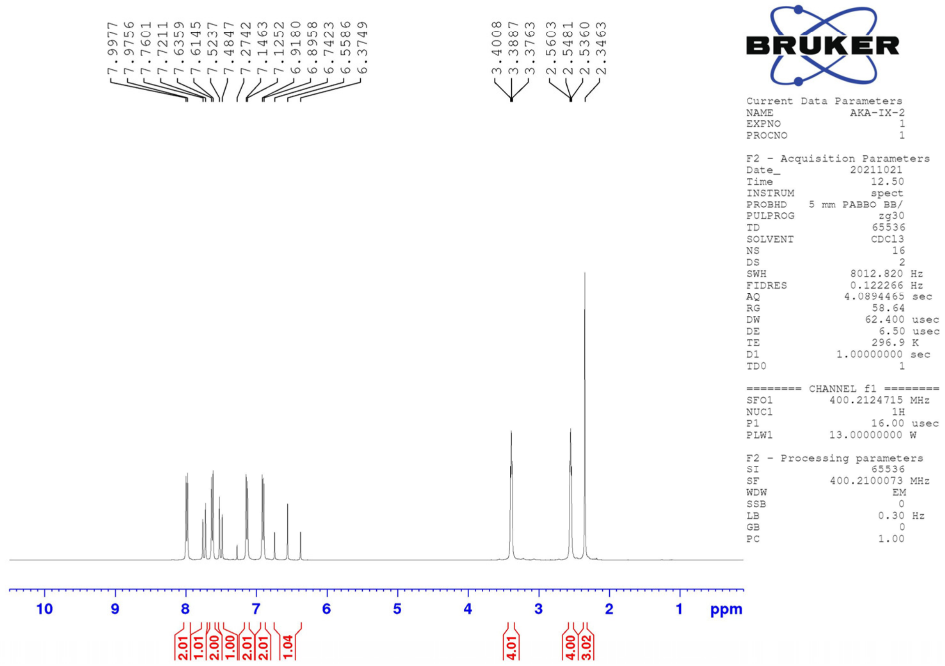

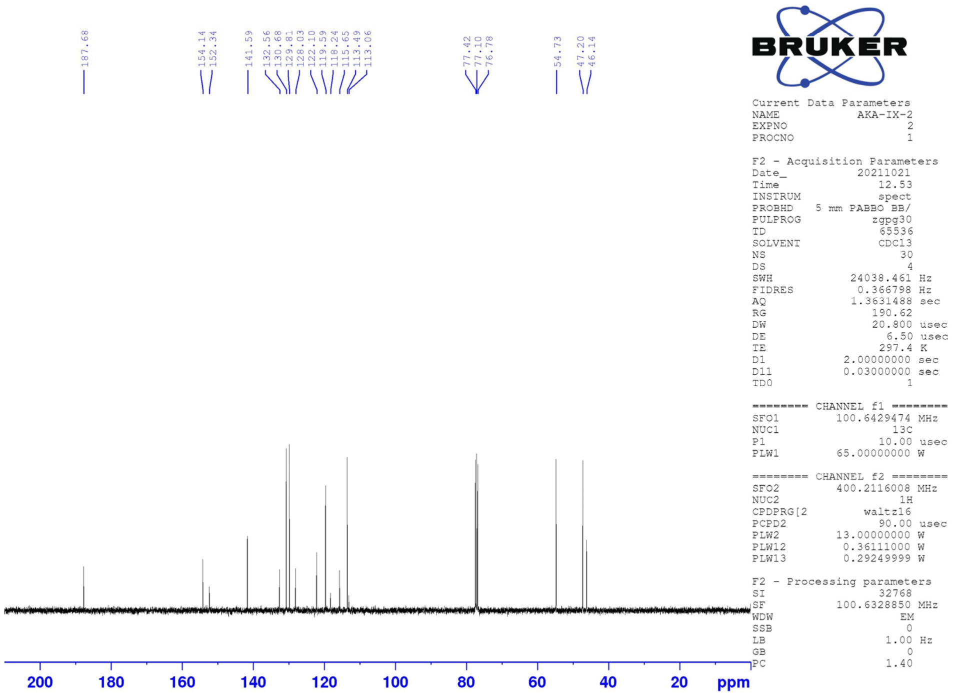

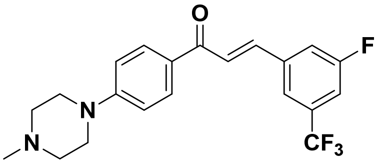

2i

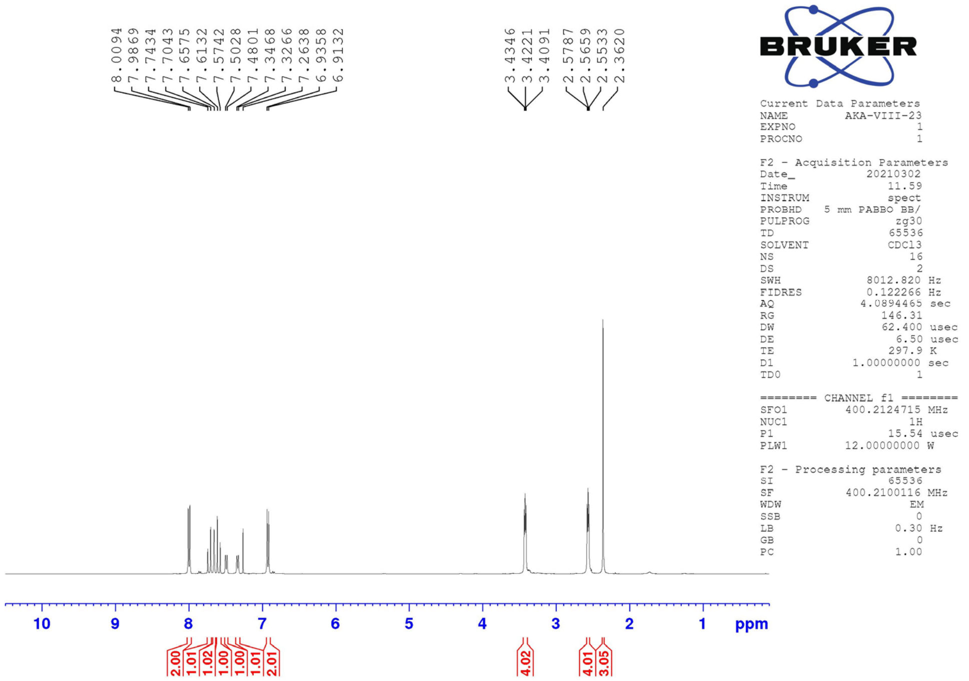

8.0094
7.9869
7.7434
7.7043
7.6575
7.6132
7.5742
7.5028
7.4801
7.3468
7.3266
7.2638
6.9358
6.9132
3.4346
3.4221
3.4091
2.5787
2.5659
2.5533
2.3620
BRUKER
Current Data Parameters
NAME AKA-VIII-23
EXPNO 1
PROCNO 1
F2 - Acquisition Parameters
Date_ 20210302
Time 11.59
INSTRUM spect
PROBHD 5 mm PABBO BB/
PULPROG zg30
TD 65536
SOLVENT CDCl3
NS 16
DS 2
SWH 8012.820 Hz
FIDRES 0.122266 Hz
AQ 4.0894465 sec
RG 146.31
DW 62.400 usec
DE 6.50 usec
TE 297.9 K
D1 1.00000000 sec
TD0 1
======== CHANNEL f1 ========
SFO1 400.2124715 MHz
NUC1 1H
P1 15.54 usec
PLW1 12.00000000 W
F2 - Processing parameters
SI 65536
SF 400.2100116 MHz
WDW EM
SSB 0
LB 0.30 Hz
GB 0
PC 1.00
10
9
8
7
6
5
4
3
2
1
ppm
2.00
1.01
1.02
1.00
1.00
1.01
2.01
4.02
4.01
3.05

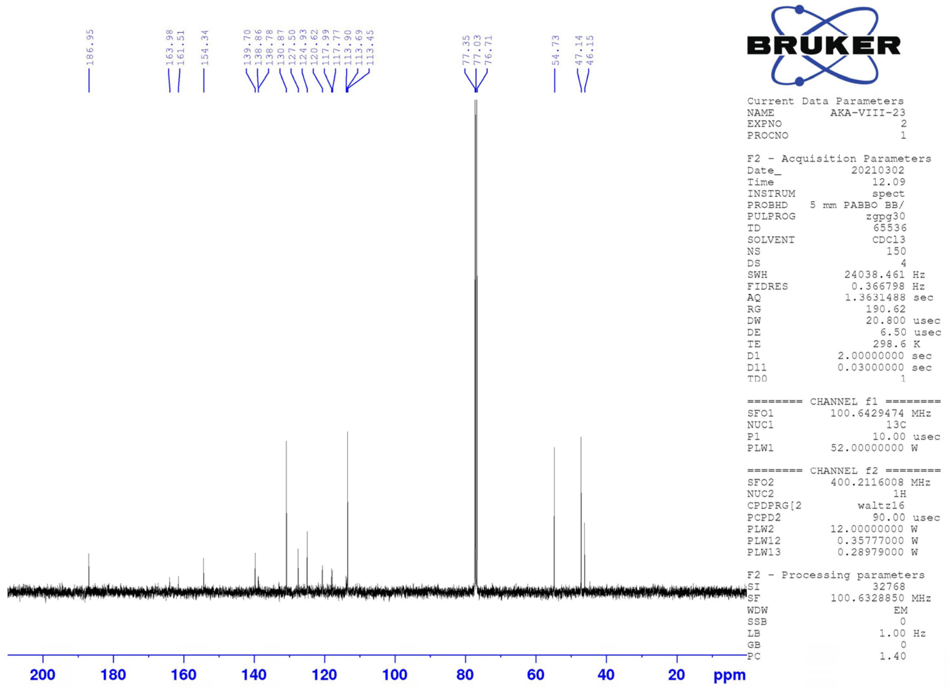

186.95
163.98
161.51
154.34
139.70
138.86
138.78
130.87
127.50
124.93
120.62
117.99
117.77
113.90
113.69
113.45
77.35
77.03
76.71
54.73
47.14
46.15
BRUKER
Current Data Parameters
NAME AKA-VIII-23
EXPNO 2
PROCNO 1
F2 - Acquisition Parameters
Date_ 20210302
Time 12.09
INSTRUM spect
PROBHD 5 mm PABBO BB/
PULPROG zgpg30
TD 65536
SOLVENT CDCl3
NS 150
DS 4
SWH 24038.461 Hz
FIDRES 0.366798 Hz
AQ 1.3631488 sec
RG 190.62
DW 20.800 usec
DE 6.50 usec
TE 298.6 K
D1 2.00000000 sec
D11 0.03000000 sec
TD0 1
======== CHANNEL f1 ========
SFO1 100.6429474 MHz
NUC1 13C
P1 10.00 usec
PLW1 52.00000000 W
======== CHANNEL f2 ========
SFO2 400.2116008 MHz
NUC2 1H
CPDPRG[2 waltz16
PCPD2 90.00 usec
PLW2 12.00000000 W
PLW12 0.35777000 W
PLW13 0.28979000 W
F2 - Processing parameters
SI 32768
SF 100.6328850 MHz
WDW EM
SSB 0
LB 1.00 Hz
GB 0
PC 1.40
200
180
160
140
120
100
80
60
40
20
ppm

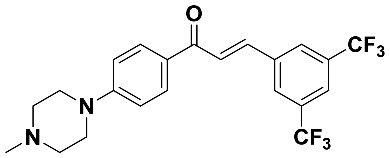

2j

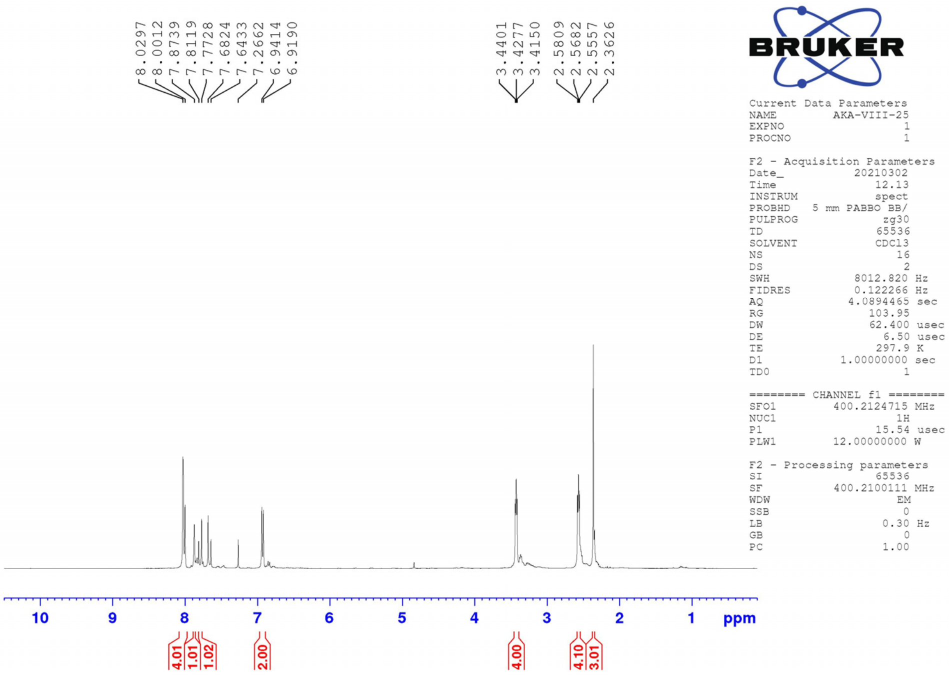

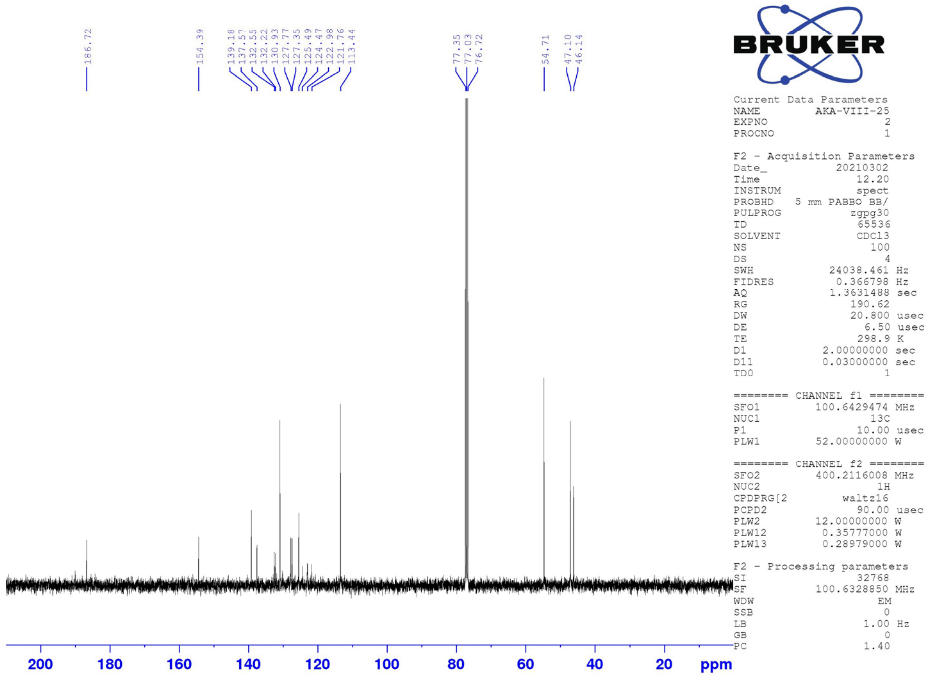

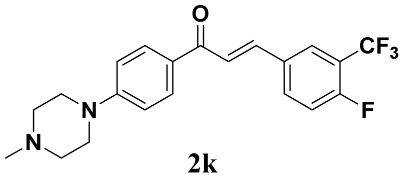

2k

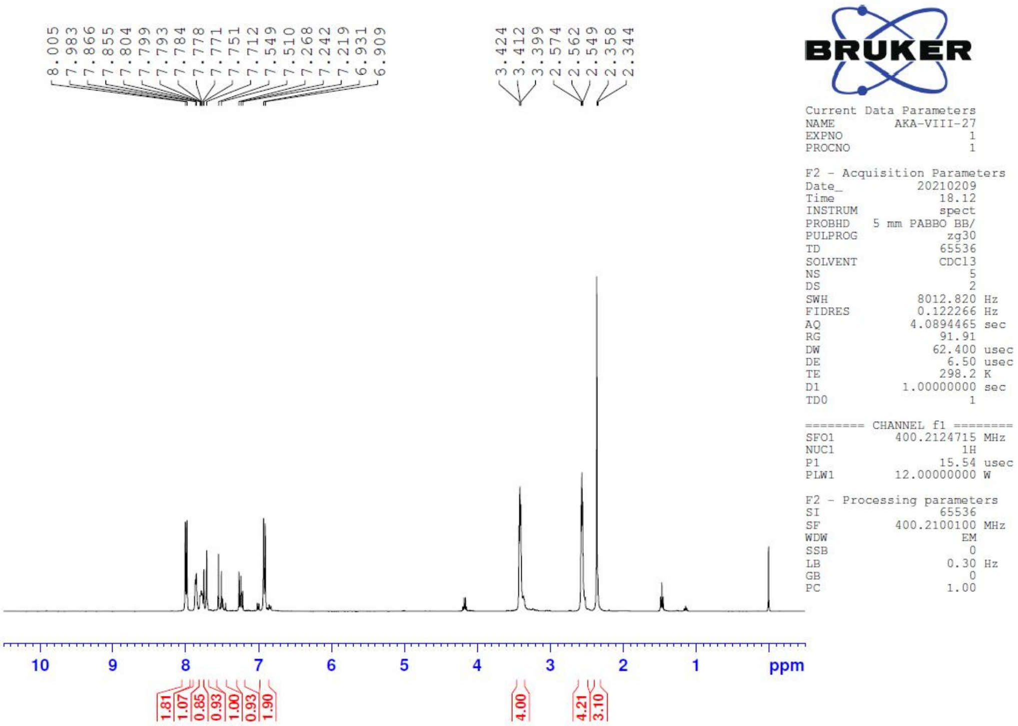
8.005
7.983
7.866
7.855
7.804
7.799
7.793
7.784
7.778
7.771
7.751
7.712
7.549
7.510
7.268
7.242
7.219
6.931
6.909
3.424
3.412
3.399
2.574
2.562
2.549
2.358
2.344
BRUKER
Current Data Parameters
NAME AKA-VIII-27
EXPNO 1
PROCNO 1
F2 - Acquisition Parameters
Date_ 20210209
Time 18.12
INSTRUM spect
PROBHD 5 mm PABBO BB/
PULPROG zg30
TD 65536
SOLVENT CDCl3
NS 5
DS 2
SWH 8012.820 Hz
FIDRES 0.122266 Hz
AQ 4.0894465 sec
RG 91.91
DW 62.400 usec
DE 6.50 usec
TE 298.2 K
D1 1.00000000 sec
TD0 1
======== CHANNEL f1 ========
SFO1 400.2124715 MHz
NUC1 1H
P1 15.54 usec
PLW1 12.00000000 W
F2 - Processing parameters
SI 65536
SF 400.2100100 MHz
WDW EM
SSB 0
LB 0.30 Hz
GB 0
PC 1.00
10
9
8
7
6
5
4
3
2
1
ppm
1.81
1.07
0.85
0.93
1.00
0.93
1.90
4.00
4.21
3.10

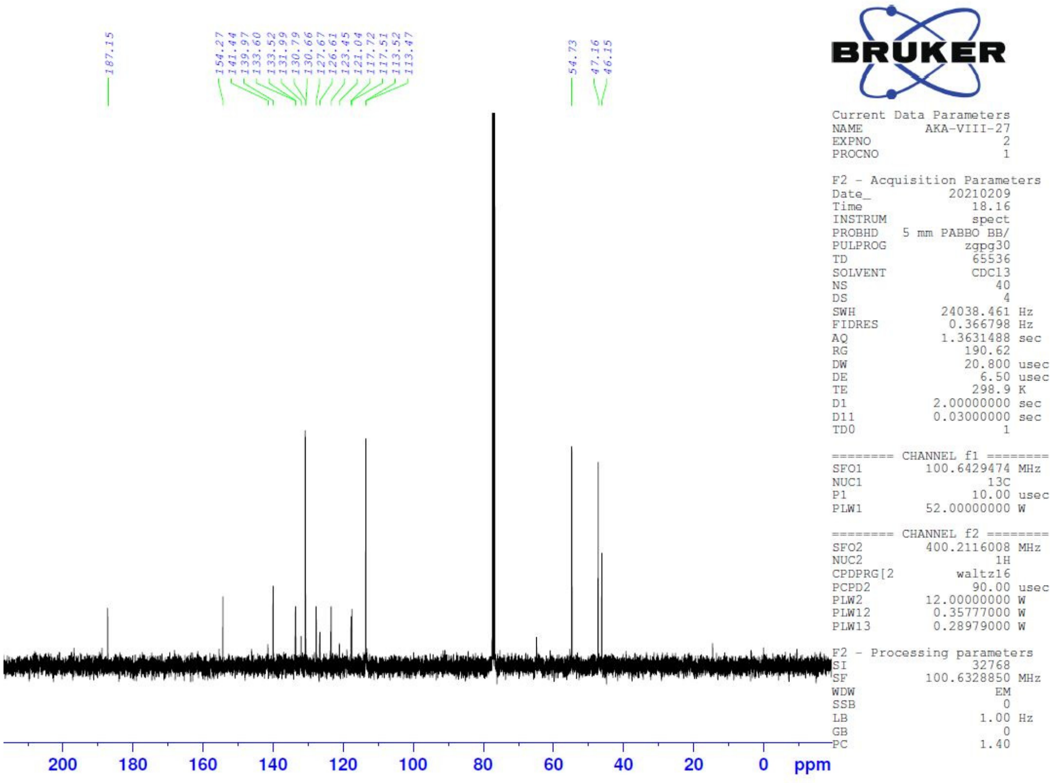
187.15
154.27
141.44
139.97
133.60
133.52
131.99
130.79
130.66
127.67
126.61
123.45
121.04
117.72
117.51
113.52
113.47
54.73
47.16
46.15
BRUKER
Current Data Parameters
NAME AKA-VIII-27
EXPNO 2
PROCNO 1
F2 - Acquisition Parameters
Date_ 20210209
Time 18.16
INSTRUM spect
PROBHD 5 mm PABBO BB/
PULPROG zgpg30
TD 65536
SOLVENT CDCl3
NS 40
DS 4
SWH 24038.461 Hz
FIDRES 0.366798 Hz
AQ 1.3631488 sec
RG 190.62
DW 20.800 usec
DE 6.50 usec
TE 298.9 K
D1 2.00000000 sec
D11 0.03000000 sec
TD0 1
======== CHANNEL f1 ========
SFO1 100.6429474 MHz
NUC1 13C
P1 10.00 usec
PLW1 52.00000000 W
======== CHANNEL f2 ========
SFO2 400.2116008 MHz
NUC2 1H
CPDPRG[2 waltz16
PCPD2 90.00 usec
PLW2 12.00000000 W
PLW12 0.35777000 W
PLW13 0.28979000 W
F2 - Processing parameters
SI 32768
SF 100.6328850 MHz
WDW EM
SSB 0
LB 1.00 Hz
GB 0
PC 1.40
200
180
160
140
120
100
80
60
40
20
0
ppm

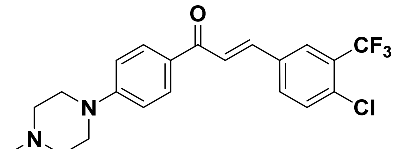
O
CF3
N
N
Cl

2l

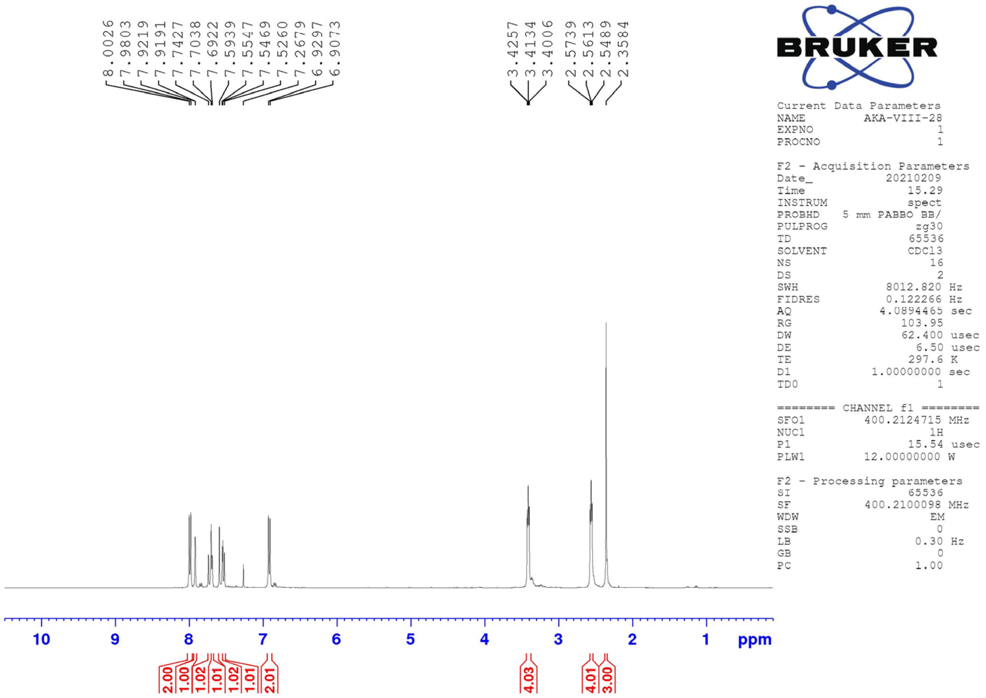
8.0026
7.9803
7.9219
7.9191
7.7427
7.7038
7.6922
7.5939
7.5547
7.5469
7.5260
7.2679
6.9297
6.9073
3.4257
3.4134
3.4006
2.5739
2.5613
2.5489
2.3584
BRUKER
Current Data Parameters
NAME AKA-VIII-28
EXPNO 1
PROCNO 1
F2 - Acquisition Parameters
Date_ 20210209
Time 15.29
INSTRUM spect
PROBHD 5 mm PABBO BB/
PULPROG zg30
TD 65536
SOLVENT CDCl3
NS 16
DS 2
SWH 8012.820 Hz
FIDRES 0.122266 Hz
AQ 4.0894465 sec
RG 103.95
DW 62.400 usec
DE 6.50 usec
TE 297.6 K
D1 1.00000000 sec
TD0 1
======== CHANNEL f1 ========
SFO1 400.2124715 MHz
NUC1 1H
P1 15.54 usec
PLW1 12.00000000 W
F2 - Processing parameters
SI 65536
SF 400.2100098 MHz
WDW EM
SSB 0
LB 0.30 Hz
GB 0
PC 1.00
10 9 8 7 6 5 4 3 2 1 ppm
2.00
1.00
1.02
1.01
1.02
1.01
2.01
4.03
4.01
3.00

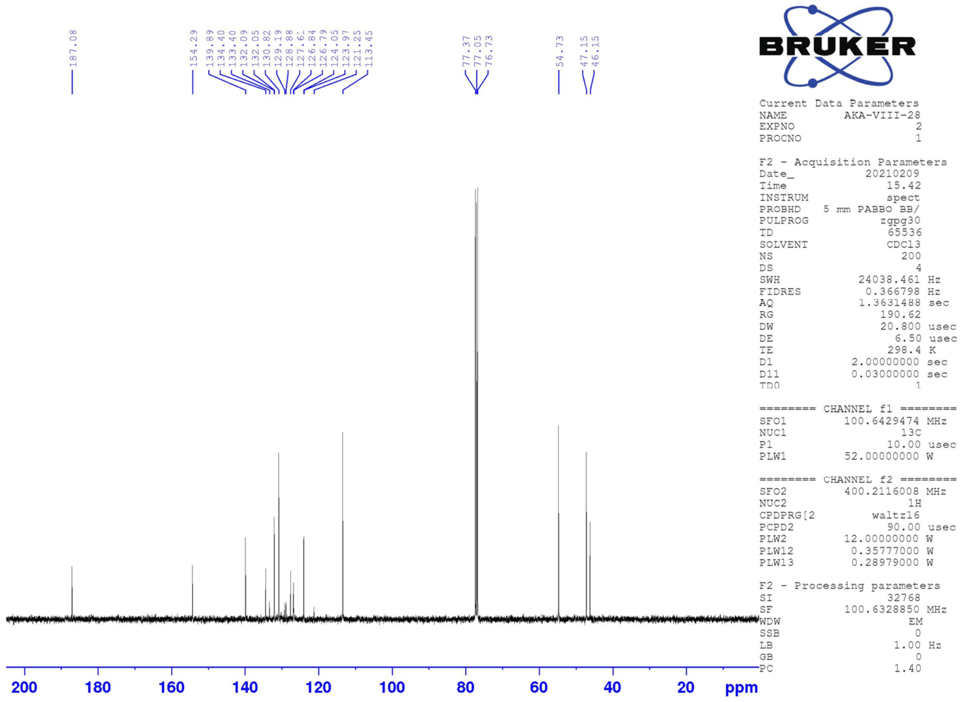
187.08
154.29
139.89
134.40
133.40
132.09
132.05
130.82
129.19
128.88
127.61
126.84
126.79
124.05
123.97
121.25
113.45
77.37
77.05
76.73
54.73
47.15
46.15
BRUKER
Current Data Parameters
NAME AKA-VIII-28
EXPNO 2
PROCNO 1
F2 - Acquisition Parameters
Date_ 20210209
Time 15.42
INSTRUM spect
PROBHD 5 mm PABBO BB/
PULPROG zgpg30
TD 65536
SOLVENT CDCl3
NS 200
DS 4
SWH 24038.461 Hz
FIDRES 0.366798 Hz
AQ 1.3631488 sec
RG 190.62
DW 20.800 usec
DE 6.50 usec
TE 298.4 K
D1 2.00000000 sec
D11 0.03000000 sec
TD0 1
======== CHANNEL f1 ========
SFO1 100.6429474 MHz
NUC1 13C
P1 10.00 usec
PLW1 52.00000000 W
======== CHANNEL f2 ========
SFO2 400.2116008 MHz
NUC2 1H
CPDPRG[2 waltz16
PCPD2 90.00 usec
PLW2 12.00000000 W
PLW12 0.35777000 W
PLW13 0.28979000 W
F2 - Processing parameters
SI 32768
SF 100.6328850 MHz
WDW EM
SSB 0
LB 1.00 Hz
GB 0
PC 1.40
200 180 160 140 120 100 80 60 40 20 ppm

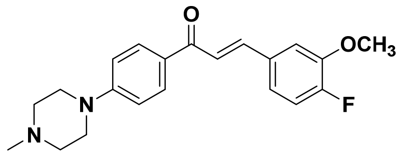
O
OCH3
N
N
F

2m

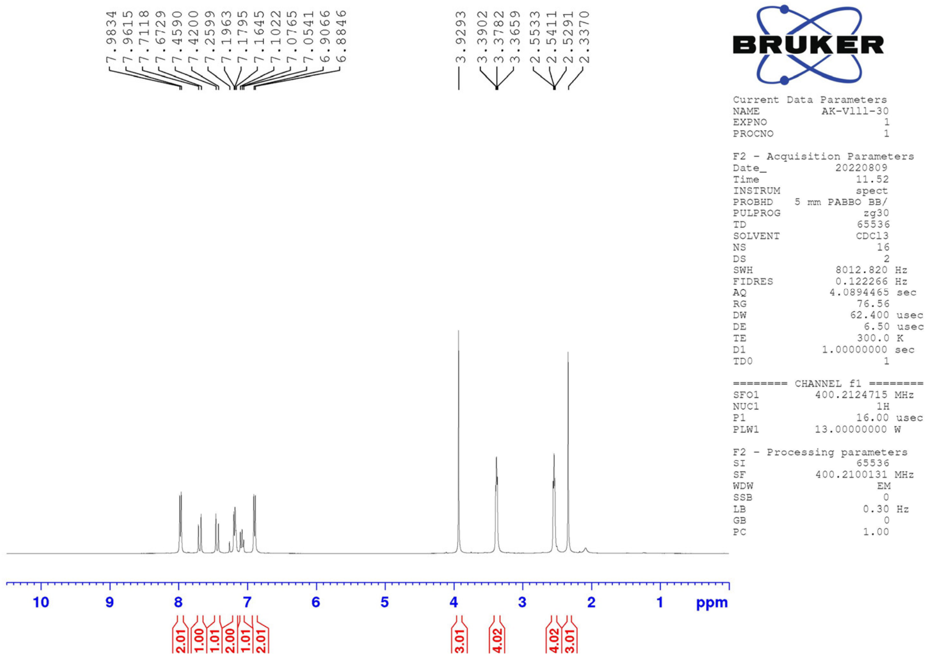
7.9834 7.9615 7.7118 7.6729 7.4590 7.4200 7.2599 7.1963 7.1795 7.1645 7.1022 7.0765 7.0541 6.9066 6.8846
3.9293 3.3902 3.3782 3.3659 2.5533 2.5411 2.5291 2.3370
10 9 8 7 6 5 4 3 2 1 ppm
2.01 1.00 1.01 2.00 1.01 2.01 3.01 4.02 4.02 3.01
BRUKER
Current Data Parameters
NAME AK-V111-30
EXPNO 1
PROCNO 1
F2 - Acquisition Parameters
Date_ 20220809
Time 11.52
INSTRUM spect
PROBHD 5 mm PABBO BB/
PULPROG zg30
TD 65536
SOLVENT CDCl3
NS 16
DS 2
SWH 8012.820 Hz
FIDRES 0.122266 Hz
AQ 4.0894465 sec
RG 76.56
DW 62.400 usec
DE 6.50 usec
TE 300.0 K
D1 1.00000000 sec
TD0 1
======== CHANNEL f1 ========
SFO1 400.2124715 MHz
NUC1 1H
P1 16.00 usec
PLW1 13.00000000 W
F2 - Processing parameters
SI 65536
SF 400.2100131 MHz
WDW EM
SSB 0
LB 0.30 Hz
GB 0
PC 1.00

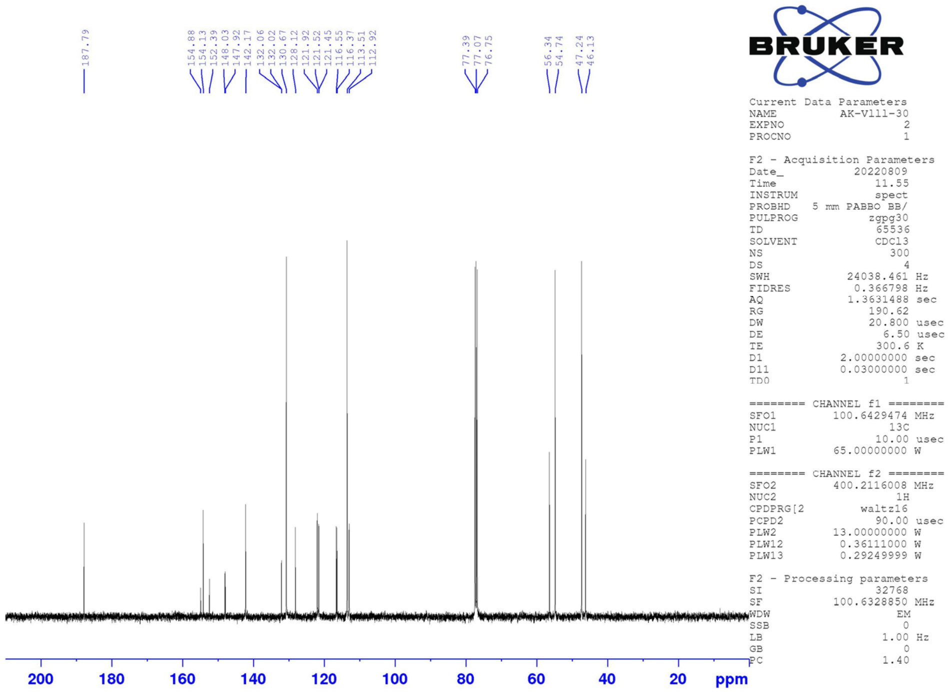
187.79
154.88 154.13 152.39 148.03 147.92 142.17 132.06 132.02 130.67 128.12 121.92 121.52 121.45 116.55 116.37 113.51 112.92
77.39 77.07 76.75
56.34 54.74
47.24 46.13
200 180 160 140 120 100 80 60 40 20 ppm
BRUKER
Current Data Parameters
NAME AK-V111-30
EXPNO 2
PROCNO 1
F2 - Acquisition Parameters
Date_ 20220809
Time 11.55
INSTRUM spect
PROBHD 5 mm PABBO BB/
PULPROG zgpg30
TD 65536
SOLVENT CDCl3
NS 300
DS 4
SWH 24038.461 Hz
FIDRES 0.366798 Hz
AQ 1.3631488 sec
RG 190.62
DW 20.800 usec
DE 6.50 usec
TE 300.6 K
D1 2.00000000 sec
D11 0.03000000 sec
TD0 1
======== CHANNEL f1 ========
SFO1 100.6429474 MHz
NUC1 13C
P1 10.00 usec
PLW1 65.00000000 W
======== CHANNEL f2 ========
SFO2 400.2116008 MHz
NUC2 1H
CPDPRG[2 waltz16
PCPD2 90.00 usec
PLW2 13.00000000 W
PLW12 0.36111000 W
PLW13 0.29249999 W
F2 - Processing parameters
SI 32768
SF 100.6328850 MHz
WDW EM
SSB 0
LB 1.00 Hz
GB 0
PC 1.40

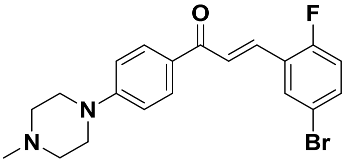
O
F
N
N
Br

2n

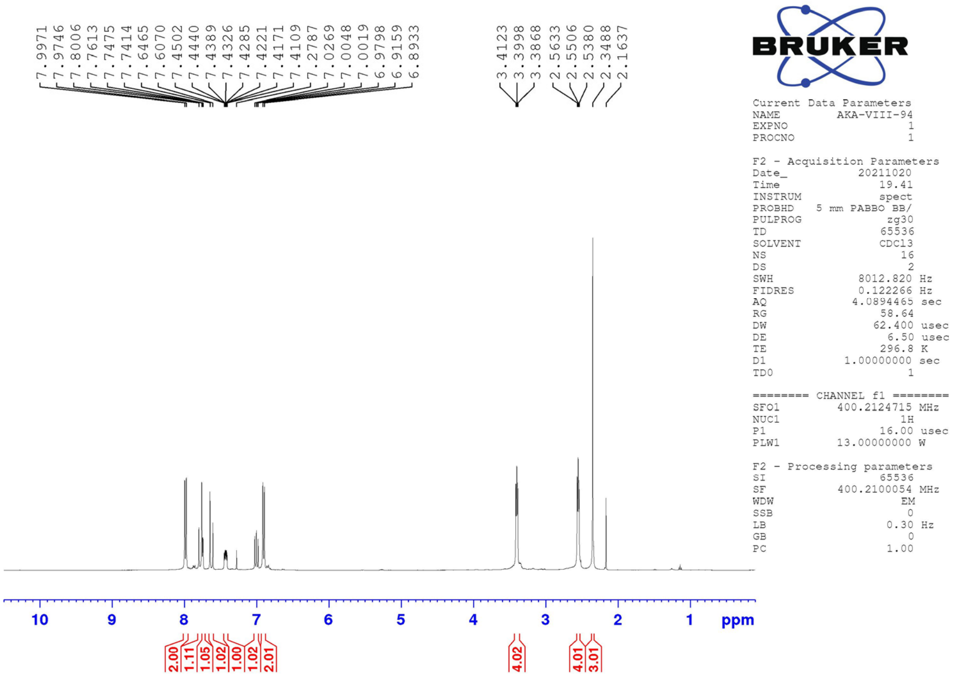
7.9971
7.9746
7.8006
7.7613
7.7475
7.7414
7.6465
7.6070
7.4502
7.4440
7.4389
7.4326
7.4285
7.4221
7.4171
7.4109
7.2787
7.0269
7.0048
7.0019
6.9798
6.9159
6.8933
3.4123
3.3998
3.3868
2.5633
2.5506
2.5380
2.3488
2.1637
BRUKER
Current Data Parameters
NAME AKA-VIII-94
EXPNO 1
PROCNO 1
F2 - Acquisition Parameters
Date_ 20211020
Time 19.41
INSTRUM spect
PROBHD 5 mm PABBO BB/
PULPROG zg30
TD 65536
SOLVENT CDCl3
NS 16
DS 2
SWH 8012.820 Hz
FIDRES 0.122266 Hz
AQ 4.0894465 sec
RG 58.64
DW 62.400 usec
DE 6.50 usec
TE 296.8 K
D1 1.00000000 sec
TD0 1
======== CHANNEL f1 ========
SFO1 400.2124715 MHz
NUC1 1H
P1 16.00 usec
PLW1 13.00000000 W
F2 - Processing parameters
SI 65536
SF 400.2100054 MHz
WDW EM
SSB 0
LB 0.30 Hz
GB 0
PC 1.00
10
9
8
7
6
5
4
3
2
1
ppm
2.00
1.11
1.05
1.02
1.00
1.02
2.01
4.02
4.01
3.01

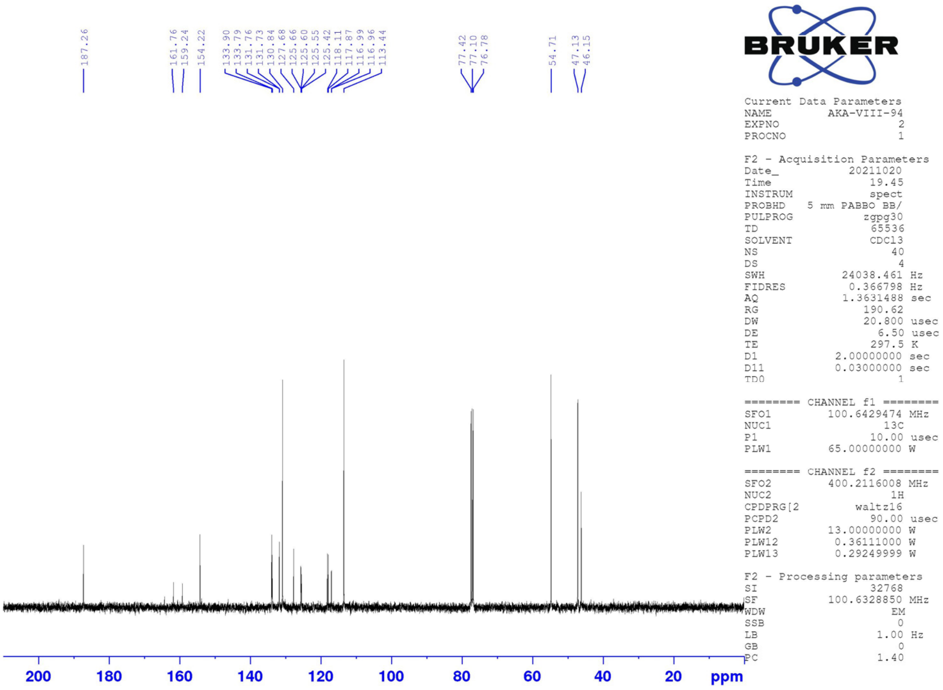
187.26
161.76
159.24
154.22
133.90
133.79
131.76
131.73
130.84
127.68
125.66
125.60
125.55
125.42
118.11
117.87
116.99
116.96
113.44
77.42
77.10
76.78
54.71
47.13
46.15
BRUKER
Current Data Parameters
NAME AKA-VIII-94
EXPNO 2
PROCNO 1
F2 - Acquisition Parameters
Date_ 20211020
Time 19.45
INSTRUM spect
PROBHD 5 mm PABBO BB/
PULPROG zgpg30
TD 65536
SOLVENT CDCl3
NS 40
DS 4
SWH 24038.461 Hz
FIDRES 0.366798 Hz
AQ 1.3631488 sec
RG 190.62
DW 20.800 usec
DE 6.50 usec
TE 297.5 K
D1 2.00000000 sec
D11 0.03000000 sec
TD0 1
======== CHANNEL f1 ========
SFO1 100.6429474 MHz
NUC1 13C
P1 10.00 usec
PLW1 65.00000000 W
======== CHANNEL f2 ========
SFO2 400.2116008 MHz
NUC2 1H
CPDPRG[2 waltz16
PCPD2 90.00 usec
PLW2 13.00000000 W
PLW12 0.36111000 W
PLW13 0.29249999 W
F2 - Processing parameters
SI 32768
SF 100.6328850 MHz
WDW EM
SSB 0
LB 1.00 Hz
GB 0
PC 1.40
200
180
160
140
120
100
80
60
40
20
ppm

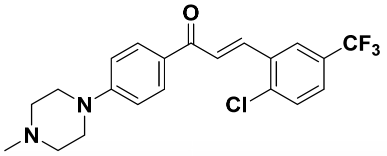

O
CF3
N
Cl
N

2o

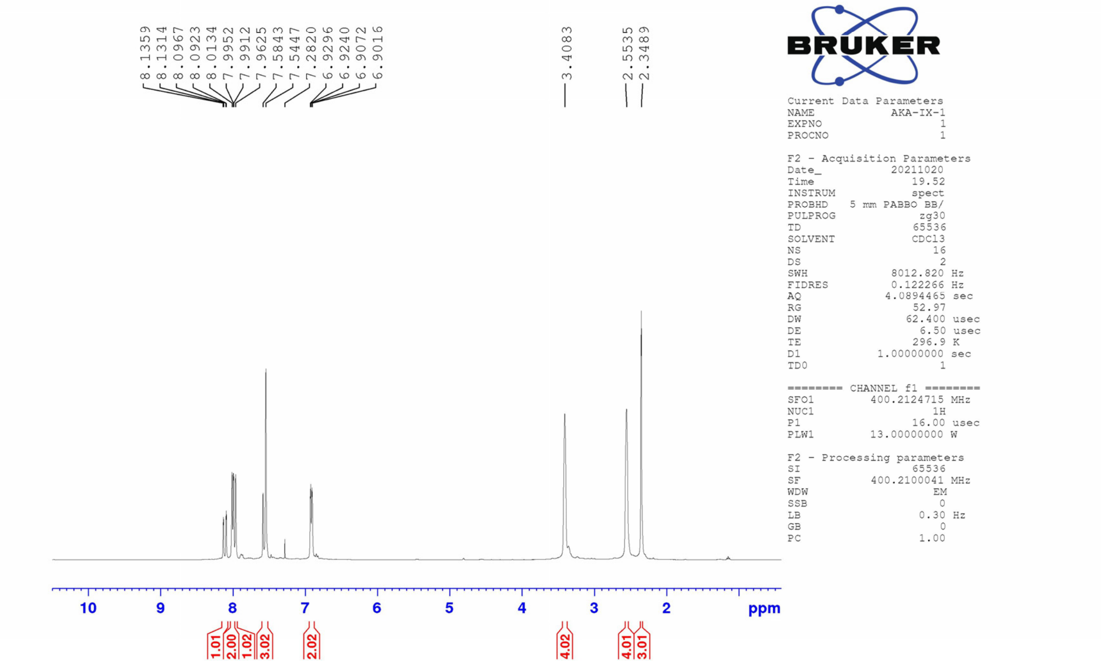

8.1359
8.1314
8.0967
8.0923
8.0134
7.9952
7.9912
7.9625
7.5843
7.5447
7.2820
6.9296
6.9240
6.9072
6.9016
3.4083
2.5535
2.3489
BRUKER
Current Data Parameters
NAME AKA-IX-1
EXPNO 1
PROCNO 1
F2 - Acquisition Parameters
Date_ 20211020
Time 19.52
INSTRUM spect
PROBHD 5 mm PABBO BB/
PULPROG zg30
TD 65536
SOLVENT CDCl3
NS 16
DS 2
SWH 8012.820 Hz
FIDRES 0.122266 Hz
AQ 4.0894465 sec
RG 52.97
DW 62.400 usec
DE 6.50 usec
TE 296.9 K
D1 1.00000000 sec
TD0 1
======== CHANNEL f1 ========
SFO1 400.2124715 MHz
NUC1 1H
P1 16.00 usec
PLW1 13.00000000 W
F2 - Processing parameters
SI 65536
SF 400.2100041 MHz
WDW EM
SSB 0
LB 0.30 Hz
GB 0
PC 1.00
10
9
8
7
6
5
4
3
2
ppm
1.01
2.00
1.02
3.02
2.02
4.02
4.01
3.01

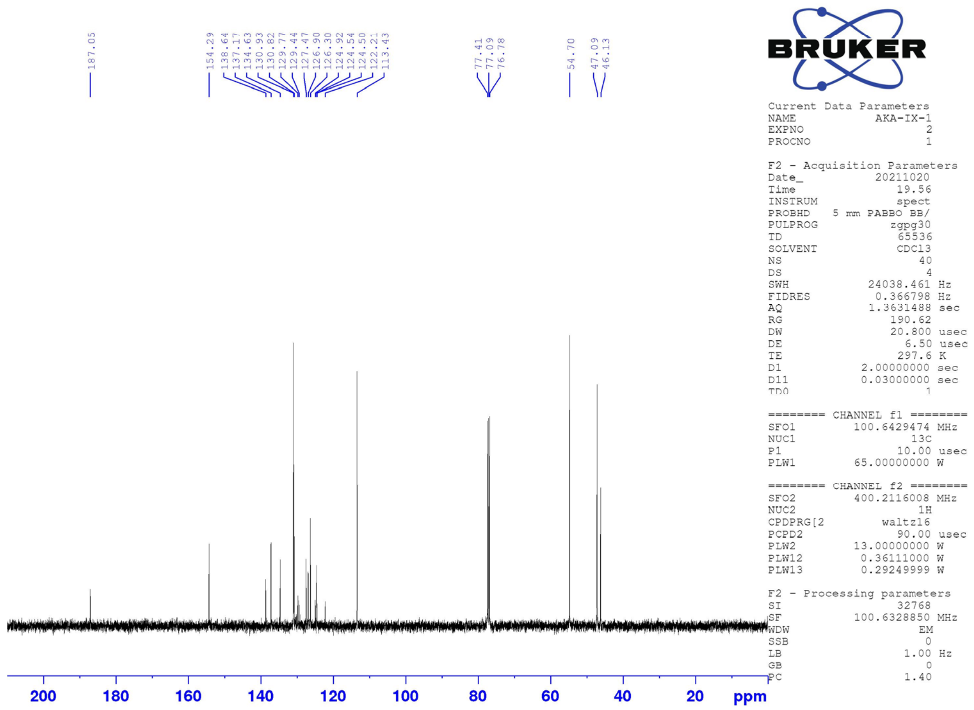

187.05
154.29
138.64
137.17
134.63
130.93
130.82
129.77
129.44
127.47
126.90
126.30
124.92
124.54
124.50
122.21
113.43
77.41
77.09
76.78
54.70
47.09
46.13
BRUKER
Current Data Parameters
NAME AKA-IX-1
EXPNO 2
PROCNO 1
F2 - Acquisition Parameters
Date_ 20211020
Time 19.56
INSTRUM spect
PROBHD 5 mm PABBO BB/
PULPROG zgpg30
TD 65536
SOLVENT CDCl3
NS 40
DS 4
SWH 24038.461 Hz
FIDRES 0.366798 Hz
AQ 1.3631488 sec
RG 190.62
DW 20.800 usec
DE 6.50 usec
TE 297.6 K
D1 2.00000000 sec
D11 0.03000000 sec
TD0 1
======== CHANNEL f1 ========
SFO1 100.6429474 MHz
NUC1 13C
P1 10.00 usec
PLW1 65.00000000 W
======== CHANNEL f2 ========
SFO2 400.2116008 MHz
NUC2 1H
CPDPRG[2 waltz16
PCPD2 90.00 usec
PLW2 13.00000000 W
PLW12 0.36111000 W
PLW13 0.29249999 W
F2 - Processing parameters
SI 32768
SF 100.6328850 MHz
WDW EM
SSB 0
LB 1.00 Hz
GB 0
PC 1.40
200
180
160
140
120
100
80
60
40
20
ppm

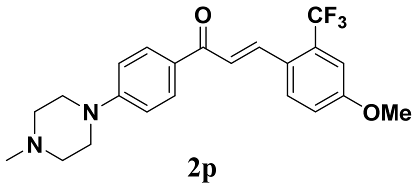
O
CF3
N
N
OMe

2p

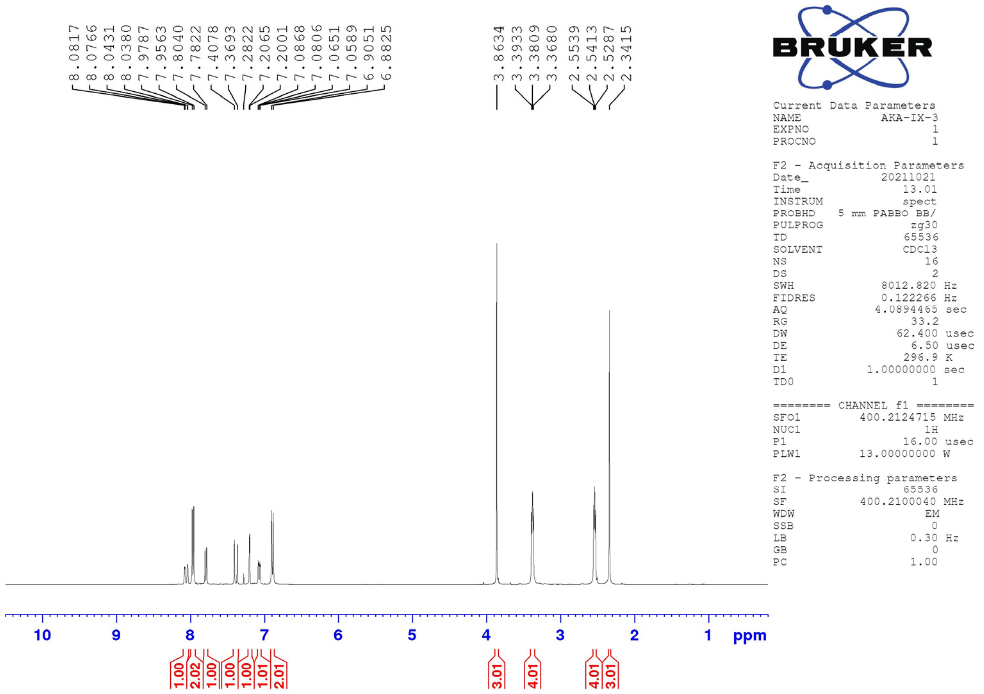
8.0817
8.0766
8.0431
8.0380
7.9787
7.9563
7.8040
7.7822
7.4078
7.3693
7.2822
7.2065
7.2001
7.0868
7.0806
7.0651
7.0589
6.9051
6.8825
3.8634
3.3933
3.3809
3.3680
2.5539
2.5413
2.5287
2.3415
1.00
2.02
1.00
1.00
1.00
1.01
2.01
3.01
4.01
4.01
3.01
ppm
BRUKER
Current Data Parameters
NAME AKA-IX-3
EXPNO 1
PROCNO 1
F2 - Acquisition Parameters
Date_ 20211021
Time 13.01
INSTRUM spect
PROBHD 5 mm PABBO BB/
PULPROG zg30
TD 65536
SOLVENT CDCl3
NS 16
DS 2
SWH 8012.820 Hz
FIDRES 0.122266 Hz
AQ 4.0894465 sec
RG 33.2
DW 62.400 usec
DE 6.50 usec
TE 296.9 K
D1 1.00000000 sec
TD0 1
======== CHANNEL f1 ========
SFO1 400.2124715 MHz
NUC1 1H
P1 16.00 usec
PLW1 13.00000000 W
F2 - Processing parameters
SI 65536
SF 400.2100040 MHz
WDW EM
SSB 0
LB 0.30 Hz
GB 0
PC 1.00

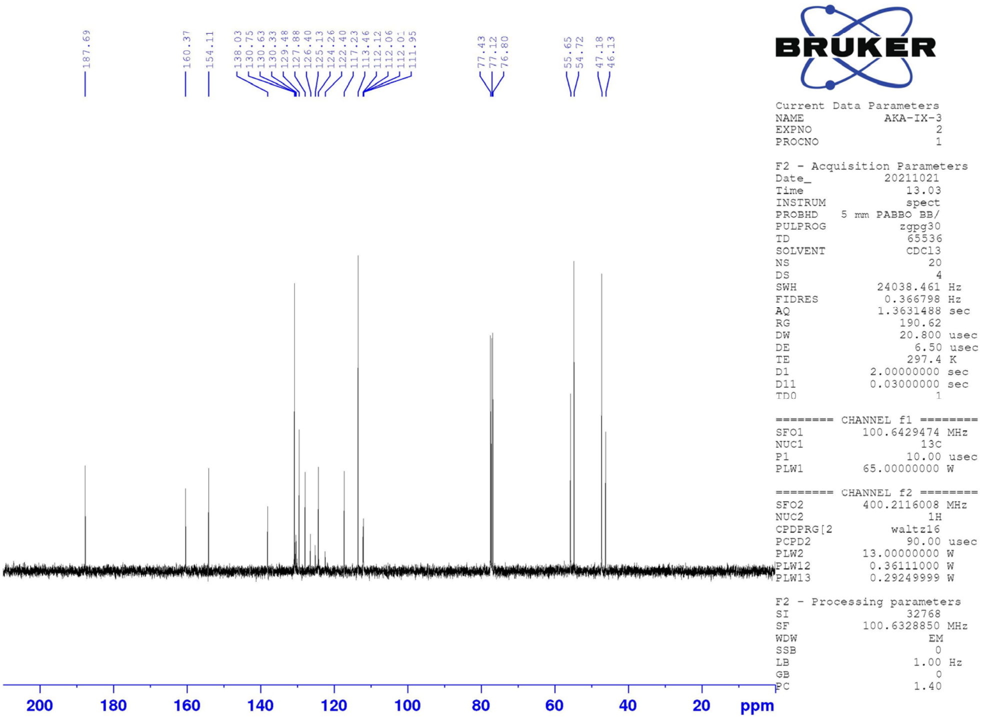
187.69
160.37
154.11
138.03
130.75
130.63
130.33
129.48
127.88
126.40
125.13
124.26
122.40
117.23
113.46
112.12
112.06
111.95
77.43
77.12
76.80
55.65
54.72
47.18
46.13
ppm
BRUKER
Current Data Parameters
NAME AKA-IX-3
EXPNO 2
PROCNO 1
F2 - Acquisition Parameters
Date_ 20211021
Time 13.03
INSTRUM spect
PROBHD 5 mm PABBO BB/
PULPROG zgpg30
TD 65536
SOLVENT CDCl3
NS 20
DS 4
SWH 24038.461 Hz
FIDRES 0.366798 Hz
AQ 1.3631488 sec
RG 190.62
DW 20.800 usec
DE 6.50 usec
TE 297.4 K
D1 2.00000000 sec
D11 0.03000000 sec
TD0 1
======== CHANNEL f1 ========
SFO1 100.6429474 MHz
NUC1 13C
P1 10.00 usec
PLW1 65.00000000 W
======== CHANNEL f2 ========
SFO2 400.2116008 MHz
NUC2 1H
CPDPRG[2 waltz16
PCPD2 90.00 usec
PLW2 13.00000000 W
PLW12 0.36111000 W
PLW13 0.29249999 W
F2 - Processing parameters
SI 32768
SF 100.6328850 MHz
WDW EM
SSB 0
LB 1.00 Hz
GB 0
PC 1.40

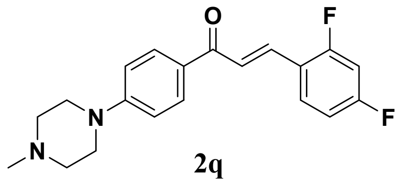

2q

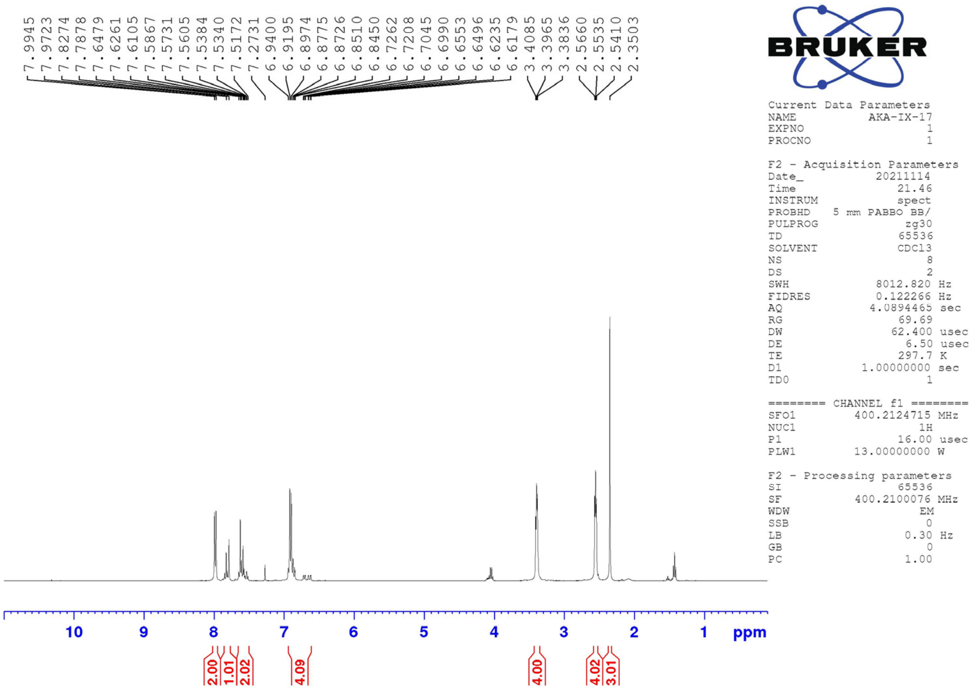
7.9945
7.9723
7.8274
7.7878
7.6479
7.6261
7.6105
7.5867
7.5731
7.5605
7.5384
7.5340
7.5172
7.2731
6.9400
6.9195
6.8974
6.8775
6.8726
6.8510
6.8450
6.7262
6.7208
6.7045
6.6990
6.6553
6.6496
6.6235
6.6179
3.4085
3.3965
3.3836
2.5660
2.5535
2.5410
2.3503
BRUKER
Current Data Parameters
NAME AKA-IX-17
EXPNO 1
PROCNO 1
F2 - Acquisition Parameters
Date_ 20211114
Time 21.46
INSTRUM spect
PROBHD 5 mm PABBO BB/
PULPROG zg30
TD 65536
SOLVENT CDCl3
NS 8
DS 2
SWH 8012.820 Hz
FIDRES 0.122266 Hz
AQ 4.0894465 sec
RG 69.69
DW 62.400 usec
DE 6.50 usec
TE 297.7 K
D1 1.00000000 sec
TD0 1
======== CHANNEL f1 ========
SFO1 400.2124715 MHz
NUC1 1H
P1 16.00 usec
PLW1 13.00000000 W
F2 - Processing parameters
SI 65536
SF 400.2100076 MHz
WDW EM
SSB 0
LB 0.30 Hz
GB 0
PC 1.00
10 9 8 7 6 5 4 3 2 1 ppm
2.00
1.01
2.02
4.09
4.00
4.02
3.01

Figure S2. HRMS charts

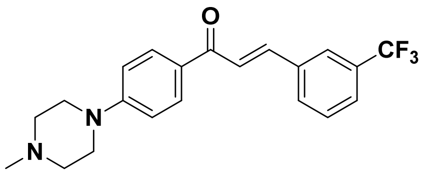

2a

$C_{21}H_{22}F_3N_2O$ $[M+H]^+$:

Calculated: 375.1684

Found: 375.1701

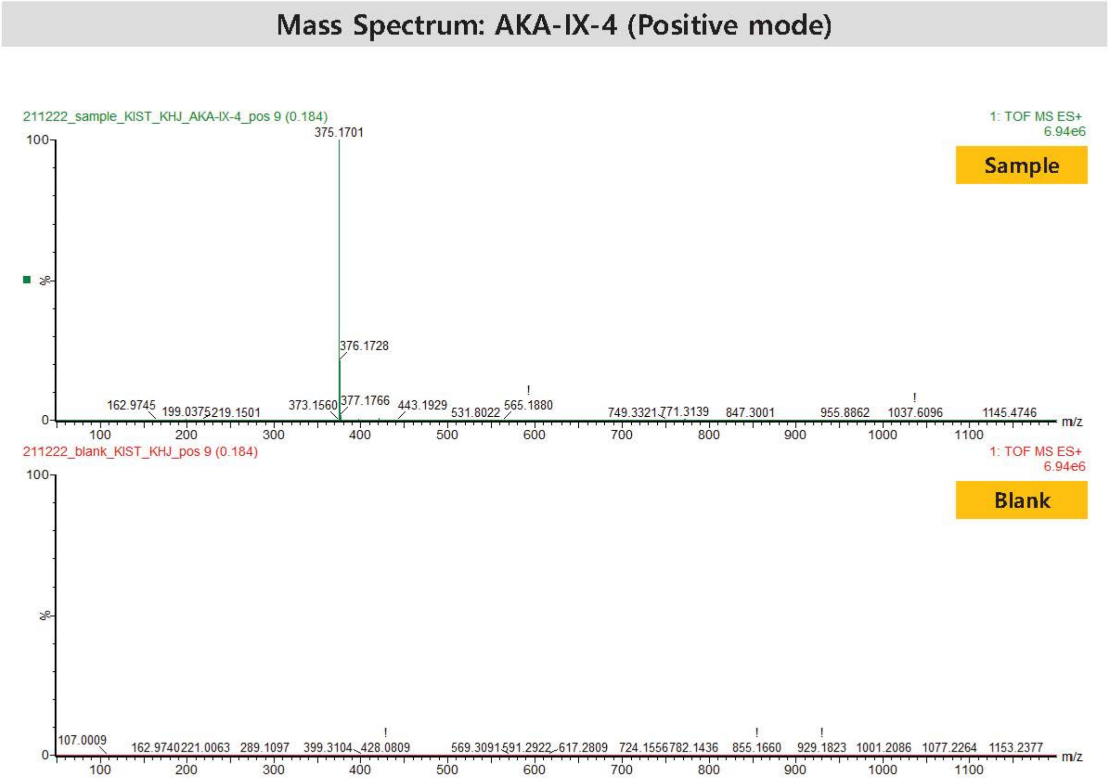

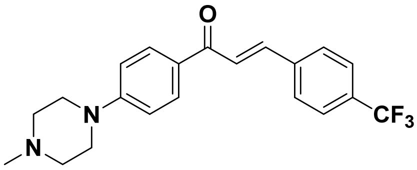

2b

$C_{21}H_{22}F_3N_2O$ $[M+H]^+$:

Calculated: 375.1684

Found: 375.1693

Mass Spectrum: AKA-IX-20 (Positive mode)

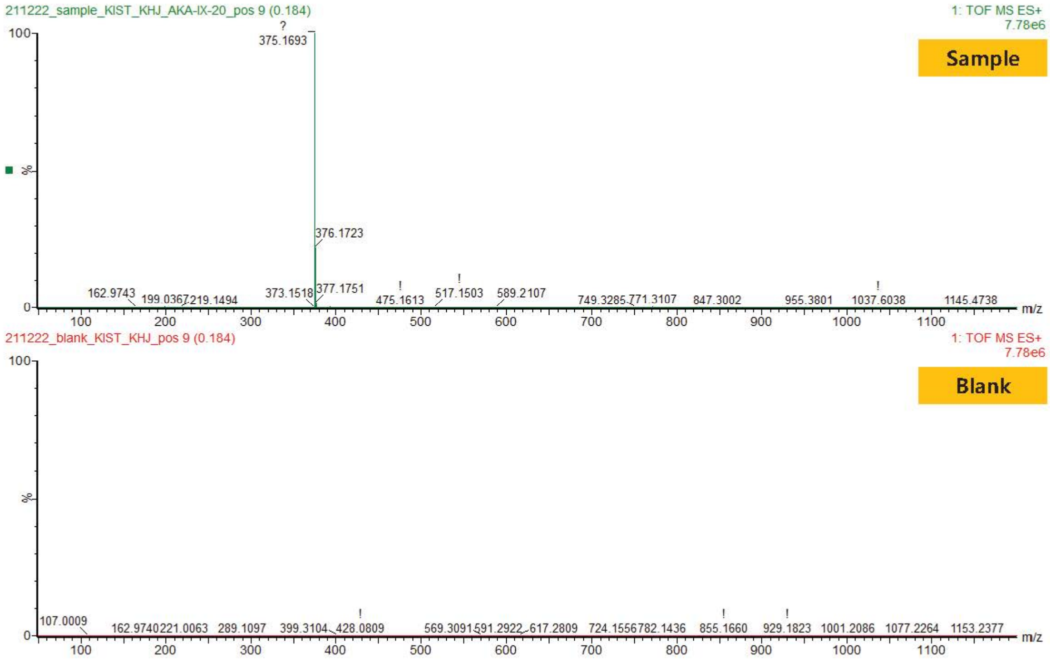

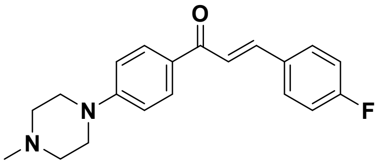

2c

$C_{20}H_{22}FN_2O$ $[M+H]^+$:

Calculated: 325.1716

Found: 325.1722

Mass Spectrum: AKA-IX-19 (Positive mode)

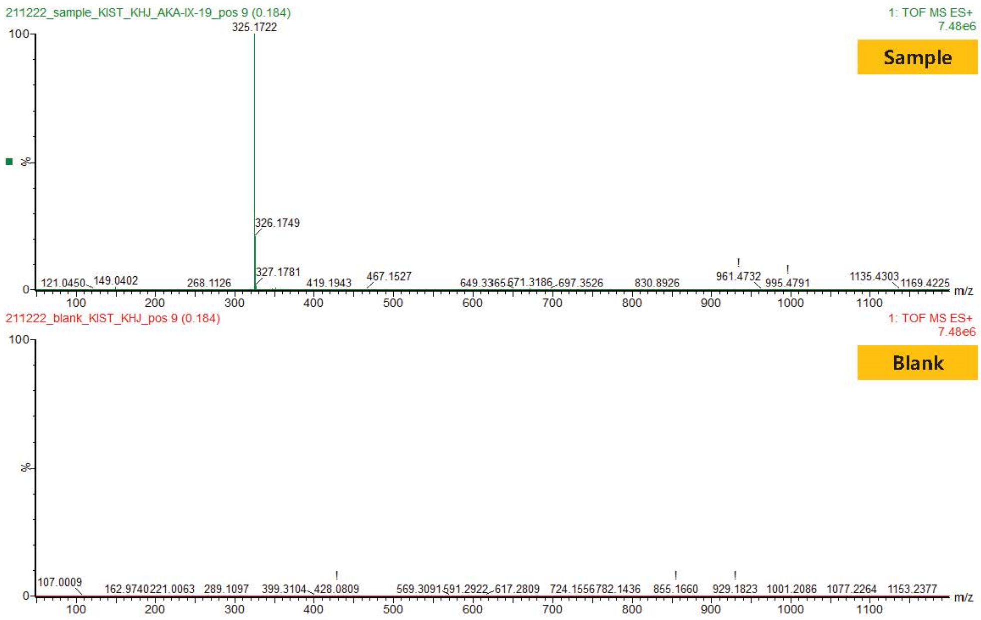

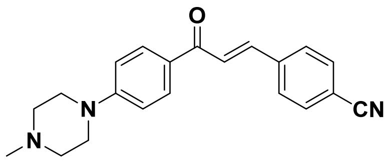

2d

$C_{21}H_{22}N_3O$ $[M+H]^+$:

Calculated: 332.1763

Found: 332.1774

Mass Spectrum: AK-VIII-91 (Positive mode)

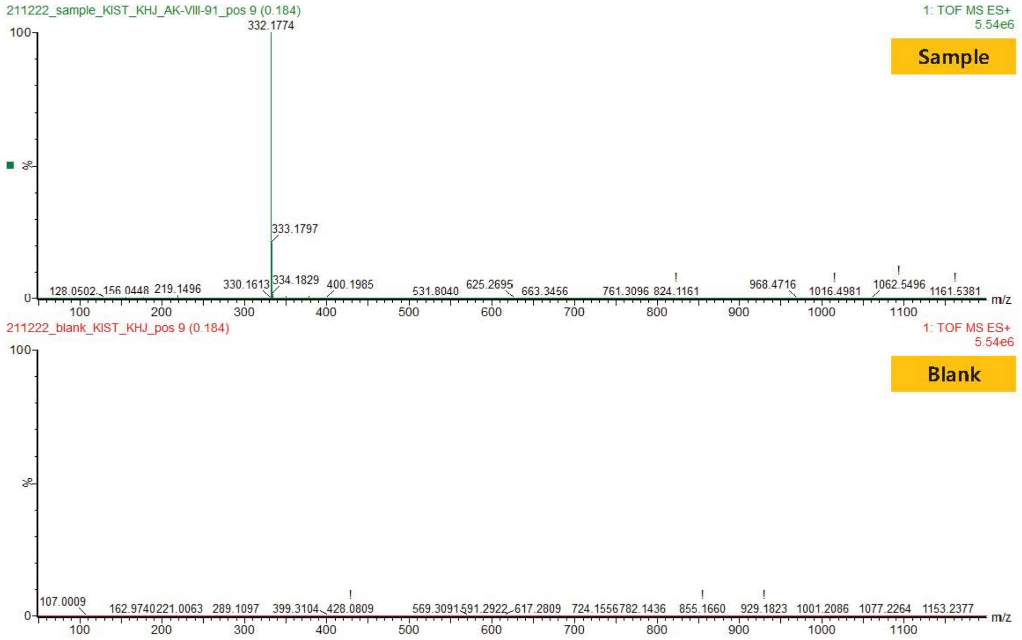

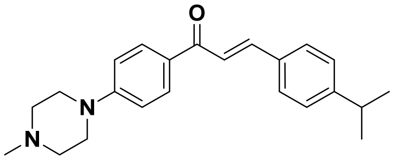

2e

$C_{23}H_{29}N_2O$ $[M+H]^+$:

Calculated: 349.2280

Found: 349.2291

Mass Spectrum: AK-VIII-92 (Positive mode)

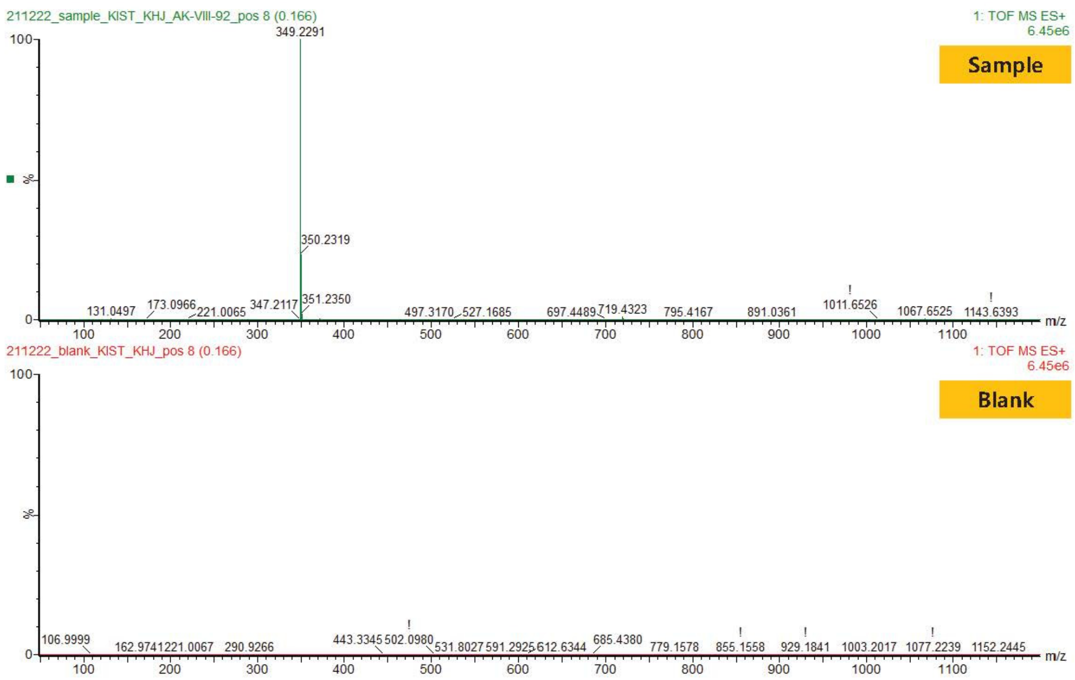

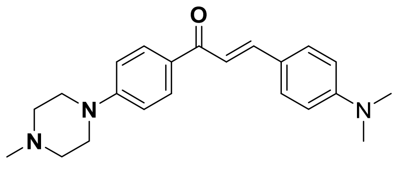

2f

$C_{22}H_{28}N_3O$ $[M+H]^+$:

Calculated: 350.2232

Found: 350.2239

Mass Spectrum: AK-VIII-100 (Positive mode)

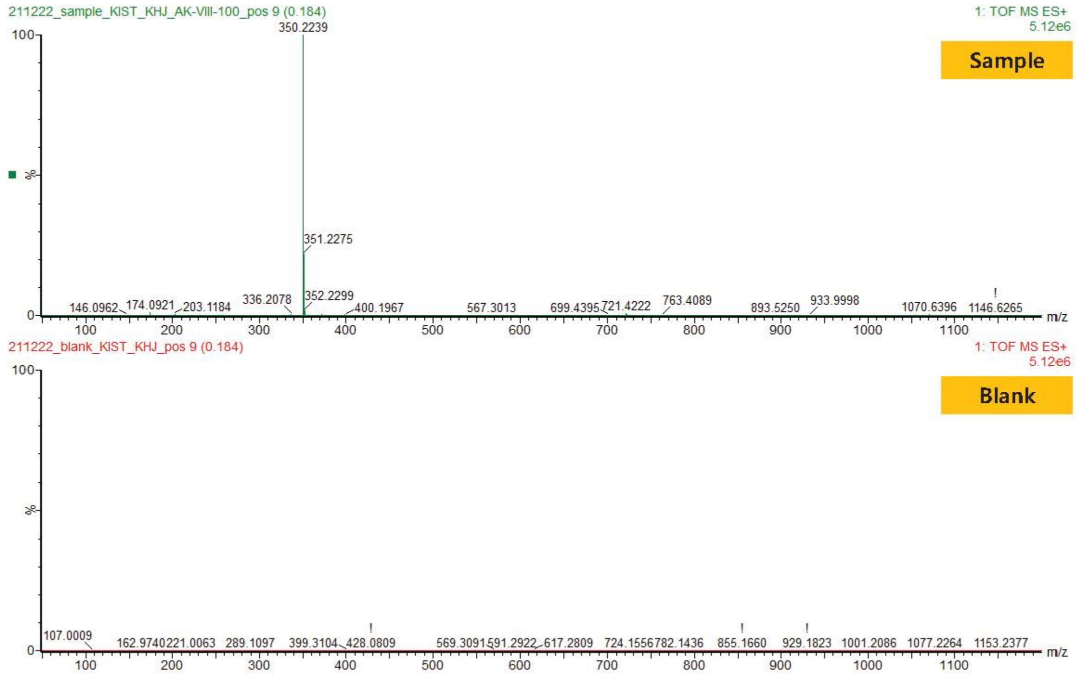

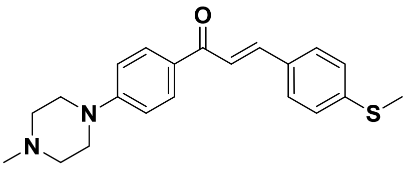

2g

$C_{21}H_{25}N_2OS$ $[M+H]^+$:

Calculated: 353.1688

Found: 353.1694

Mass Spectrum: AK-VIII-93 (Positive mode)

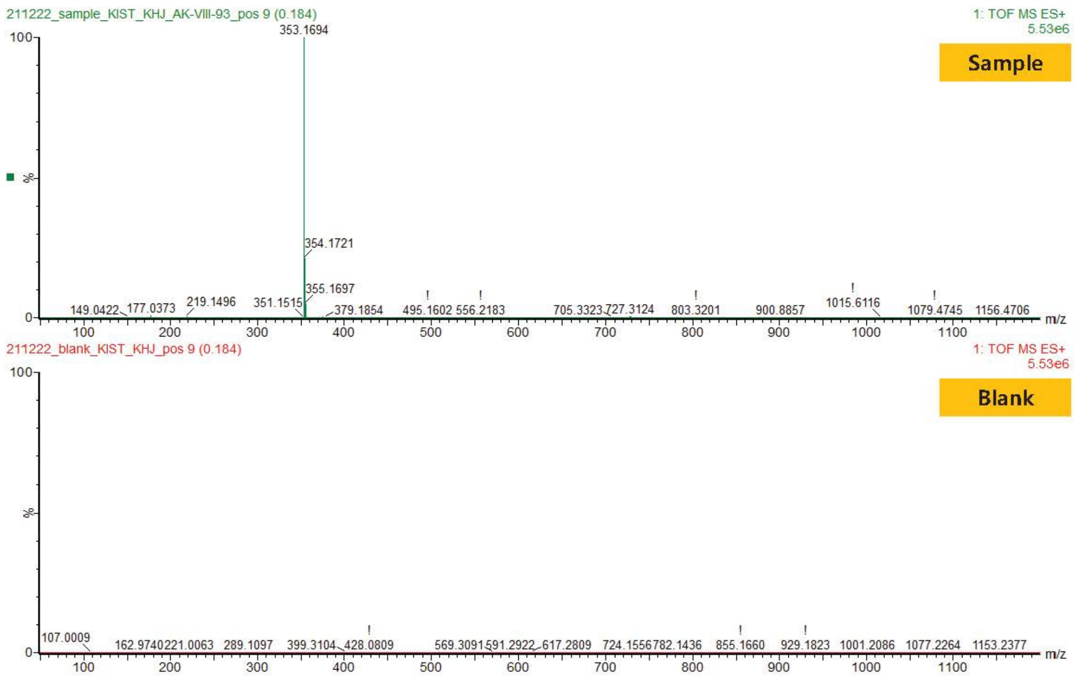

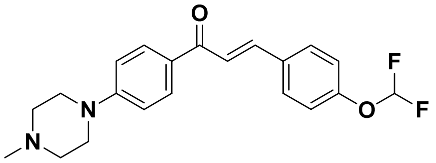

2h

$C_{21}H_{23}F_2N_2O_2$ $[M+H]^+$:

Calculated: 373.1728

Found: 373.1732

Mass Spectrum: AKA-IX-2 (Positive mode)

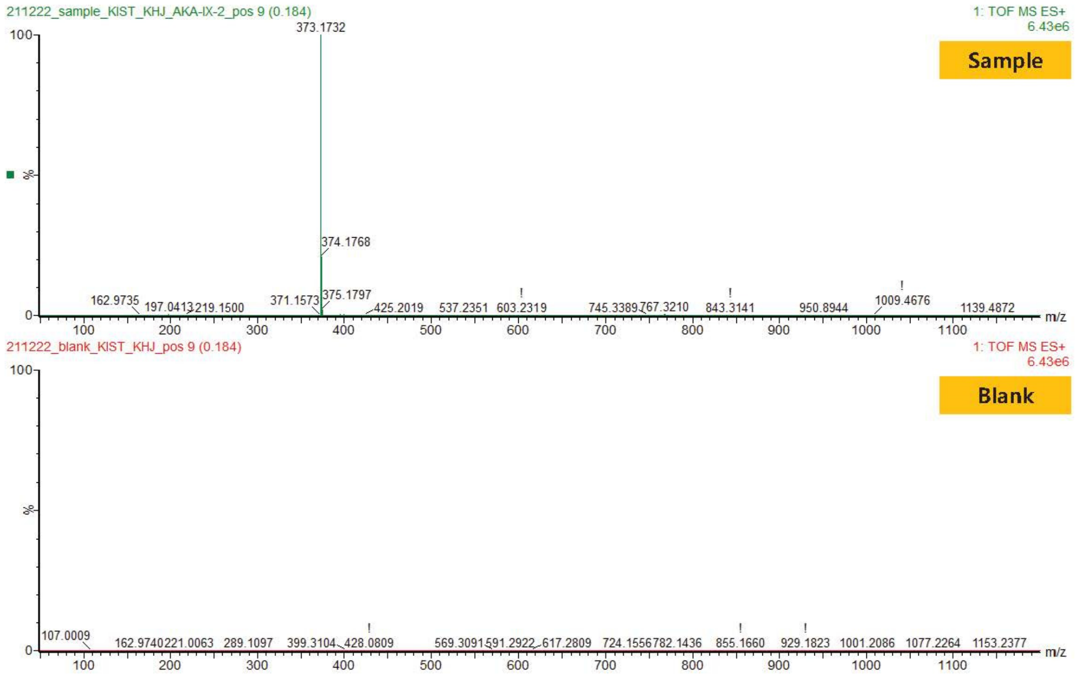

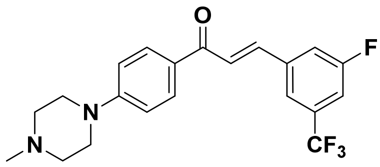

2i

$C_{21}H_{21}F_4N_2O$ $[M+H]^+$:

Calculated: 393.1590

Found: 393.1592

Mass Spectrum: AK-VIII-23 (Positive mode)

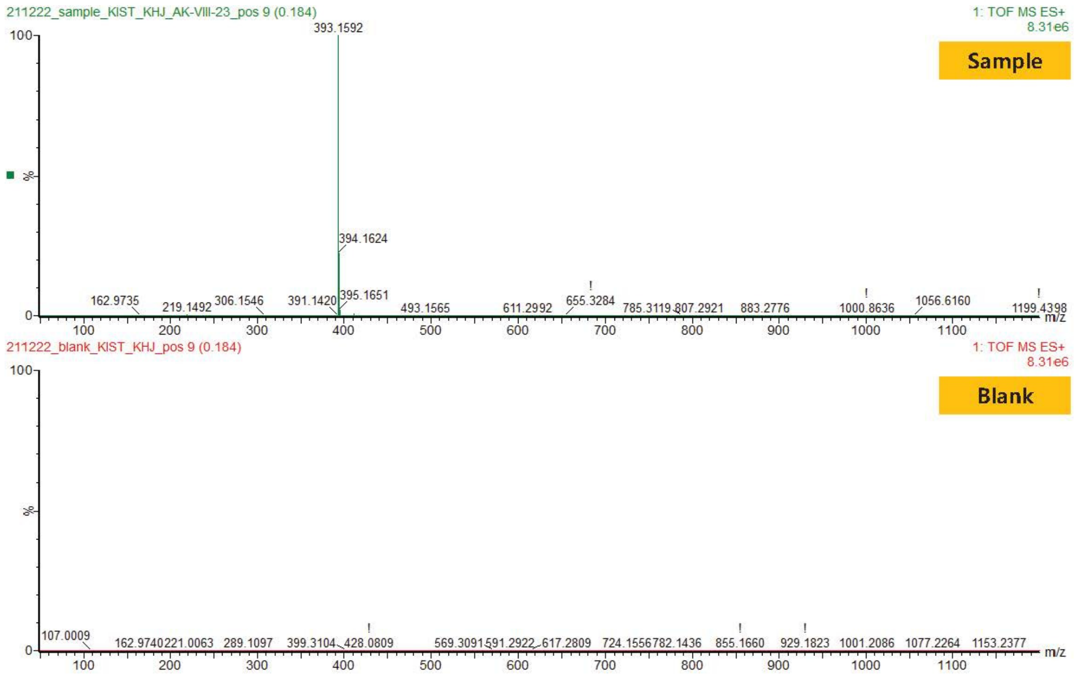

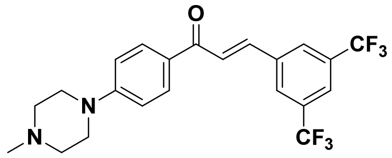

2j

$C_{22}H_{21}F_6N_2O$ $[M+H]^+$:

Calculated: 443.1558

Found: 443.1561

Mass Spectrum: AK-VIII-25 (Positive mode)

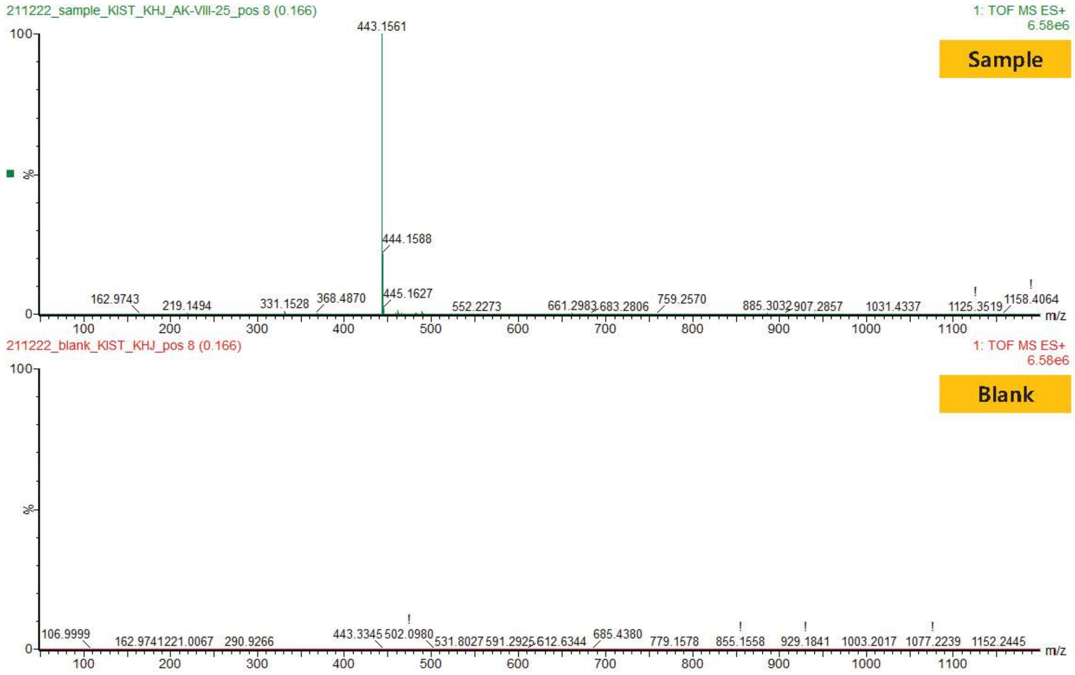

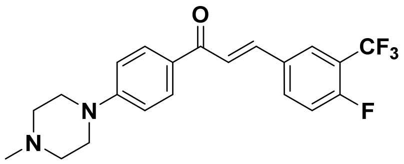

2k

$C_{21}H_{21}F_4N_2O$ $[M+H]^+$:

Calculated: 393.1590

Found: 393.1595

Mass Spectrum: AK-VIII-27 (Positive mode)

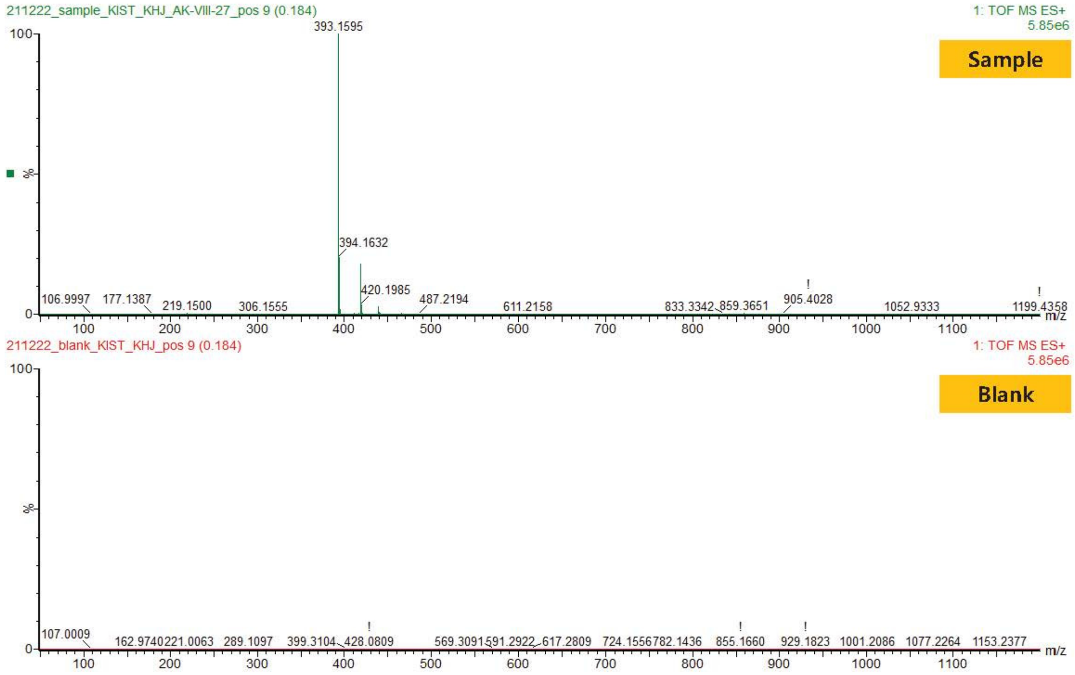

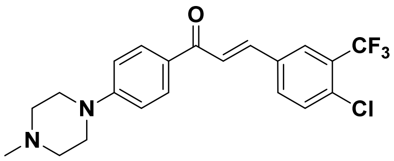

2l

$C_{21}H_{21}ClF_3N_2O$ $[M+H]^+$:

Calculated: 409.1295

Found: 409.1303

Mass Spectrum: AK-VIII-28 (Positive mode)

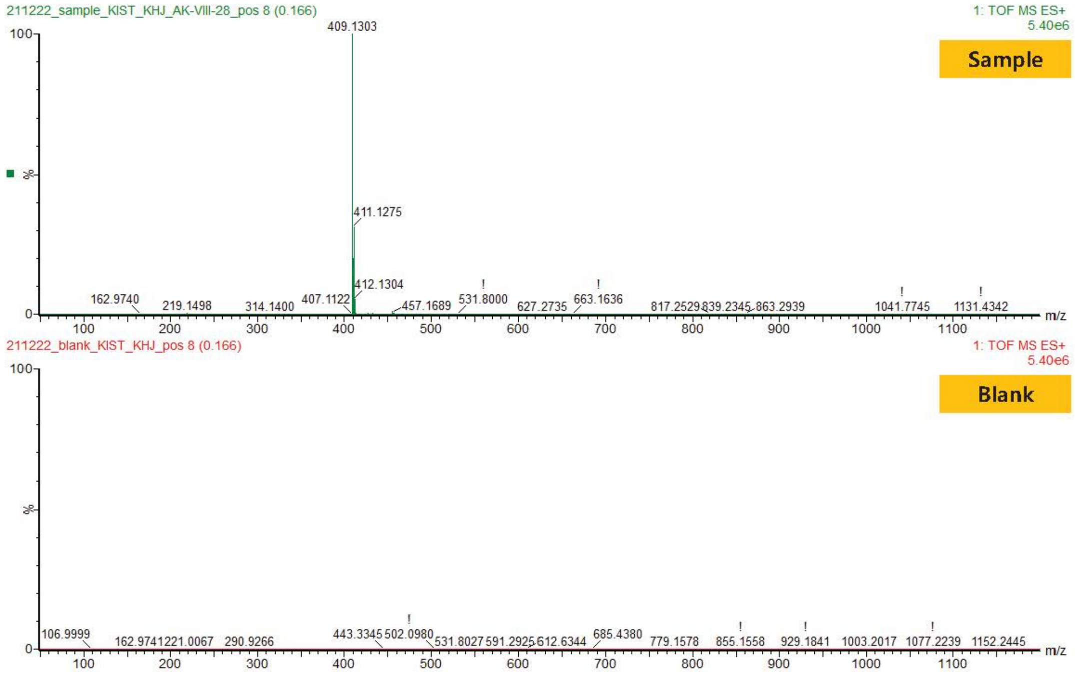

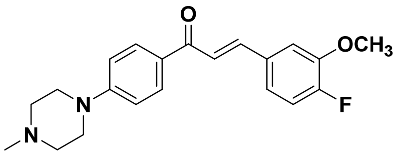

2m

$C_{21}H_{24}FN_2O_2$ $[M+H]^+$:

Calculated: 355.1822

Found: 355.1826

Mass Spectrum: AK-VIII-30 (Positive mode)

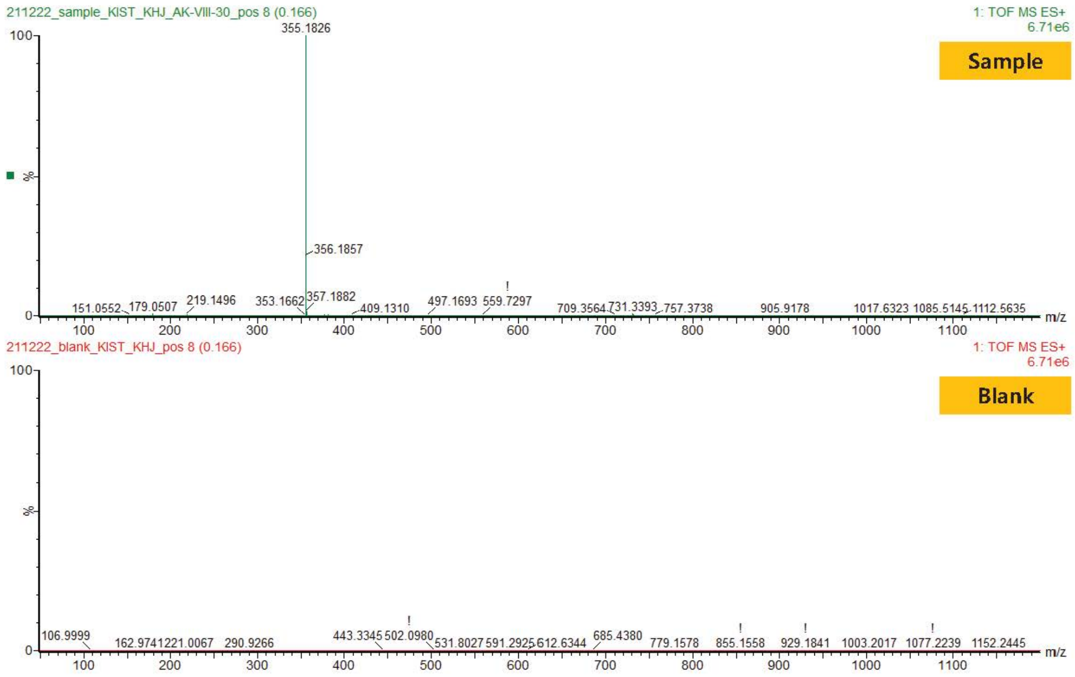

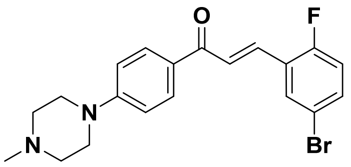

2n

$C_{20}H_{21}BrFN_2O$ $[M+H]^+$:

Calculated: 403.0821

Found: 403.0836

Mass Spectrum: AK-VIII-94 (Positive mode)

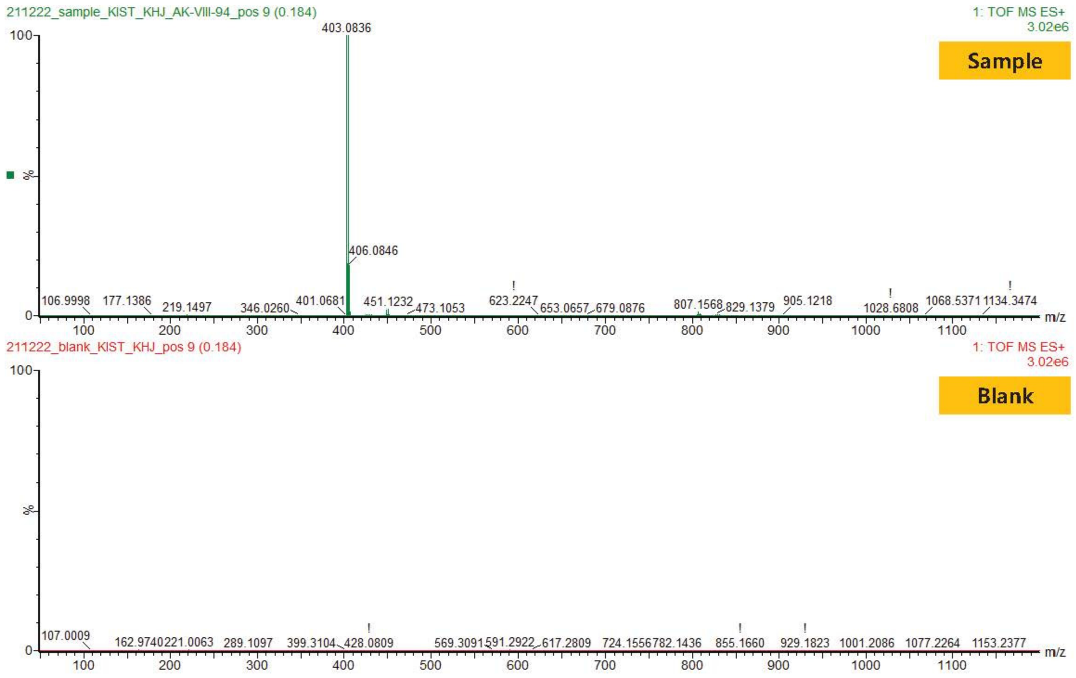

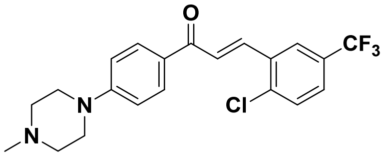

2o

$C_{21}H_{21}ClF_3N_2O$ $[M+H]^+$:

Calculated: 409.1295

Found: 409.1295

Mass Spectrum: AKA-IX-1 (Positive mode)

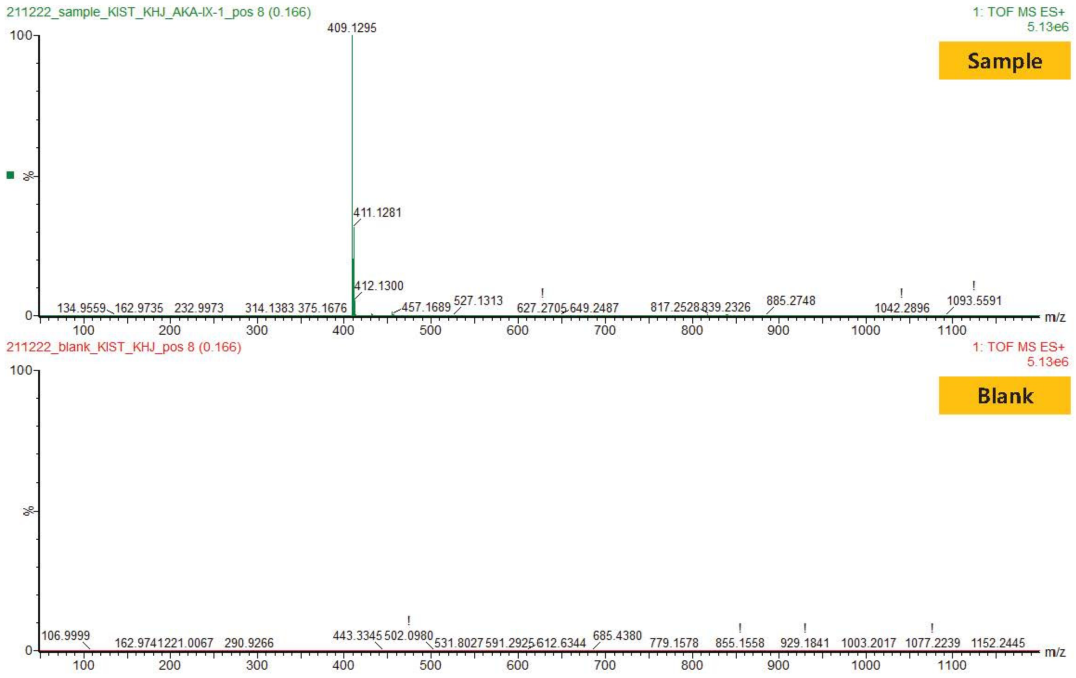

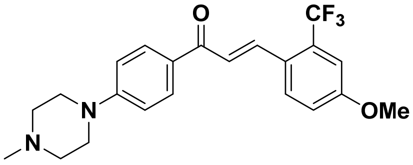

2p

$C_{22}H_{24}F_3N_2O_2$ $[M+H]^+$:

Calculated: 405.1790

Found: 405.1800

Mass Spectrum: AKA-IX-3 (Positive mode)

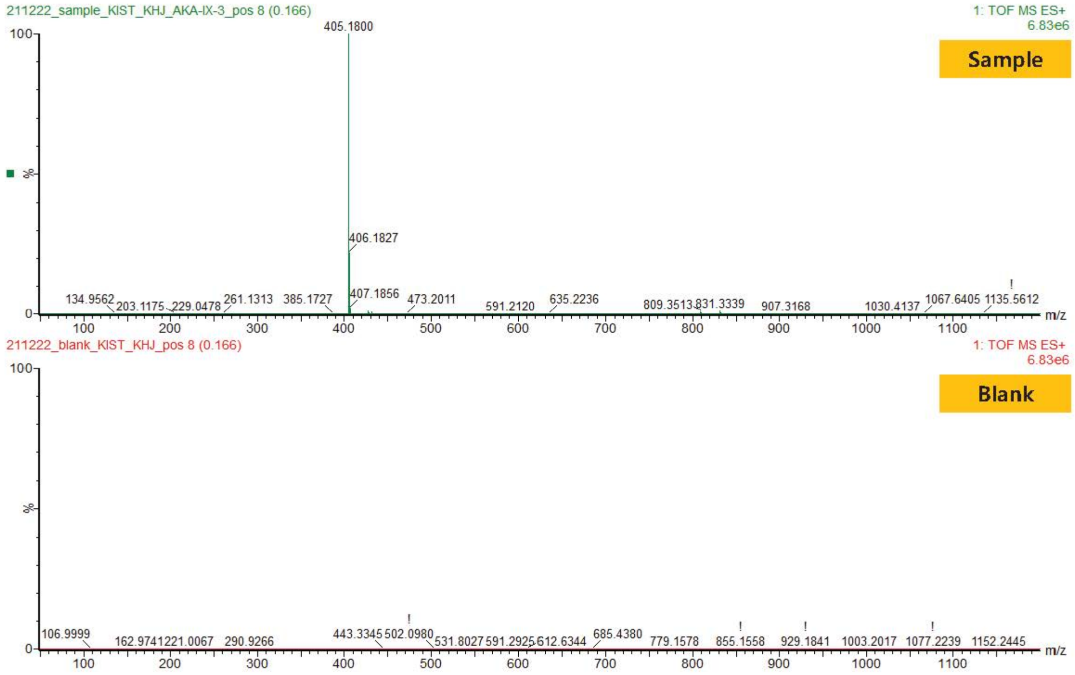

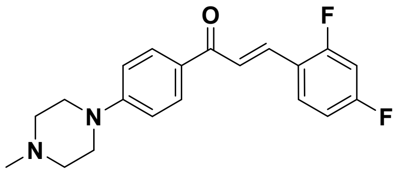

2q

$C_{20}H_{21}F_2N_2O$ $[M+H]^+$:

Calculated: 343.1622

Found: 343.1622

Mass Spectrum: AKA-IX-17 (Positive mode)

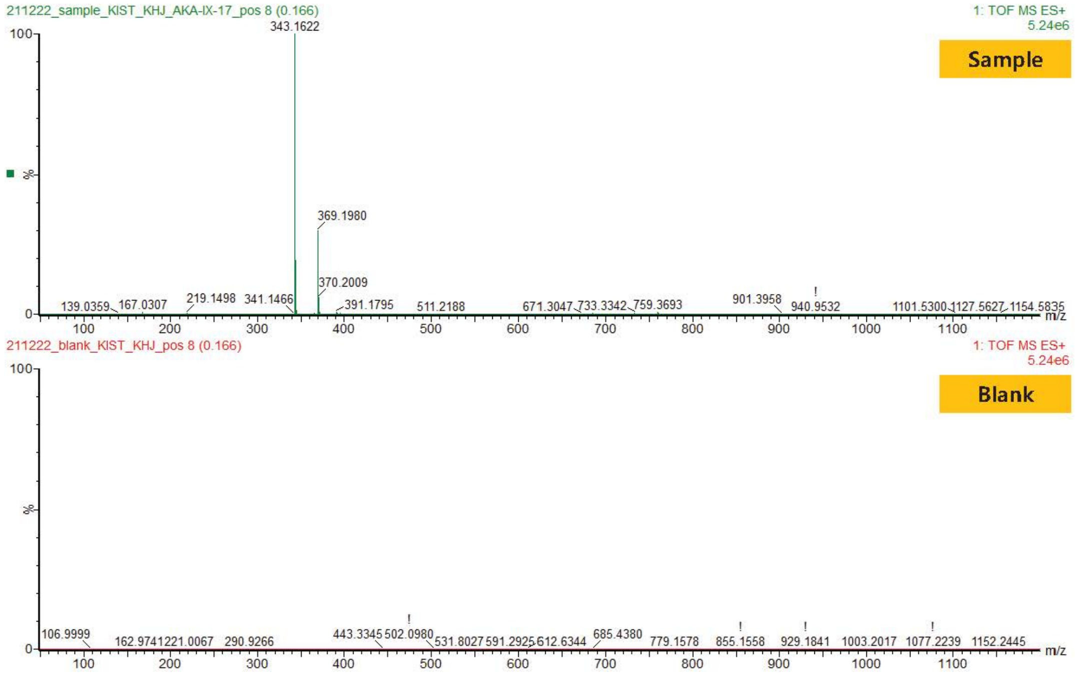

Figure S3. IR spectra

2a

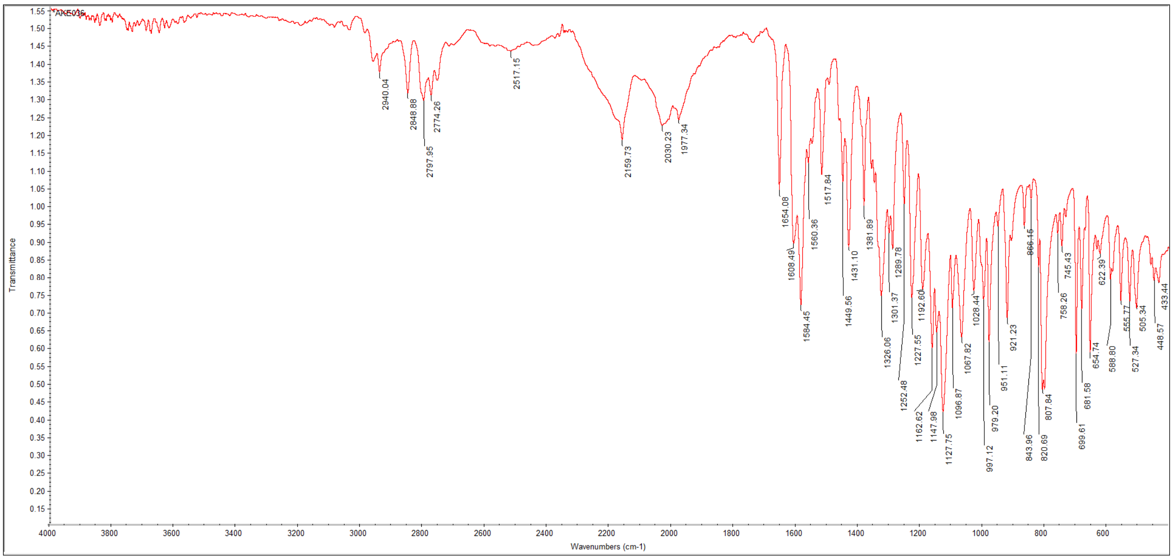

2b

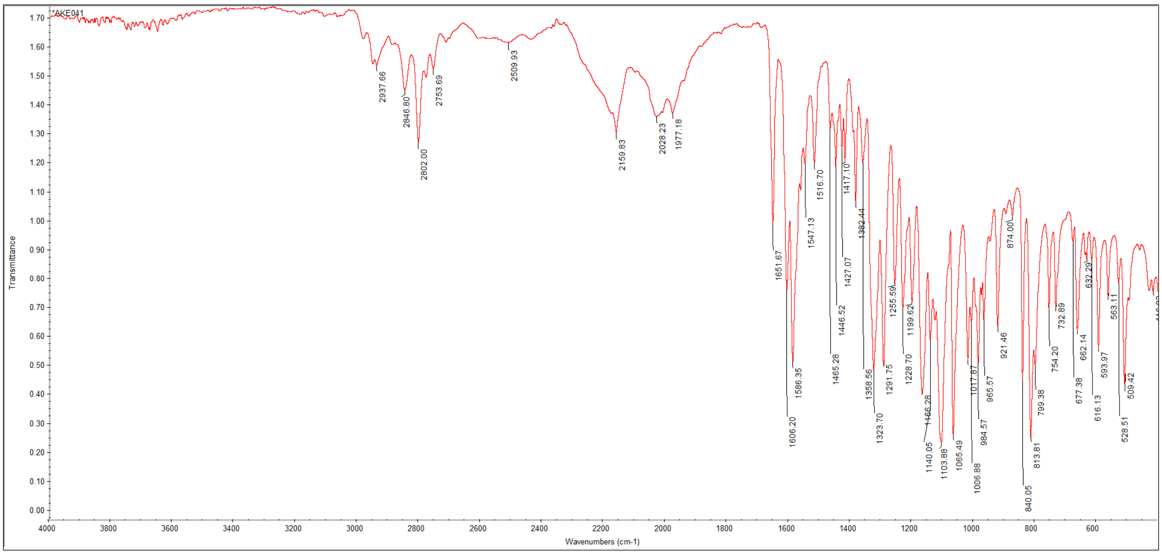

2c

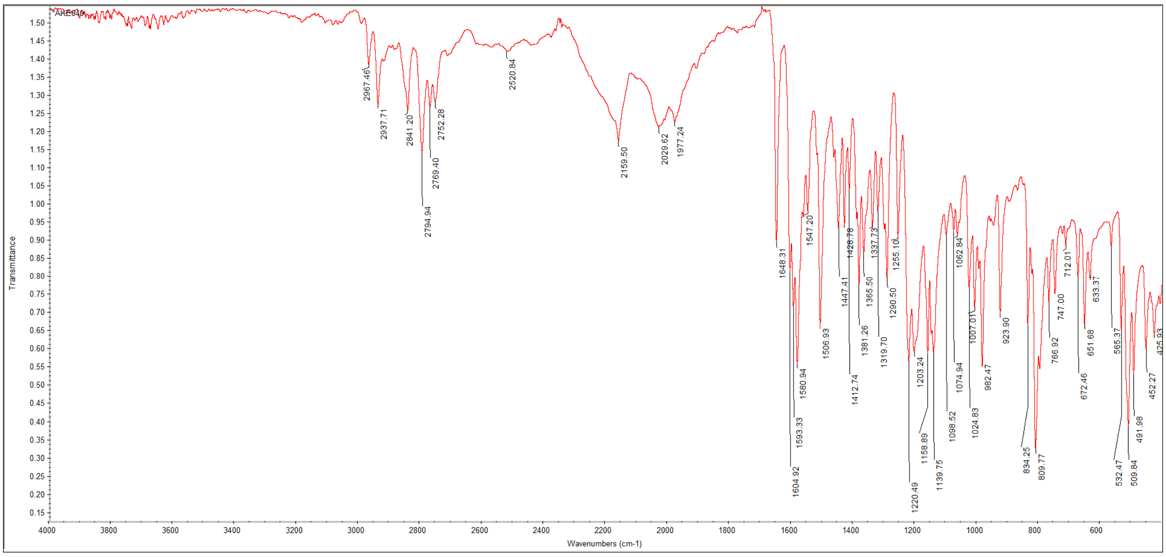
Transmittance
Wavenumbers (cm-1)
2967.46
2937.71
2841.20
2794.94
2769.40
2752.28
2520.84
2159.50
2029.62
1977.24
1648.31
1604.92
1593.33
1580.94
1547.20
1506.93
1447.41
1428.78
1412.74
1381.26
1365.50
1337.73
1319.70
1290.50
1255.10
1220.49
1203.24
1158.89
1139.75
1098.52
1074.94
1062.84
1024.83
1007.01
982.47
923.90
834.25
809.77
766.92
747.00
712.01
672.46
651.68
633.37
565.37
532.47
509.84
491.98
452.27
425.93

2d

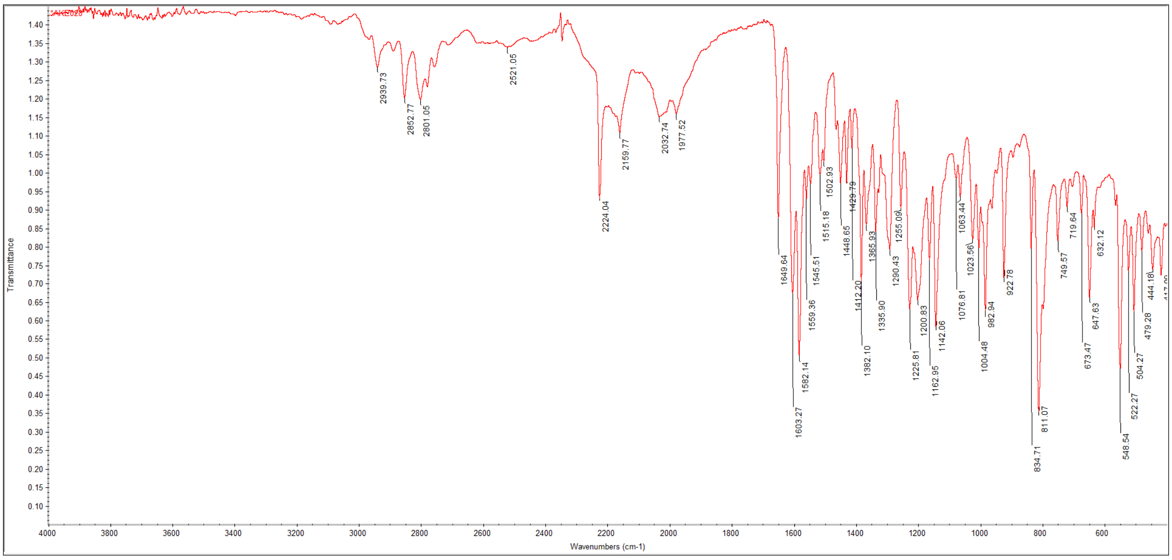
Transmittance
Wavenumbers (cm-1)
2939.73
2852.77
2801.05
2521.05
2224.04
2159.77
2032.74
1977.52
1649.64
1603.27
1582.14
1559.36
1545.51
1515.18
1502.93
1448.65
1429.79
1412.20
1382.10
1365.93
1335.90
1290.43
1255.09
1225.81
1200.83
1162.95
1142.06
1076.81
1063.44
1023.56
1004.48
982.94
922.78
834.71
811.07
749.57
719.64
673.47
647.63
632.12
548.54
522.27
504.27
479.28
444.18

2e

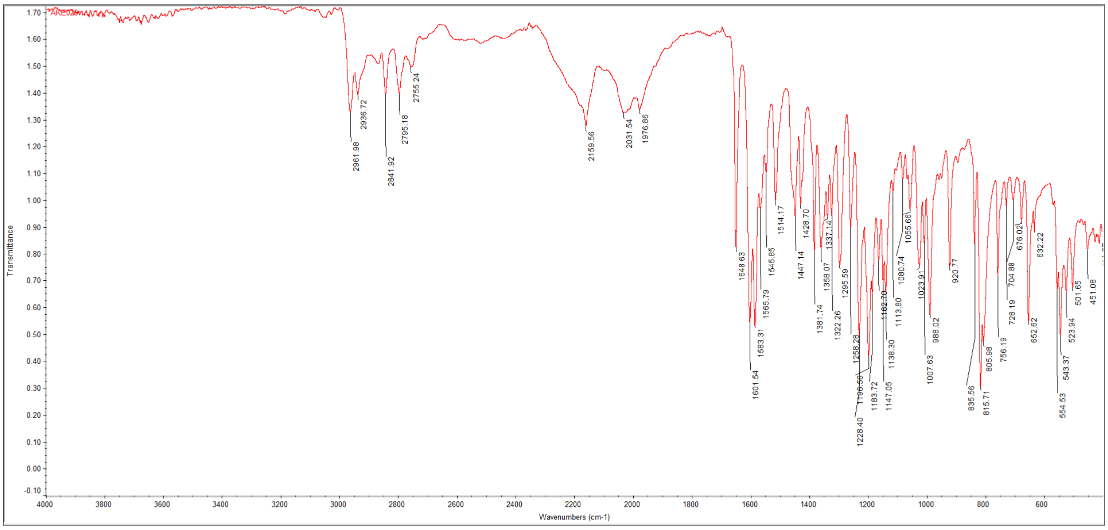
Transmittance
Wavenumbers (cm-1)
2961.98
2936.72
2841.92
2795.18
2755.24
2159.56
2031.54
1976.86
1648.63
1601.54
1583.31
1565.79
1545.85
1514.17
1447.14
1428.70
1381.74
1358.07
1337.14
1322.26
1295.59
1258.28
1228.40
1196.60
1183.72
1162.70
1147.05
1138.30
1113.80
1080.74
1055.66
1023.91
1007.63
988.02
920.77
835.56
815.71
805.98
756.19
728.19
704.88
676.02
652.62
632.22
554.63
543.37
523.94
501.65
451.08

2f

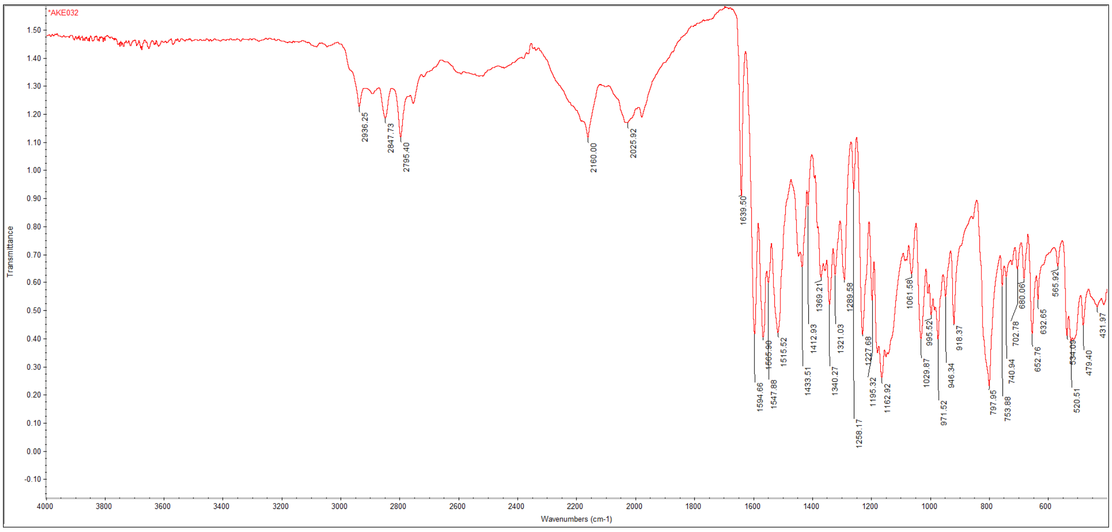
*AKE032
Transmittance
Wavenumbers (cm-1)
2936.25
2847.73
2795.40
2160.00
2025.92
1639.50
1594.66
1565.90
1547.88
1515.52
1433.51
1412.93
1369.21
1340.27
1321.03
1289.58
1258.17
1227.68
1195.32
1162.92
1061.58
1029.87
995.52
971.52
946.34
918.37
797.95
753.88
740.94
702.78
680.06
652.76
632.65
565.92
534.09
520.51
479.40
431.97

2g

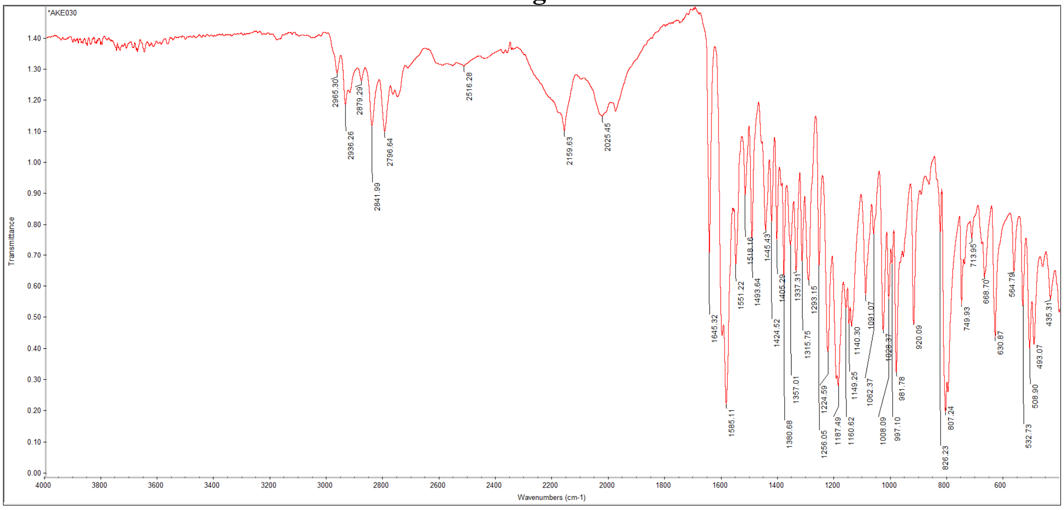
*AKE030
Transmittance
Wavenumbers (cm-1)
2965.30
2936.26
2879.29
2841.99
2796.64
2516.28
2159.63
2025.45
1645.92
1585.11
1551.22
1518.16
1493.64
1445.43
1424.52
1405.29
1380.68
1357.01
1337.31
1315.75
1293.15
1256.05
1224.59
1187.49
1160.62
1149.25
1140.30
1091.07
1062.37
1028.37
1008.09
997.10
981.78
920.09
826.23
807.24
749.93
713.95
668.70
630.87
564.79
532.73
508.90
493.07
435.31

2h

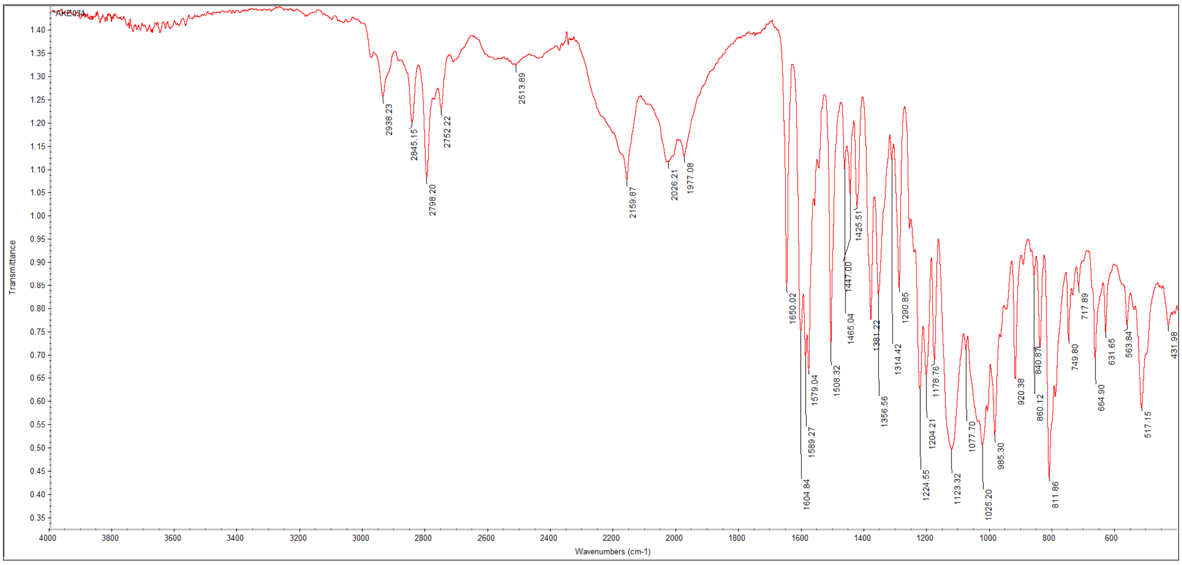
Transmittance
Wavenumbers (cm-1)
2938.23
2845.15
2798.20
2752.22
2513.89
2159.87
2026.21
1977.08
1650.02
1604.84
1589.27
1579.04
1508.32
1465.04
1447.00
1425.51
1381.22
1356.56
1314.42
1290.85
1224.55
1204.21
1178.76
1123.32
1077.70
1025.20
985.30
920.38
860.12
840.87
811.86
749.80
717.89
664.90
631.65
563.84
517.15
431.98

2i

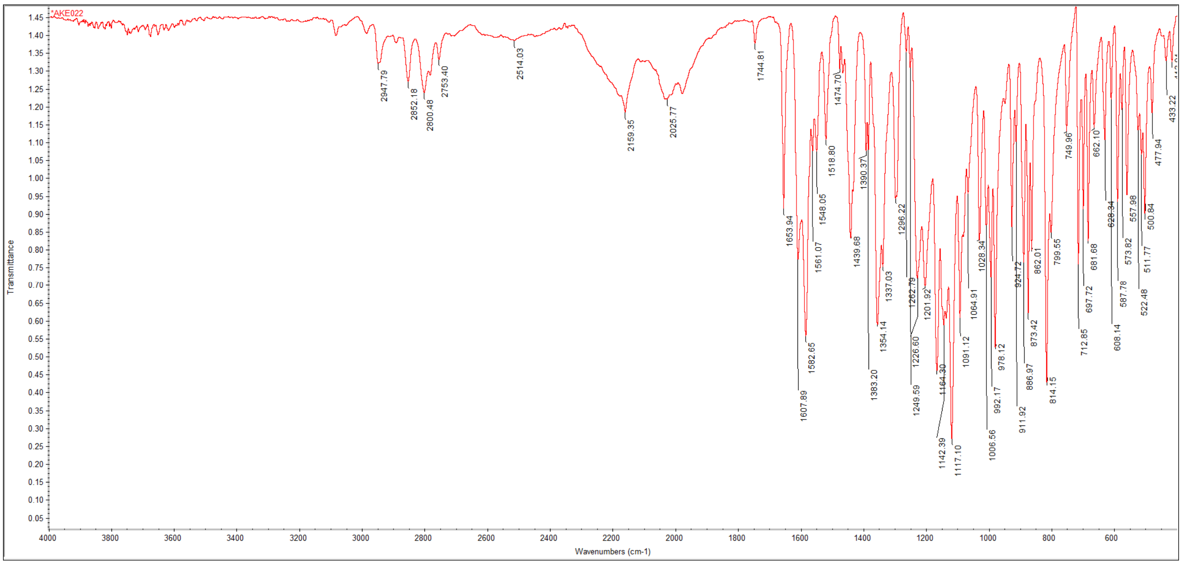
AKE022
Transmittance
Wavenumbers (cm-1)

2j

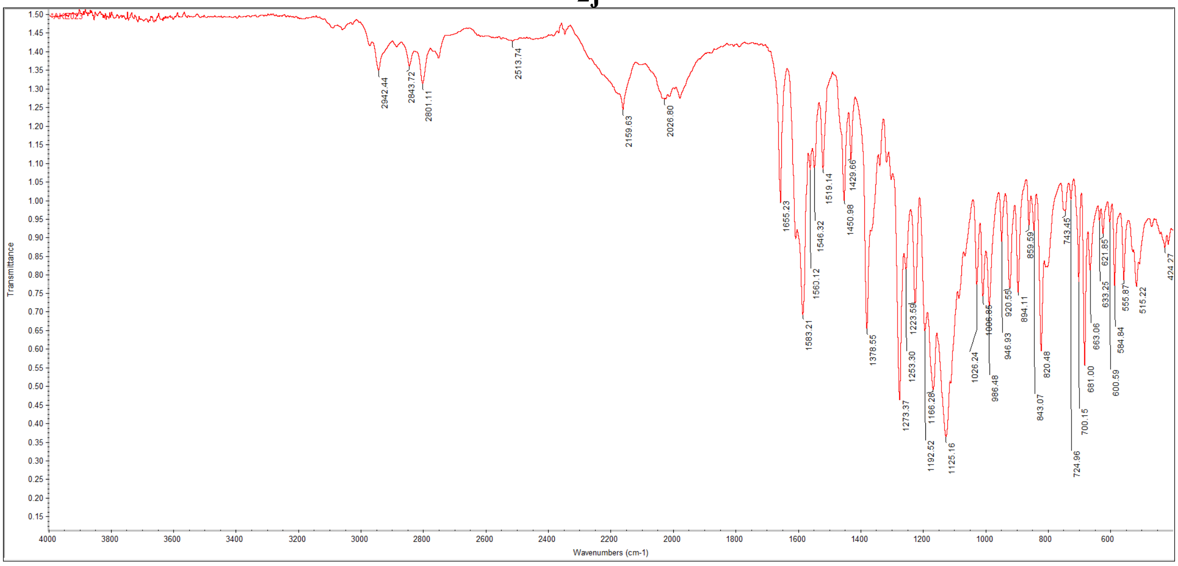
AKE023
Transmittance
Wavenumbers (cm-1)

2k

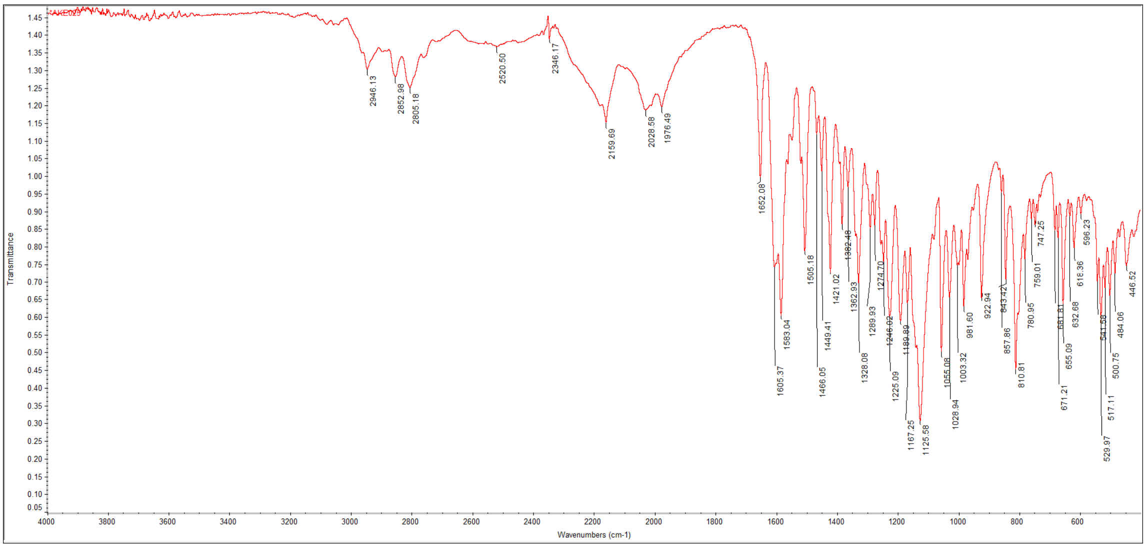
Transmittance
Wavenumbers (cm-1)

2l

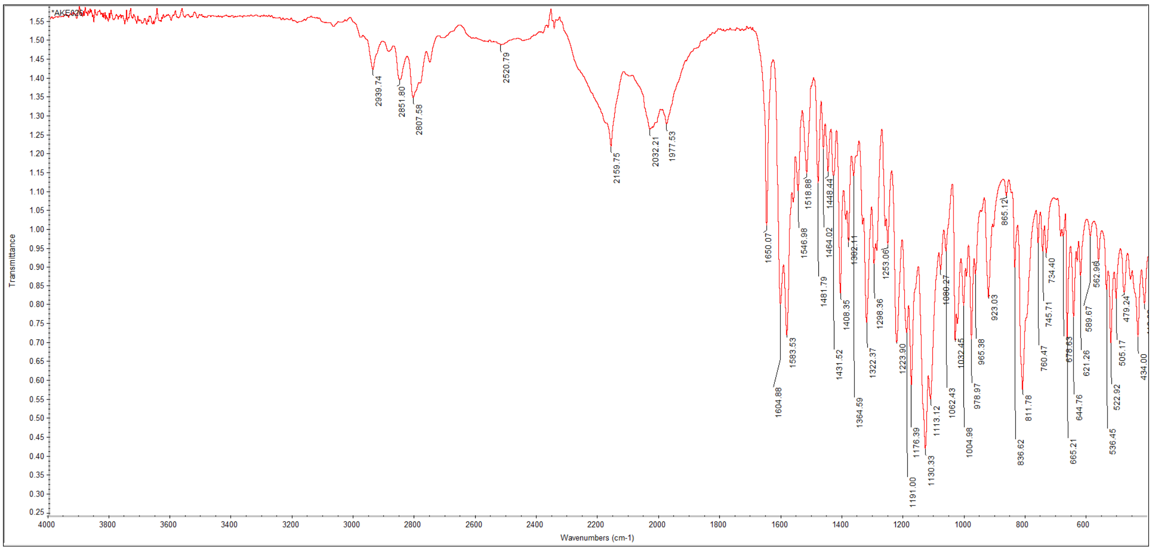
Transmittance
Wavenumbers (cm-1)

2m

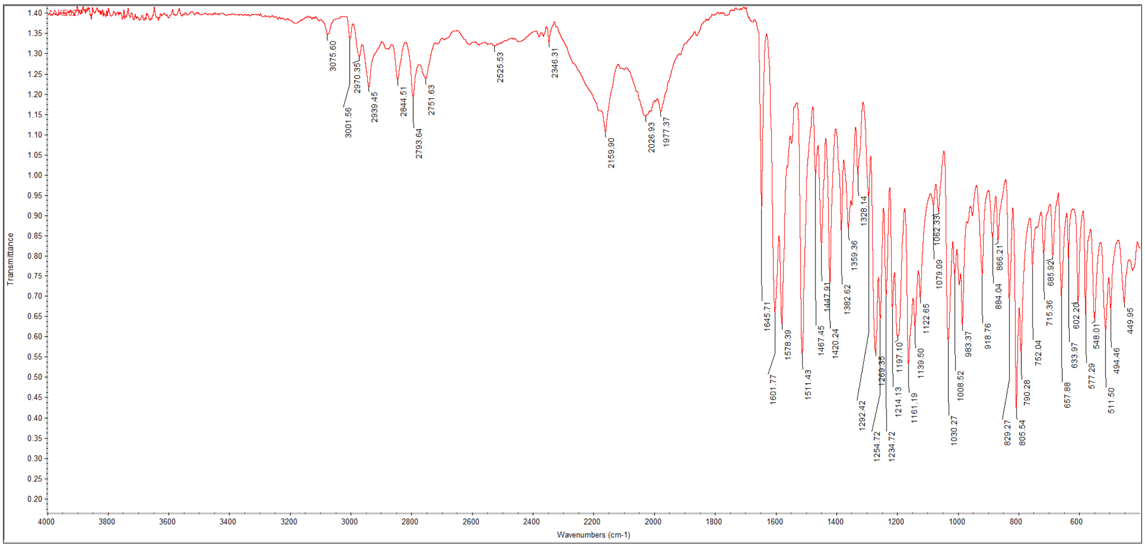

2n

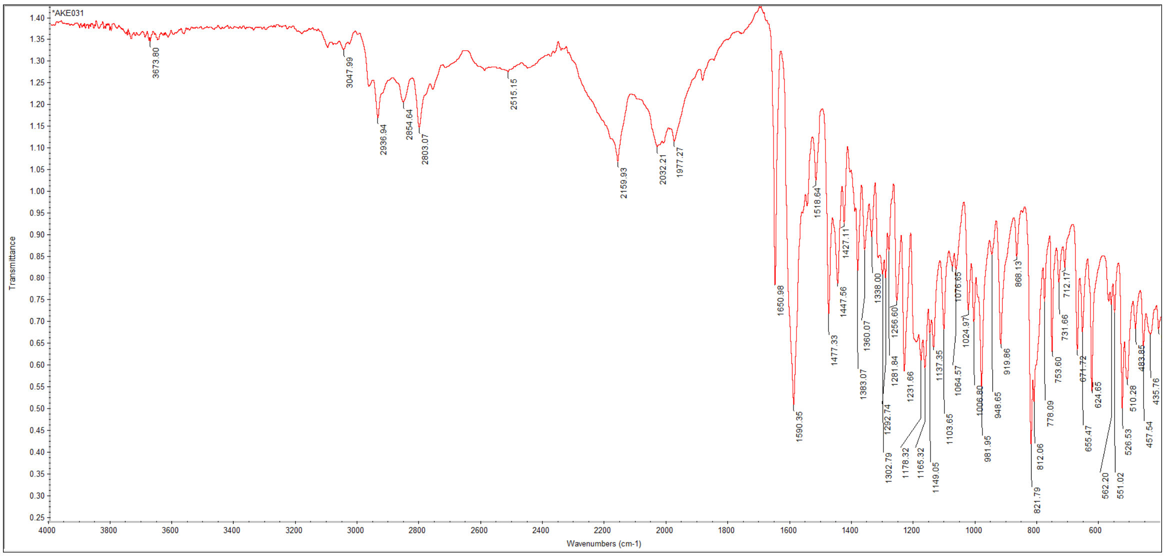

2o

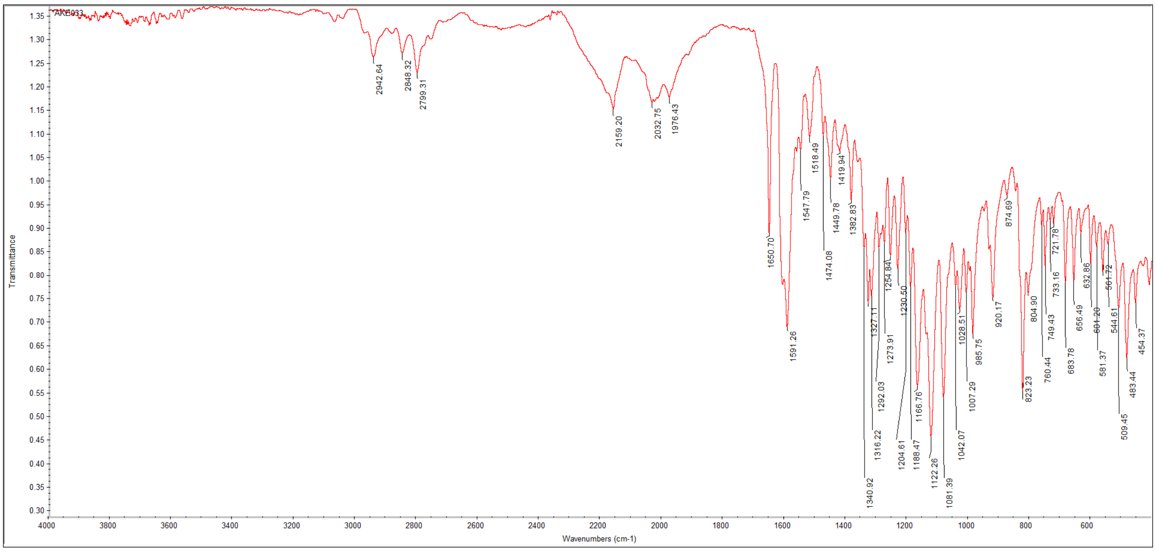
AKE033
Transmittance
Wavenumbers (cm-1)
2942.64
2848.32
2799.31
2159.20
2032.75
1976.43
1650.70
1591.26
1547.79
1518.49
1474.08
1449.78
1419.94
1382.83
1340.92
1327.11
1316.22
1292.03
1273.91
1254.84
1230.50
1204.61
1188.47
1166.76
1122.26
1081.39
1042.07
1028.51
1007.29
985.75
920.17
874.69
823.23
804.90
760.44
749.43
733.16
721.78
683.78
656.49
632.86
601.29
581.37
561.72
544.61
509.45
483.44
454.37

2p

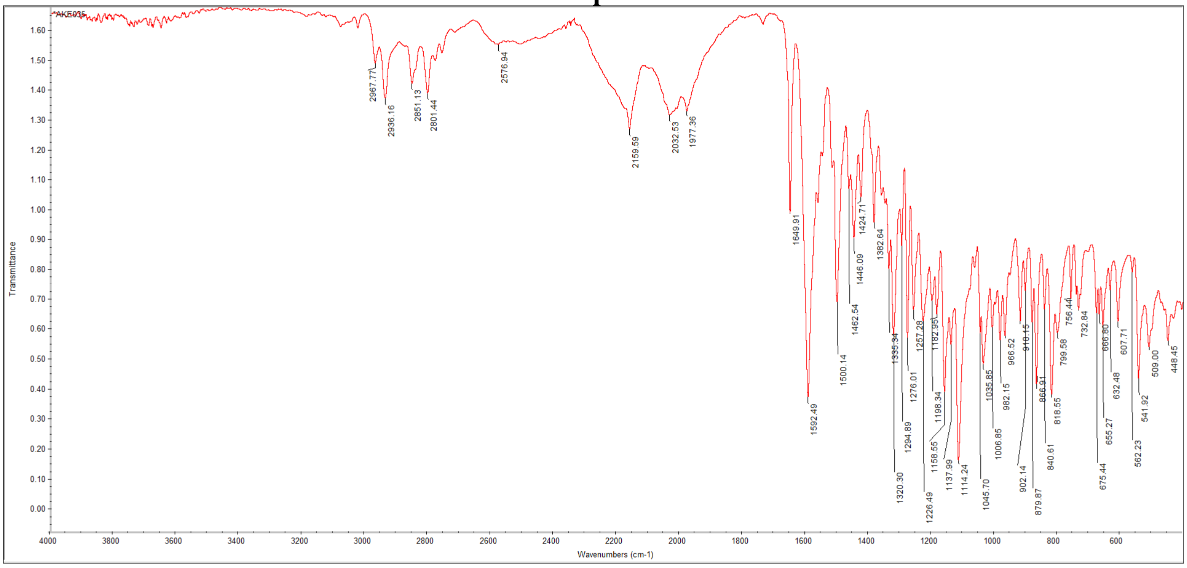
AKE035
Transmittance
Wavenumbers (cm-1)
2967.77
2936.16
2851.13
2801.44
2576.94
2159.59
2032.53
1977.36
1649.91
1592.49
1500.14
1462.54
1446.09
1424.71
1382.64
1335.34
1320.30
1294.89
1276.01
1257.28
1226.49
1198.34
1182.95
1158.55
1137.99
1114.24
1045.70
1035.85
1006.85
982.15
966.52
918.15
902.14
879.87
866.91
840.61
818.55
799.58
756.44
732.84
675.44
666.80
655.27
632.48
607.71
562.23
541.92
509.00
448.45

2q

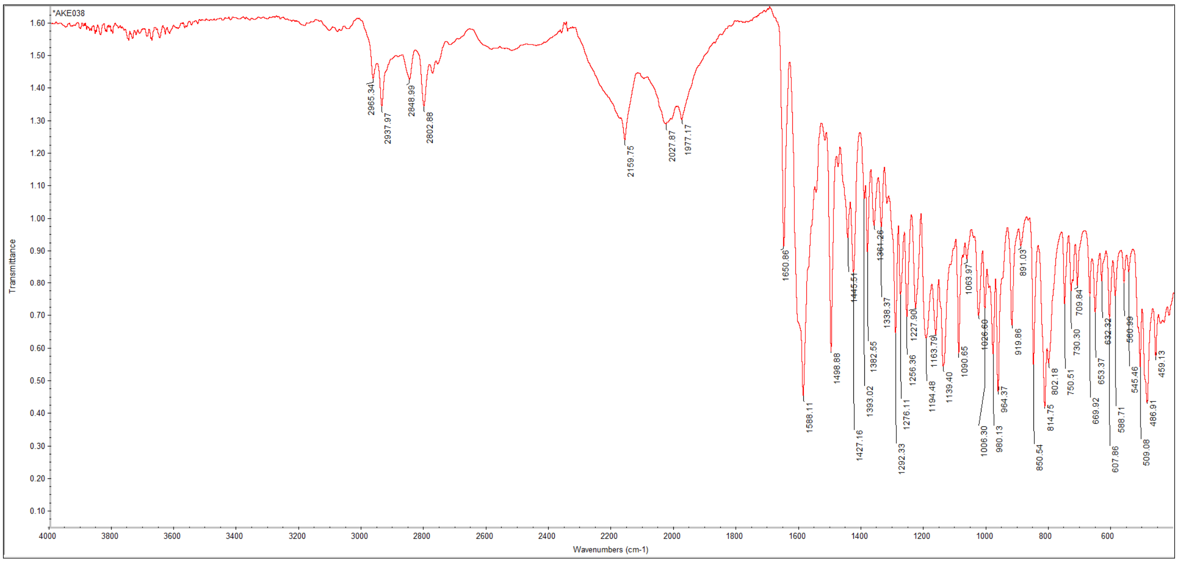
*AKE038
Transmittance
1.60
1.50
1.40
1.30
1.20
1.10
1.00
0.90
0.80
0.70
0.60
0.50
0.40
0.30
0.20
0.10
2965.34
2937.97
2848.99
2802.88
2159.75
2027.87
1977.17
1650.86
1588.11
1498.88
1445.51
1427.16
1393.02
1382.55
1361.26
1338.37
1292.33
1276.11
1256.36
1227.90
1194.48
1163.79
1139.40
1090.65
1063.97
1026.69
1006.30
980.13
964.37
919.86
891.03
850.54
814.75
802.18
750.51
730.30
709.84
669.92
653.37
632.32
607.86
588.71
560.99
545.46
509.08
486.91
459.13
4000
3800
3600
3400
3200
3000
2800
2600
2400
2200
2000
1800
1600
1400
1200
1000
800
600
Wavenumbers (cm-1)

Figure S4. IC$_{50}$ curves of 2k and 2n against MAO-B

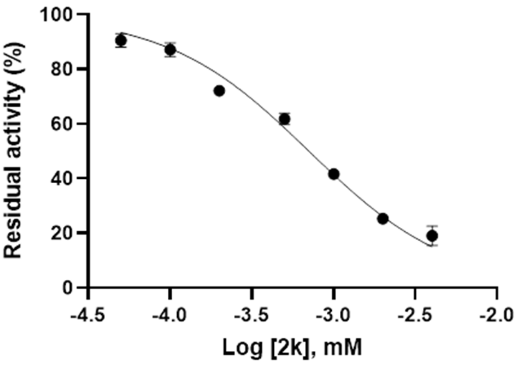

(**A**)

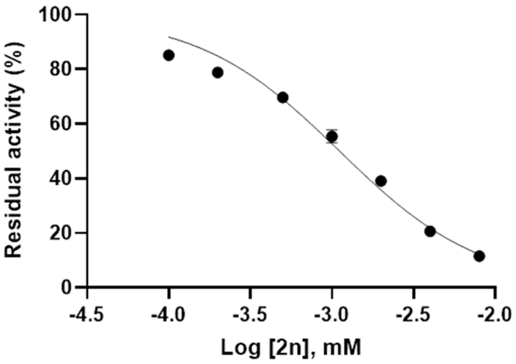

(**B**)

Figure S4. The IC$_{50}$ curves of **2k** (**A**) and **2n** (**B**) using GraphPad Prism version 9.3.1. The IC$_{50}$ of **2k** and **2n** were determined with 0.71 and 1.11 µM, respectively.
